# Supplementary material for: A Putative Prophylactic Solution for COVID-19: Development of Novel Multiepitope Vaccine Candidate against SARS-COV-2 by Comprehensive Immunoinformatic and Molecular Modelling Approach
Source: Biology (Basel). 2020 Sep 18;9(9):296. doi: 10.3390/biology9090296 (PMC7563440; doi:10.3390/biology9090296)
Supplement: Supplementary file 1 [file biology-09-00296-s001.pdf]

Supporting Information

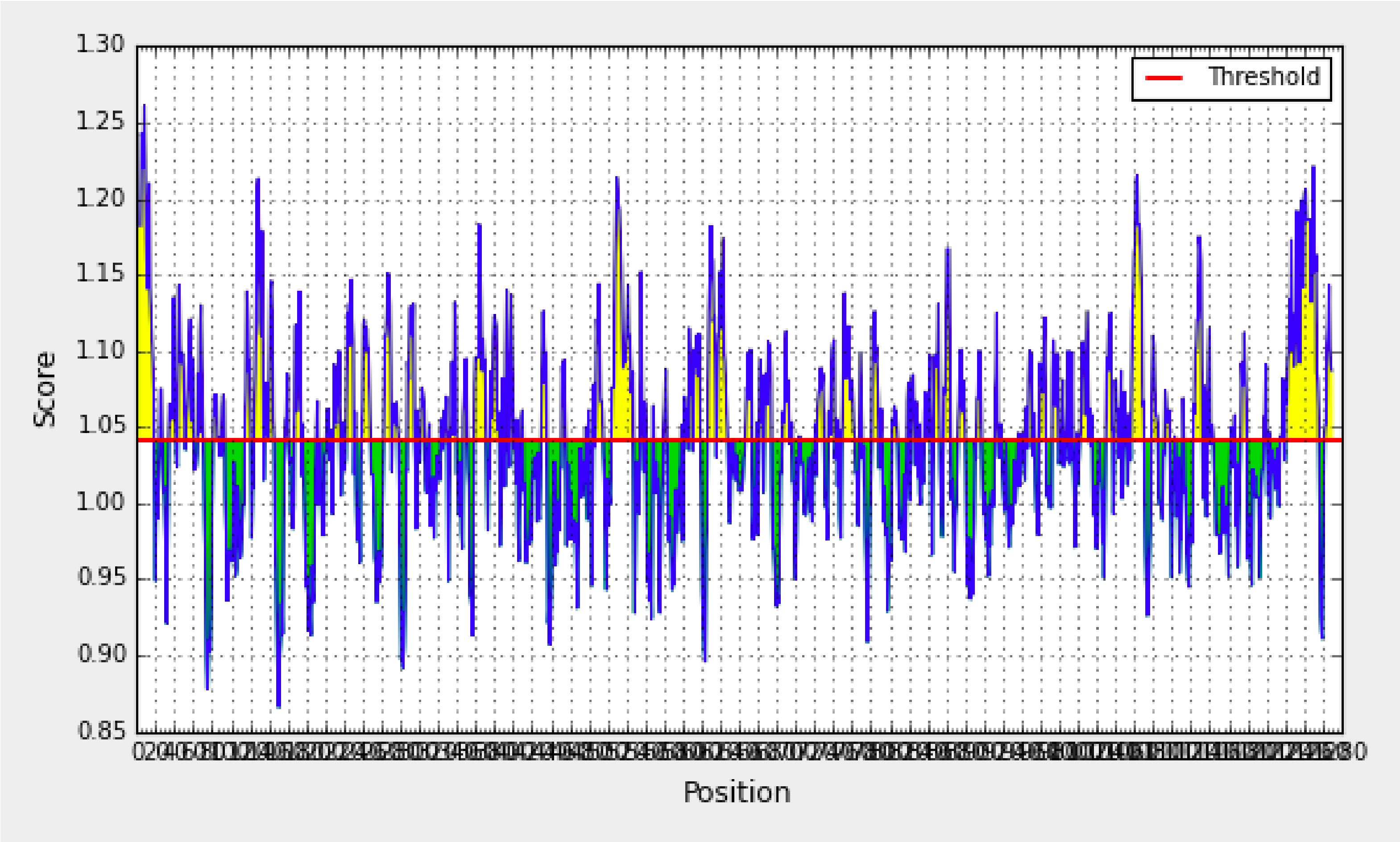

Figure S1. Graphical representation of predicted antigenic propensity of SARS-CoV-2 Spike protein.

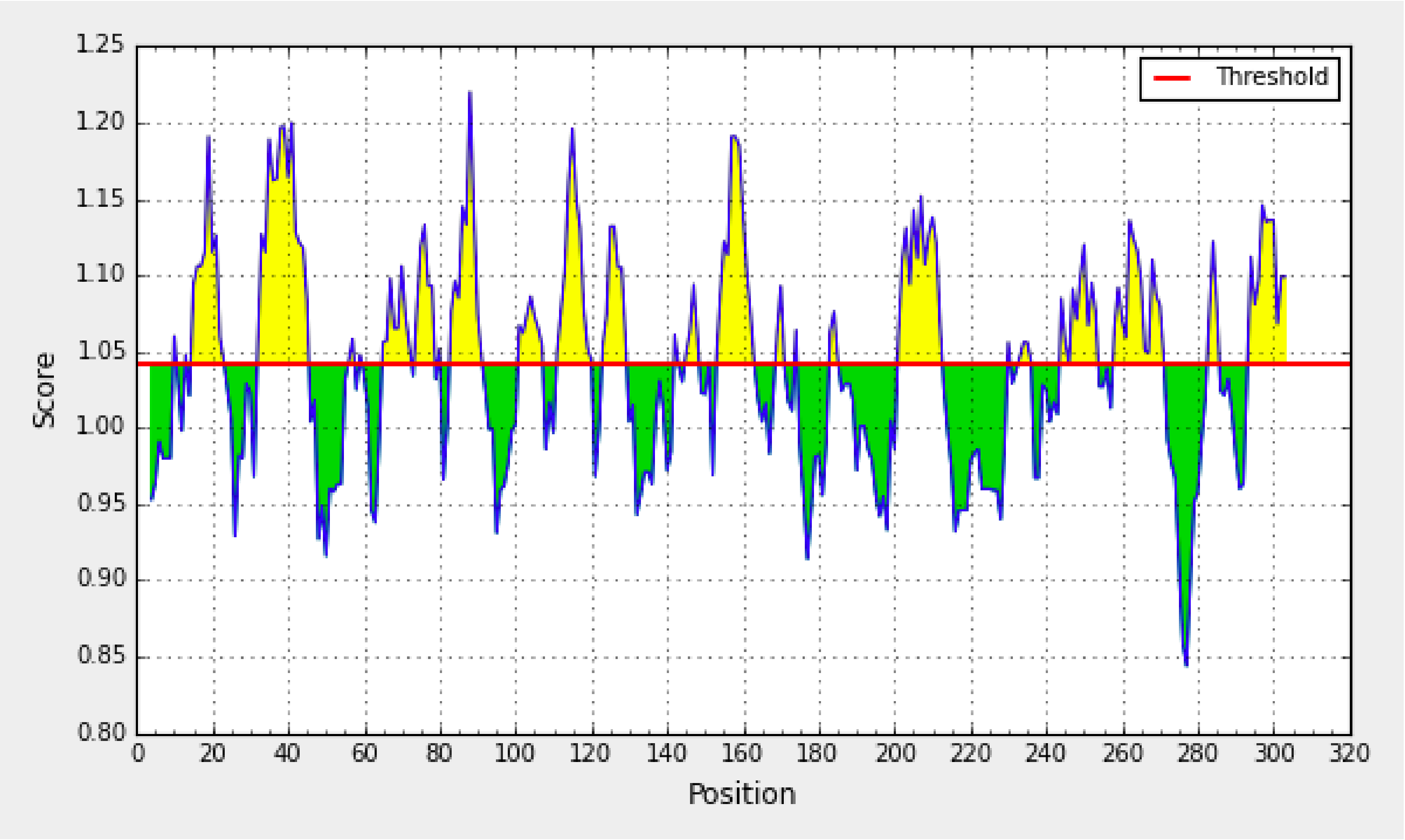

Figure S2. Graphical representation of predicted antigenic propensity of SARS-CoV-2 Main protease.

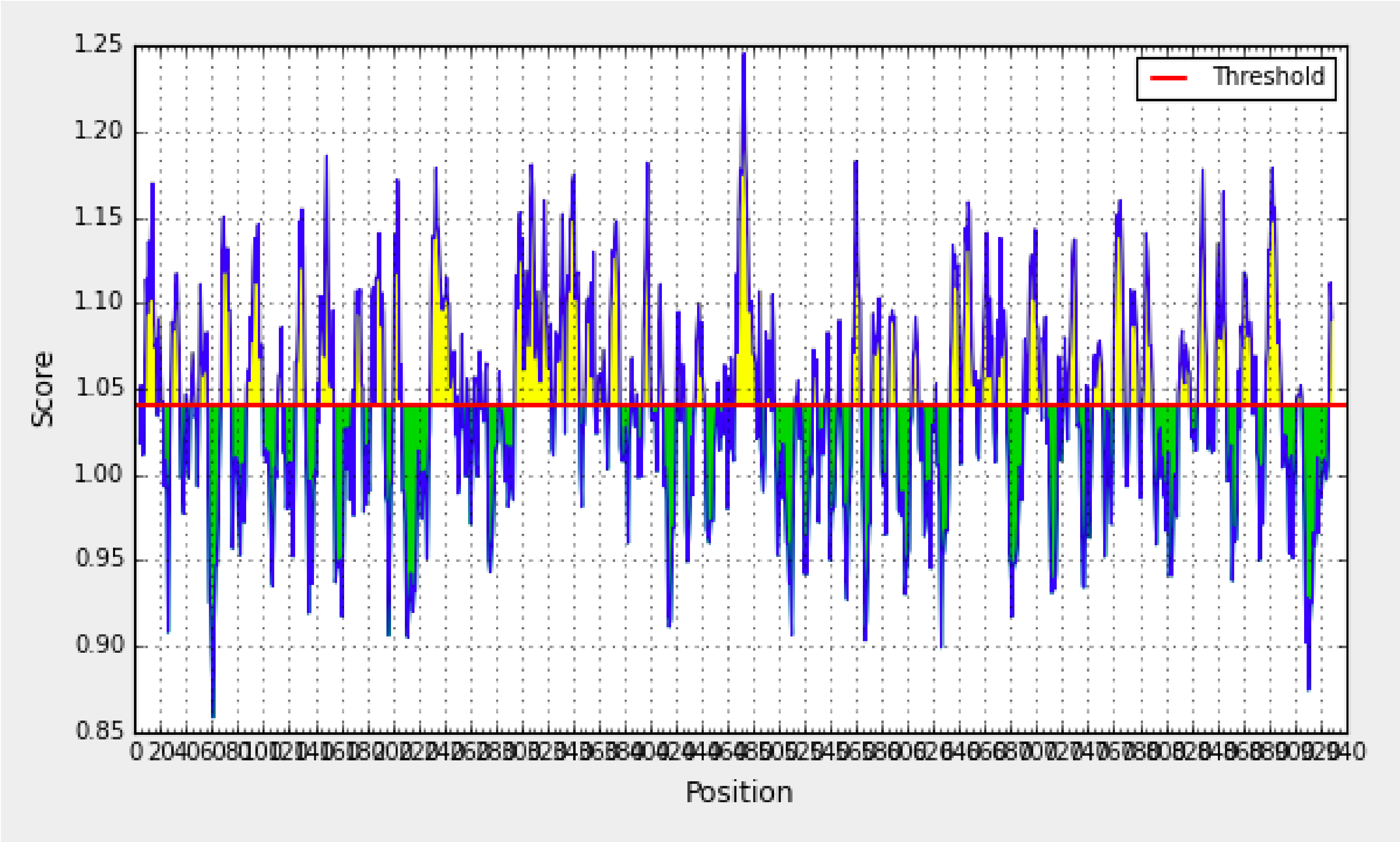

Figure S3. Graphical representation of predicted antigenic propensity of SARS-CoV-2 Nsp12 RNA-dependent-RNA-polymerase (RdRp).

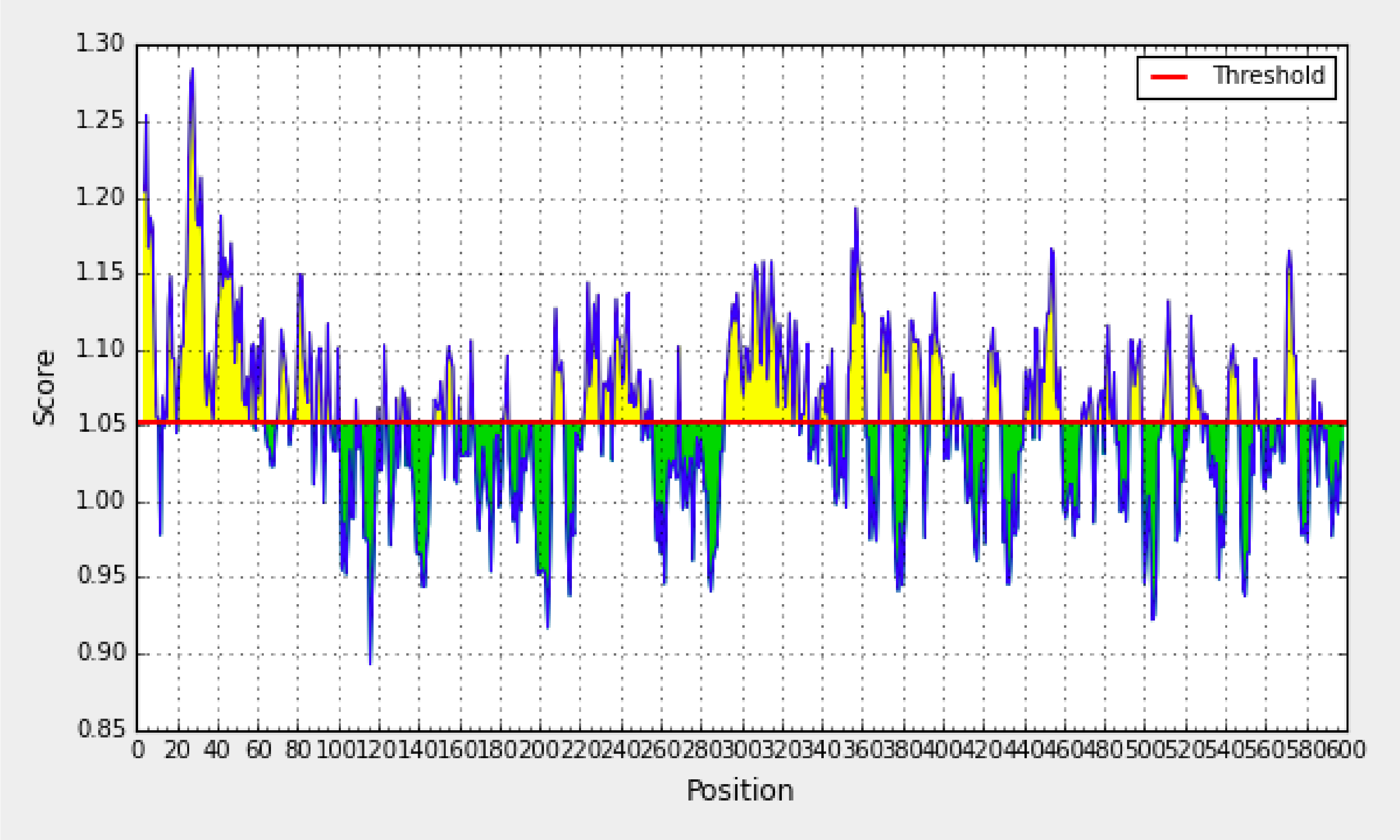

Figure S4. Graphical representation of predicted antigenic propensity of SARS-CoV-2 Nsp13 helicase.

GIGDPVTCLKSGAICHPVFCPRRYKQIGTCGLPGTKCCKKPEAAKCVADYSVLYAAYCNDPFLGVY AAY
MTSCCSCLKAAYSTQDLFLPFAAYTSNQVAVLYAAYKTSVDCTMYAAYVASQSIIAYAAYGAAAYYVGY AAY RISNCVADYAAYITDAVDCAL
AAYECSNLLLQY AAYTVNVLAWLYAAYQTFSVLACYAAY DYDCVSFCYAAYTANPKTPKYAAYDTDFVNEFY AAYLSFKELLVY
AAYSTDVVYRAFAAYFVSLAIDAYAAY
MVMCGGSLYAAYLSDDAVVCFAAYTTAYANSVFAAYAVVCFNSTYAAYMCDIRQLLFAAYVVDSYYSLLAAYVTANVNALLAAYMLVKQGDDYAA
YGDDYVYLPYAAYDVFHLYLQYAAYEYADVFHLYAAYGVPVVDSYYAAYSVELKHFFFAAYFVDGVPFVVAAYCCSLSHRFY AAYVTDVTQLYL
AAYDVTDVTQLYAAYSSQGSEYDY
AAYPTLVPQEHYAAYIVDTVSALVAAYCIMSDRDLYAAYKVNSTLEQYAAYHYVRITGLYAAYANGQVFGLYAAYGSEYDYVIFGPGPGVVVLSF
ELLHAPATVCGPK GPGPGITGRLQSLQTYVTQQLIRAAEIRGPGPG
AYYVGYLQPRTFLLKYNGPGPGTGRLQSLQTYVTQQLGPGPGIIAYTMSLGAENSVAYGPGPGGWTFGAGAALQIPFAGPGPGTPKYKFVRIQPG
QTFSVLAGPGPGSHNFLVQAGNVQLRVIGGPGPGFVRIQPGQTFSVLACGPGPGPEFYEAMYTPHTVLQGPGPGSLSHRFYRLANECAQVLSEGP
GPGQKLLKSIAATRGATVGPGPGWEPEFYEAMYTPHTVLGPGPGQFHQKLLKSIAATRGAGPGPGKLLKSIAATRGATVVGPGPGMPNMLRIMAS
LVLARKGPGPGAVASKILGLPTQTVDSSQGPGPGMQKYSTLQGPPGTGKSGPGPGETFKLSYGIATVREVLGPGPGVGDYFVLTSHTVMPLSGPG
PGISPYNSQNAVASKILEAAKGIINTLQKYYCRVRGGRCAVLSCLPKEEQIGKCSTRGRKCCRRKK

Figure S5. Multiepitope vaccine construct.

Note: Grey highlighted in start is human β-Defensins hBD-2 (PDB ID: 1FD3) and in the end human β-Defensins hBD-3 (PDB ID: 1KJ6). Yellow color represents CTL epitope while sea green color represents HTL epitope. The linker used are highlight red for 'AAY' and magenta for 'GPGPG'. First 11 yellow peptides belong to SARS-CoV-2 spike protein till ECSNLLLQY, next 4 peptide belong to Mpro till TANPKTPKY, next 19 peptides from RdRp till CCSLSHRFY followed by next 10 peptides from helicase till GSEYDYVIF. For HTL, First 6 sea green peptides belong to spike protein till GWTFGAGAALQIPFA, next 3 from Mpro till FVRIQPGQTFSVLAC, next 8 from RdRp till AVASKILGLPTQTVDSSQ followed by last 4 from helicase till SPYNSQNAVASKIL.

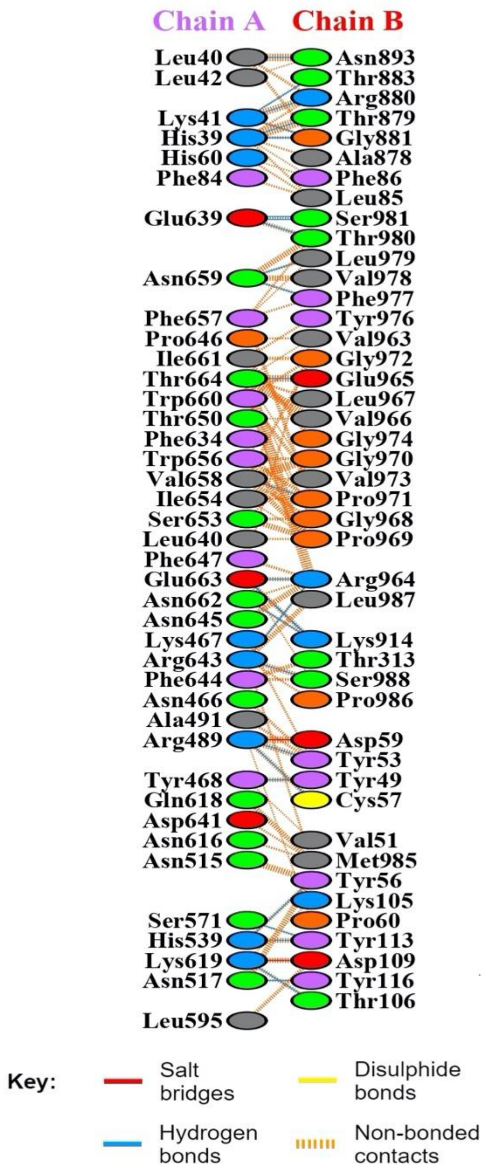

Figure S6. Molecular interactions of MVC (Chain B) with TLR3 (Chain A).

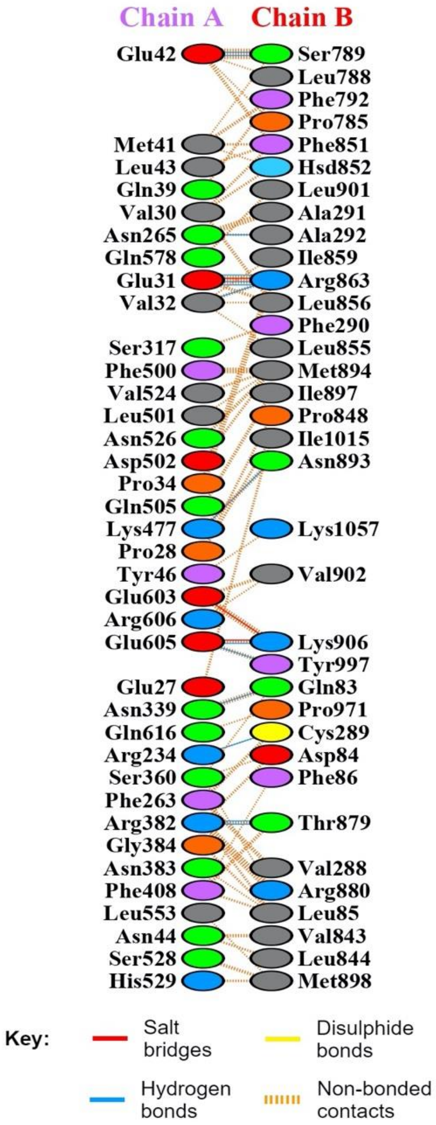

Figure S7. Molecular interactions of MVC (Chain B) with TLR4 (Chain A).

Table S1. List of predicted CTL from SARS-CoV-2 S protein. → -E represents the MHC ligands and top epitopes.

NetCTL-1.2 predictions using MHC supertype A1. Threshold 0.750000

Number of MHC ligands 37 identified. Number of peptides 1265. Protein name S

Residue No.	Peptide Sequence	Predicted MHC binding affinity	Rescale binding affinity	C-terminal cleavage affinity	Transport efficiency	Prediction score	Identified MHC ligand
865	LTDEMIAQY	0.7953	3.3768	0.9723	2.7790	3.6616	<-E
258	WTAGAAAYY	0.6735	2.8596	0.7339	2.8630	3.1128	<-E
604	TSNQVAVLY	0.6559	2.7847	0.9440	2.9910	3.0758	<-E
361	CVADYSVLY	0.5348	2.2705	0.9764	3.1800	2.5759	<-E
733	KTSVDCTMY	0.4908	2.0840	0.9649	3.0160	2.3795	<-E
746	STECSNLLL	0.5136	2.1808	0.8879	0.7030	2.3492	<-E
652	GAEHVNNSY	0.4042	1.7163	0.9769	2.6630	1.9960	<-E
196	NIDGYFKIY	0.3921	1.6649	0.9664	3.0150	1.9606	<-E
160	YSSANNCTF	0.3975	1.6878	0.9032	2.5980	1.9531	<-E
152	WMESEFRVY	0.3902	1.6569	0.7993	2.9290	1.9232	<-E
162	SANNCTFEY	0.3737	1.5865	0.9196	2.9900	1.8739	<-E
687	VASQSIIAY	0.3529	1.4986	0.9656	3.0890	1.7978	<-E
30	NSFTRGVYY	0.3389	1.4389	0.6421	3.1240	1.6915	<-E
136	CNDPFLGVY	0.2613	1.1095	0.6900	2.4500	1.3355	<-E
392	FTNVYADSF	0.2704	1.1480	0.3800	2.3170	1.3208	<-E
261	GAAAYYVGY	0.2253	0.9568	0.7608	2.9690	1.2194	<-E
357	RISNCVADY	0.2106	0.8941	0.9292	3.3940	1.2032	<-E
465	ERDISTEIY	0.2097	0.8903	0.9744	2.6460	1.1687	<-E
285	ITDAVDCAL	0.2350	0.9979	0.8708	0.7900	1.1680	<-E
1039	RVDFCGKGY	0.2036	0.8644	0.7618	3.2320	1.1403	<-E
343	NATRFASVY	0.1955	0.8300	0.9342	2.8730	1.1138	<-E
1237	MTSCCSCLK	0.2260	0.9595	0.7525	0.4790	1.0963	<-E
50	STQDLFLPF	0.1974	0.8383	0.5530	2.5110	1.0468	<-E
1096	VSNGTHWFV	0.2012	0.8544	0.6143	0.2180	0.9574	<-E
880	GTITSGWTF	0.1656	0.7031	0.7489	2.5570	0.9433	<-E
815	RSFIEDLLF	0.1421	0.6035	0.5938	3.0320	0.8441	<-E
1264	VLKGVKLHY	0.1262	0.5356	0.9783	2.8590	0.8253	<-E
748	ECSNLLLQY	0.1413	0.6000	0.5316	2.7470	0.8171	<-E
370	NSASFSTFK	0.1671	0.7093	0.5456	0.5070	0.8165	<-E

372	ASFSTFKCY	0.1180	0.5010	0.9587	3.2750	0.8085	<-E
628	QLTPTWRVY	0.1189	0.5047	0.9661	2.7820	0.7887	<-E
296	LSETKCTLK	0.1515	0.6432	0.8919	0.2200	0.7879	<-E
192	FVFKNIDGY	0.1358	0.5767	0.4093	2.9130	0.7837	<-E
445	VGGNYNYLY	0.1164	0.4941	0.9518	2.6580	0.7698	<-E
83	VLPFNDGVY	0.1130	0.4797	0.9703	2.8460	0.7675	<-E
1095	FVSNGTHWF	0.1232	0.5231	0.7203	2.6210	0.7622	<-E
612	YQDVNCTEV	0.1531	0.6501	0.5870	0.2420	0.7502	<-E
554	ESNKKFLPF	0.1137	0.4828	0.8860	2.2870	0.7300	
1054	QSAPHGVVF	0.1298	0.5509	0.3317	2.4600	0.7237	
584	ILDITPCSF	0.1091	0.4633	0.9625	2.2340	0.7194	
240	TLLALHRSY	0.1004	0.4261	0.9765	2.8920	0.7171	
497	FQPTNGVGY	0.1117	0.4741	0.5919	2.8600	0.7059	
109	TLDSKTQSL	0.1226	0.5207	0.9659	0.7870	0.7049	
482	GVEGFNCYF	0.1113	0.4727	0.7156	2.4910	0.7046	
1059	GVVFLHVTY	0.0965	0.4098	0.9743	2.9390	0.7029	
94	STEKSNIIR	0.1454	0.6174	0.0573	1.5260	0.7023	
603	NTSNQVAVL	0.1153	0.4893	0.9311	0.9760	0.6778	
28	YTNSFTRGV	0.1446	0.6140	0.2606	0.3330	0.6697	
940	STASALGKL	0.1169	0.4965	0.7662	1.0570	0.6643	
441	LDSKVGGNY	0.1029	0.4370	0.5479	2.7580	0.6571	
51	TQDLFLPFF	0.1126	0.4782	0.3252	2.4670	0.6503	
269	YLQPRTFLL	0.1065	0.4522	0.9774	0.8920	0.6434	
625	HADQLTPTW	0.1085	0.4609	0.9481	0.6790	0.6370	
699	LGAENSVAY	0.0829	0.3518	0.9565	2.7330	0.6320	
869	MIAQYTSAL	0.1043	0.4427	0.9295	0.9800	0.6311	
369	YNSASFSTF	0.0862	0.3659	0.9425	2.4060	0.6276	
781	VFAQVKQIY	0.0787	0.3339	0.8989	3.0040	0.6190	
1248	CSCGSCCKF	0.0828	0.3516	0.9216	2.5360	0.6166	
983	RLDKVEAEV	0.1044	0.4432	0.9691	0.4560	0.6113	
723	TTEILPVSM	0.1189	0.5049	0.6408	0.1650	0.6093	
973	ISSVLNDIL	0.1146	0.4866	0.4188	1.1680	0.6078	
443	SKVGGNYNY	0.0737	0.3129	0.9617	2.9660	0.6054	
413	GQTGKIADY	0.0756	0.3210	0.9727	2.6400	0.5989	
909	IGVTQNVLY	0.0760	0.3227	0.9090	2.7290	0.5955	
718	FTISVTTEI	0.1091	0.4634	0.6625	0.6280	0.5941	
334	NLCPFGEVF	0.0778	0.3303	0.8576	2.6100	0.5894	
292	ALDPLSETK	0.0979	0.4158	0.9698	0.4450	0.5835	
617	CTEVPVAIH	0.1406	0.5971	0.0786	-0.6010	0.5789	
78	RFDNPVLPF	0.0737	0.3127	0.8082	2.8710	0.5775	
12	SSQCVNLTT	0.1314	0.5577	0.3085	-0.5970	0.5742	

271	QPRTFLLKY	0.0679	0.2884	0.9734	2.7300	0.5709
572	TTDAVRDPQ	0.1390	0.5902	0.0248	-0.4630	0.5707
305	SFTVEKGIY	0.0689	0.2927	0.7809	3.2100	0.5703
1144	ELDSFKEEL	0.0996	0.4228	0.8267	0.4490	0.5693
69	HVSGTNGTK	0.0937	0.3978	0.9687	0.5170	0.5690
1113	QIITTDNTF	0.0761	0.3232	0.7536	2.6000	0.5663
171	VSQPFLMDL	0.0871	0.3697	0.9711	0.9730	0.5640
29	TNSFTRGVY	0.0801	0.3402	0.5887	2.6950	0.5632
1005	QTYVTQQLI	0.0931	0.3951	0.8470	0.7610	0.5602
125	NVVIKVCEF	0.0644	0.2734	0.9725	2.7980	0.5591
937	SLSSTASAL	0.0876	0.3718	0.9485	0.8840	0.5583
721	SVTTEILPV	0.0922	0.3913	0.9374	0.4790	0.5559
898	FAMQMAYRF	0.0951	0.4037	0.1975	2.4120	0.5539
417	KIADYNYKL	0.0821	0.3485	0.9666	1.1770	0.5524
773	EQDKNTQEV	0.1041	0.4419	0.7161	0.0570	0.5522
751	NLLLQYGSF	0.0654	0.2777	0.9341	2.6630	0.5510
1020	ASANLAATK	0.0957	0.4064	0.6962	0.7980	0.5507
20	TRTQLPPAY	0.0594	0.2522	0.9524	3.0840	0.5493
710	NSIAIPTNF	0.0789	0.3349	0.4789	2.8220	0.5478
1102	WFVTQRNFY	0.0767	0.3257	0.4336	3.1200	0.5468
777	NTQEVFAQV	0.0903	0.3835	0.9215	0.3990	0.5416
874	TSALLAGTI	0.1160	0.4925	0.1409	0.5190	0.5396
635	VYSTGSNVF	0.0575	0.2443	0.9721	2.9890	0.5395
734	TSVDCTMYI	0.0844	0.3582	0.9493	0.7330	0.5373
735	SVDCTMYIC	0.1244	0.5283	0.0323	0.0400	0.5352
388	NDLCFTNVY	0.0620	0.2631	0.8904	2.7200	0.5327
576	VRDPQTLEI	0.0847	0.3597	0.8800	0.7810	0.5308
59	FSNVTWFHA	0.1311	0.5566	0.0786	-0.7890	0.5289
366	SVLYNSASF	0.0597	0.2533	0.9328	2.6920	0.5278
999	GRLQSLQTY	0.0550	0.2333	0.9621	2.9800	0.5266
257	GWTAGAAAY	0.0573	0.2432	0.8692	3.0100	0.5241
288	AVDCALDPL	0.0827	0.3513	0.8416	0.9220	0.5236
396	YADSFVIRG	0.1314	0.5579	0.2678	-1.4920	0.5235
827	TLADAGFIK	0.0833	0.3536	0.9482	0.5270	0.5222
704	SVAYSNNSI	0.0891	0.3782	0.6585	0.8840	0.5212
1099	GTHWFVTQR	0.0723	0.3071	0.9418	1.4550	0.5212
1130	IGIVNNTVY	0.0562	0.2387	0.9441	2.7970	0.5202
489	YFPLQSYGF	0.0577	0.2450	0.9343	2.6600	0.5181
896	IPFAMQMAY	0.0558	0.2371	0.9698	2.7060	0.5179
829	ADAGFIKQY	0.0541	0.2297	0.9677	2.8260	0.5162
204	YSKHTPINL	0.0765	0.3248	0.9372	0.9820	0.5145

675	QTQTNSPRR	0.0757	0.3213	0.8208	1.3920	0.5141
551	VLTESNKKF	0.0598	0.2537	0.8814	2.5500	0.5134
487	NCYFPLQSY	0.0507	0.2153	0.9572	3.0640	0.5121
481	NGVEGFNCY	0.0546	0.2318	0.9381	2.7660	0.5109
1147	SFKEELDKY	0.0518	0.2199	0.9375	3.0040	0.5107
691	SIIAYTMSL	0.0729	0.3095	0.9516	1.1560	0.5100
886	WTFGAGAAL	0.0794	0.3369	0.8225	0.9880	0.5097
234	NITRFQTLL	0.0736	0.3123	0.9498	1.0930	0.5094
262	AAAYYVGYL	0.0847	0.3597	0.6133	1.1380	0.5086
666	IGAGICASY	0.0550	0.2335	0.9760	2.5530	0.5075
533	LVKNKCVNF	0.0530	0.2251	0.9372	2.8160	0.5065
745	DSTECSNLL	0.0860	0.3652	0.7136	0.6130	0.5029
433	VIAWNSNNL	0.0718	0.3048	0.9336	1.1550	0.5026
62	VTWFHAIHV	0.0768	0.3259	0.9521	0.6680	0.5021
794	IKDFGGFNF	0.0605	0.2571	0.8688	2.2860	0.5017
636	YSTGSNVFQ	0.1172	0.4978	0.0487	-0.0740	0.5014
965	QLSSNFGAI	0.0819	0.3476	0.8031	0.6640	0.5013
437	NSNNLDSKV	0.1043	0.4428	0.3000	0.2420	0.4999
382	VSPTKLNDL	0.0747	0.3172	0.8742	1.0120	0.4989
2	FVFLVLLPL	0.0703	0.2985	0.9532	1.1460	0.4988
11	VSSQCVNLT	0.1203	0.5108	0.1022	-0.5590	0.4982
1173	NASVVNIQK	0.0772	0.3278	0.9493	0.5590	0.4982
24	LPPAYTNSF	0.0576	0.2445	0.9581	2.1700	0.4967
47	VLHSTQDLF	0.0804	0.3412	0.1327	2.7000	0.4961
1116	TTDNTFVSG	0.1254	0.5324	0.2933	-1.6050	0.4961
509	RVVVLSFEL	0.0669	0.2842	0.9559	1.3630	0.4957
552	LTESNKKFL	0.1014	0.4304	0.1506	0.8400	0.4950
789	YKTPPIKDF	0.0543	0.2304	0.9165	2.5370	0.4947
241	LLALHRSYL	0.0721	0.3063	0.9122	1.0050	0.4933
415	TGKIADYNY	0.0513	0.2179	0.9511	2.5860	0.4899
557	KKFLPFQQF	0.0467	0.1984	0.9567	2.9440	0.4891
1129	VIGIVNNTV	0.0772	0.3276	0.9469	0.3890	0.4891
992	QIDRLITGR	0.0740	0.3144	0.6632	1.4970	0.4887
939	SSTASALGK	0.1012	0.4295	0.2039	0.5700	0.4886
500	TNGVGYQPY	0.0675	0.2865	0.5291	2.4430	0.4880
956	AQALNTLVK	0.0707	0.3003	0.9664	0.8420	0.4874
321	QPTESIVRF	0.0548	0.2328	0.9422	2.2540	0.4868
1226	AIVMVTIML	0.0660	0.2802	0.9324	1.3140	0.4857
1201	QELGKYEQY	0.0617	0.2619	0.5295	2.8680	0.4847
1197	LIDLQELGK	0.0900	0.3821	0.5236	0.4750	0.4844
122	NATNVVIKV	0.0748	0.3177	0.9412	0.5090	0.4843

347	FASVYAWNR	0.0921	0.3911	0.0966	1.5700	0.4841
976	VLNDILSRL	0.0677	0.2876	0.9706	0.9960	0.4830
205	SKHTPINLV	0.0754	0.3202	0.9458	0.3950	0.4818
607	QVAVLYQDV	0.0851	0.3613	0.5945	0.6240	0.4817
137	NDPFLGVYY	0.0523	0.2221	0.8504	2.6320	0.4813
127	VIKVCEFQF	0.0504	0.2140	0.8496	2.7700	0.4799
178	DLEGKQGNF	0.0694	0.2947	0.5170	2.1300	0.4787
1192	NLNESLIDL	0.0671	0.2847	0.9460	1.0180	0.4775
98	SNIIRGWIF	0.0580	0.2463	0.6679	2.6050	0.4767
478	TPCNGVEGF	0.0559	0.2372	0.8306	2.2720	0.4754
1224	LIAIVMVTI	0.0754	0.3200	0.8161	0.6540	0.4751
93	ASTEKSNII	0.0774	0.3288	0.7772	0.5780	0.4743
193	VFKNIDGYF	0.0494	0.2099	0.8749	2.6570	0.4740
84	LPFNDGVYF	0.0488	0.2071	0.9761	2.3930	0.4732
230	PIGINITRF	0.0560	0.2379	0.8777	2.0680	0.4729
1212	WPWYIWLGF	0.0521	0.2214	0.8493	2.4760	0.4726
1021	SANLAATKM	0.0719	0.3051	0.9593	0.4700	0.4725
975	SVLNDILSR	0.0640	0.2716	0.7533	1.7480	0.4720
302	TLKSFTVEK	0.0718	0.3046	0.9616	0.4540	0.4716
111	DSKTQSLLI	0.1013	0.4301	0.1747	0.3050	0.4715
882	ITSGWTFGA	0.0879	0.3730	0.8194	-0.5280	0.4695
1065	VTYVPAQEK	0.0695	0.2951	0.9318	0.6690	0.4683
1000	RLQSLQTYV	0.0778	0.3303	0.7484	0.5160	0.4683
697	MSLGAENSV	0.0721	0.3060	0.9348	0.4300	0.4677
1207	EQYIKWPWY	0.0693	0.2942	0.1715	2.9310	0.4665
505	YQPYRVVVL	0.0642	0.2724	0.9714	0.9650	0.4664
167	TFEYVSQPF	0.0524	0.2226	0.7808	2.5300	0.4663
689	SQSIIAYTM	0.0688	0.2923	0.9649	0.5830	0.4662
212	LVRDLPQGF	0.0515	0.2186	0.6949	2.8670	0.4662
469	STEIYQAGS	0.1360	0.5773	0.0385	-2.3490	0.4656
510	VVVLSFELL	0.0655	0.2780	0.7954	1.3650	0.4655
828	LADAGFIKQ	0.0911	0.3867	0.5884	-0.2150	0.4642
454	RLFRKSNLK	0.0667	0.2832	0.9440	0.7880	0.4642
559	FLPFQQFGR	0.0607	0.2576	0.8766	1.4950	0.4639
719	TISVTTEIL	0.0747	0.3172	0.6591	0.9530	0.4638
10	LVSSQCVNL	0.0638	0.2709	0.9683	0.9480	0.4636
35	GVYYPDKVF	0.0447	0.1897	0.8852	2.8130	0.4631
71	SGTNGTKRF	0.0505	0.2143	0.8804	2.3320	0.4630
384	PTKLNDLCF	0.0776	0.3295	0.1894	2.0850	0.4621
444	KVGGNYNYL	0.0633	0.2689	0.9132	1.0910	0.4605
972	AISSVLNDI	0.0711	0.3019	0.7989	0.7730	0.4604

568	DIADTTDAV	0.0745	0.3164	0.8618	0.2760	0.4594
236	TRFQTLLAL	0.0588	0.2497	0.9752	1.2610	0.4590
1114	IITTDNTFV	0.0765	0.3250	0.7566	0.3810	0.4575
538	CVNFNFNGL	0.0638	0.2710	0.8885	1.0590	0.4572
456	FRKSNLKPF	0.0448	0.1904	0.8815	2.6920	0.4572
955	NAQALNTLV	0.0952	0.4040	0.2436	0.3230	0.4567
930	AIGKIQDSL	0.0643	0.2730	0.8507	1.1180	0.4565
46	SVLHSTQDL	0.0603	0.2558	0.9287	1.2180	0.4561
374	FSTFKCYGV	0.0780	0.3310	0.7351	0.2780	0.4551
825	KVTLADAGF	0.0584	0.2477	0.4133	2.8850	0.4540
958	ALNTLVKQL	0.0621	0.2637	0.9430	0.9750	0.4539
712	IAIPTNFTI	0.0643	0.2732	0.9592	0.7330	0.4537
507	PYRVVVLSF	0.0452	0.1918	0.9718	2.3190	0.4536
870	IAQYTSALL	0.0654	0.2778	0.8103	1.0340	0.4510
1016	AEIRASANL	0.0581	0.2467	0.9642	1.1860	0.4506
1236	CMTSCCSCL	0.0629	0.2673	0.8906	0.9920	0.4505
923	IANQFNSAI	0.0759	0.3224	0.6619	0.5640	0.4499
270	LQPRTFLLK	0.0741	0.3146	0.7358	0.4770	0.4488
1055	SAPHGVVFL	0.0615	0.2611	0.9181	0.9960	0.4486
535	KNKCVNFNF	0.0489	0.2077	0.7576	2.5340	0.4481
409	QIAPGQTGK	0.0693	0.2941	0.7750	0.7550	0.4481
1185	RLNEVAKNL	0.0595	0.2527	0.9655	0.9850	0.4467
778	TQEVFAQVK	0.0710	0.3013	0.7963	0.5160	0.4465
1198	IDLQELGKY	0.0537	0.2279	0.5528	2.7060	0.4461
762	QLNRALTGI	0.0703	0.2987	0.8209	0.4830	0.4460
638	TGSNVFQTR	0.0593	0.2519	0.8763	1.2470	0.4457
919	NQKLIANQF	0.0523	0.2219	0.6627	2.4780	0.4452
400	FVIRGDEVR	0.0615	0.2612	0.6768	1.6510	0.4452
1125	NCDVVIGIV	0.0964	0.4095	0.1801	0.1660	0.4448
229	LPIGINITR	0.0579	0.2459	0.9513	1.1140	0.4443
44	RSSVLHSTQ	0.0967	0.4105	0.1986	0.0700	0.4437
425	LPDDFTGCV	0.0734	0.3117	0.8962	-0.0610	0.4431
1140	PLQPELDSF	0.0513	0.2176	0.8656	1.9030	0.4426
202	KIYSKHTPI	0.0601	0.2552	0.9616	0.8520	0.4421
1209	YIKWPWYIW	0.0645	0.2737	0.8066	0.9420	0.4418
1169	ISGINASVV	0.0809	0.3434	0.6285	0.0760	0.4414
792	PPIKDFGGF	0.0482	0.2048	0.9607	1.8290	0.4404
755	QYGSFCTQL	0.0560	0.2377	0.9740	1.1170	0.4396
102	RGWIFGTTL	0.0560	0.2377	0.9747	1.1130	0.4396
878	LAGTITSGW	0.0643	0.2732	0.8282	0.8260	0.4387
853	QKFNGLTVL	0.0556	0.2359	0.9238	1.2830	0.4387

1137	VYDPLQPEL	0.0557	0.2366	0.9745	1.1150	0.4385
458	KSNLKPFER	0.0604	0.2566	0.6559	1.6700	0.4384
119	IVNNATNVV	0.0756	0.3211	0.6391	0.4300	0.4384
926	QFNSAIGKI	0.0619	0.2627	0.9136	0.7620	0.4379
206	KHTPINLVR	0.0500	0.2121	0.9666	1.6170	0.4379
569	IADTTDAVR	0.0733	0.3111	0.4273	1.2450	0.4374
818	IEDLLFNKV	0.0701	0.2977	0.9568	-0.0790	0.4373
674	YQTQTNSPR	0.0641	0.2723	0.5812	1.5580	0.4373
1067	YVPAQEKNF	0.0610	0.2591	0.3043	2.6480	0.4372
215	DLPQGFSAL	0.0592	0.2513	0.9770	0.7750	0.4366
987	VEAEVQIDR	0.0615	0.2610	0.6581	1.5290	0.4362
453	YRLFRKSNL	0.0551	0.2340	0.9416	1.2070	0.4356
1060	VVFLHVTYV	0.0669	0.2839	0.8122	0.5970	0.4355
327	VRFPNITNL	0.0521	0.2212	0.9763	1.3440	0.4348
149	NKSWMESEF	0.0604	0.2563	0.2689	2.7580	0.4346
1087	AHFPREGVF	0.0430	0.1825	0.7109	2.8990	0.4341
57	PFFSNVTWF	0.0443	0.1882	0.8376	2.3820	0.4330
221	SALEPLVDL	0.0587	0.2493	0.8963	0.9730	0.4324
996	LITGRLQSL	0.0563	0.2389	0.8918	1.1830	0.4318
764	NRALTGIAV	0.0618	0.2624	0.9218	0.6230	0.4318
394	NVYADSFVI	0.0577	0.2450	0.9417	0.9070	0.4316
1101	HWFVTQRNF	0.0484	0.2056	0.5686	2.8120	0.4315
873	YTSALLAGT	0.1099	0.4668	0.0296	-0.8000	0.4312
233	INITRFQTL	0.0588	0.2499	0.9106	0.8640	0.4296
908	GIGVTQNVL	0.0572	0.2429	0.9495	0.8820	0.4294
447	GNYNYLYRL	0.0576	0.2446	0.9709	0.7800	0.4292
846	ARDLICAQK	0.0575	0.2439	0.9601	0.7930	0.4276
312	IYQTSNFRV	0.0621	0.2638	0.9118	0.5400	0.4276
1181	KEIDRLNEV	0.0633	0.2686	0.9569	0.3060	0.4274
1081	ICHDGKAHF	0.0507	0.2154	0.4924	2.7620	0.4273
988	EAEVQIDRL	0.0746	0.3169	0.5000	0.7060	0.4272
915	VLYENQKLI	0.0588	0.2495	0.8669	0.9440	0.4267
267	VGYLQPRTF	0.0483	0.2050	0.6564	2.4650	0.4267
962	LVKQLSSNF	0.0605	0.2571	0.2284	2.6850	0.4256
75	GTKRFDNPV	0.0740	0.3142	0.6561	0.2530	0.4252
590	CSFGGVSVI	0.0648	0.2752	0.8244	0.5010	0.4239
732	TKTSVDCTM	0.0620	0.2631	0.9589	0.3330	0.4236
857	GLTVLPPLL	0.0582	0.2470	0.8748	0.9000	0.4232
89	GVYFASTEK	0.0615	0.2609	0.9201	0.4720	0.4226
817	FIEDLLFNK	0.0779	0.3307	0.4739	0.4080	0.4222
1175	SVVNIQKEI	0.0630	0.2674	0.7072	0.9580	0.4214

26	PAYTNSFTR	0.0579	0.2457	0.8047	1.0990	0.4213
348	ASVYAWNRK	0.0776	0.3295	0.4040	0.6220	0.4212
627	DQLTPTWRV	0.0653	0.2774	0.8745	0.2460	0.4209
550	GVLTESNKK	0.0611	0.2594	0.9243	0.4520	0.4206
725	EILPVSMTK	0.0598	0.2540	0.9693	0.4170	0.4202
847	RDLICAQKF	0.0470	0.1996	0.5857	2.6430	0.4196
345	TRFASVYAW	0.0487	0.2070	0.9724	1.3360	0.4196
634	RVYSTGSNV	0.0668	0.2838	0.6279	0.8280	0.4194
238	FQTLLALHR	0.0644	0.2735	0.4490	1.5610	0.4189
421	YNYKLPDDF	0.0644	0.2733	0.1636	2.4100	0.4184
319	RVQPTESIV	0.0651	0.2765	0.7573	0.5600	0.4181
894	LQIPFAMQM	0.0602	0.2555	0.8812	0.6070	0.4180
852	AQKFNGLTV	0.0614	0.2607	0.8737	0.5250	0.4180
587	ITPCSFGGV	0.0807	0.3425	0.4491	0.1620	0.4180
951	VVNQNAQAL	0.0663	0.2814	0.6005	0.9220	0.4176
1004	LQTYVTQQL	0.0565	0.2398	0.8331	1.0520	0.4174
464	FERDISTEI	0.0616	0.2613	0.9631	0.2080	0.4162
339	GEVFNATRF	0.0463	0.1967	0.7328	2.1930	0.4162
757	GSFCTQLNR	0.0594	0.2520	0.6217	1.4060	0.4156
714	IPTNFTISV	0.0614	0.2606	0.9763	0.1510	0.4146
1168	DISGINASV	0.0702	0.2979	0.7388	0.0930	0.4133
813	SKRSFIEDL	0.0597	0.2533	0.7459	0.9610	0.4132
954	QNAQALNTL	0.0630	0.2676	0.6442	0.9730	0.4129
544	NGLTGTGVL	0.0576	0.2446	0.9017	0.6380	0.4118
349	SVYAWNRKR	0.0621	0.2638	0.3125	2.0170	0.4115
1189	VAKNLNESL	0.0606	0.2575	0.7256	0.9000	0.4113
968	SNFGAISSV	0.0604	0.2564	0.9049	0.3810	0.4111
1148	FKEELDKYF	0.0567	0.2408	0.3314	2.3990	0.4105
295	PLSETKCTL	0.0582	0.2472	0.9680	0.3510	0.4100
516	ELLHAPATV	0.0612	0.2599	0.9762	0.0430	0.4085
1146	DSFKEELDK	0.0679	0.2881	0.6846	0.3490	0.4082
1032	CVLGQSKRV	0.0602	0.2556	0.9255	0.2630	0.4076
13	SQCVNLTTR	0.0613	0.2604	0.4194	1.6760	0.4071
378	KCYGVSPTK	0.0566	0.2402	0.8852	0.6740	0.4067
1133	VNNTVYDPL	0.0580	0.2461	0.8095	0.7790	0.4065
934	IQDSLSSTA	0.0786	0.3339	0.6537	-0.5120	0.4064
70	VSGTNGTKR	0.0686	0.2911	0.3014	1.4010	0.4064
1010	QQLIRAAEI	0.0609	0.2584	0.7689	0.6510	0.4063
707	YSNNSIAIP	0.0924	0.3925	0.0296	0.1850	0.4062
410	IAPGQTGKI	0.0626	0.2658	0.7509	0.5460	0.4057
1178	NIQKEIDRL	0.0683	0.2899	0.3929	1.1350	0.4056

144	YYHKNNKSW	0.0482	0.2048	0.9329	1.2060	0.4050
574	DAVRDPQTL	0.0524	0.2224	0.9547	0.7790	0.4046
1041	DFCGKGYHL	0.0512	0.2175	0.9593	0.8620	0.4045
195	KNIDGYFKI	0.0575	0.2443	0.9096	0.4530	0.4034
318	FRVQPTESI	0.0551	0.2341	0.8400	0.8650	0.4033
914	NVLYENQKL	0.0590	0.2504	0.6517	1.0990	0.4031
684	ARSVASQSI	0.0569	0.2418	0.7295	1.0130	0.4019
1044	GKGYHLMSF	0.0457	0.1942	0.6331	2.2350	0.4009
577	RDPQTLEIL	0.0517	0.2194	0.8952	0.9230	0.3998
311	GIYQTSNFR	0.0531	0.2254	0.5866	1.7220	0.3995
60	SNVTWFHAI	0.0605	0.2567	0.8106	0.4080	0.3987
1026	ATKMSECVL	0.0600	0.2546	0.5499	1.2290	0.3985
520	APATVCGPK	0.0592	0.2514	0.8784	0.2980	0.3980
151	SWMESEFRV	0.0607	0.2578	0.6743	0.7800	0.3980
787	QIYKTPPIK	0.0587	0.2493	0.6893	0.8940	0.3974
820	DLLFNKVTL	0.0545	0.2313	0.9345	0.5090	0.3969
254	SSSGWTAGA	0.0820	0.3482	0.5197	-0.5850	0.3969
966	LSSNFGAIS	0.1184	0.5028	0.0450	-2.2540	0.3968
685	RSVASQSII	0.0718	0.3047	0.2502	1.0510	0.3948
1210	IKWPWYIWL	0.0521	0.2213	0.7716	1.1470	0.3944
449	YNYLYRLFR	0.0661	0.2806	0.2092	1.6400	0.3940
310	KGIYQTSNF	0.0470	0.1995	0.5374	2.2770	0.3940
36	VYYPDKVFR	0.0438	0.1861	0.7288	1.9680	0.3938
387	LNDLCFTNV	0.0773	0.3283	0.4039	0.0910	0.3935
379	CYGVSPTKL	0.0499	0.2117	0.8371	1.1160	0.3930
265	YYVGYLQPR	0.0545	0.2313	0.4235	1.9610	0.3928
82	PVLPFNDGV	0.0610	0.2592	0.8458	0.1320	0.3927
495	YGFQPTNGV	0.0613	0.2601	0.8182	0.1940	0.3926
695	YTMSLGAEN	0.1049	0.4452	0.0275	-1.1380	0.3925
1220	FIAGLIAIV	0.0820	0.3483	0.1792	0.3350	0.3919
993	IDRLITGRL	0.0517	0.2194	0.9217	0.6700	0.3912
1195	ESLIDLQEL	0.0558	0.2368	0.7123	0.9500	0.3912
892	AALQIPFAM	0.0591	0.2511	0.7707	0.4890	0.3911
184	GNFKNLREF	0.0465	0.1975	0.5354	2.2660	0.3911
798	GGFNFSQIL	0.0534	0.2267	0.9224	0.5160	0.3909
925	NQFNSAIGK	0.0589	0.2500	0.6549	0.8430	0.3904
484	EGFNCYFPL	0.0528	0.2240	0.8914	0.6430	0.3899
1262	EPVLKGVKL	0.0504	0.2138	0.9768	0.5780	0.3892
1006	TYVTQQLIR	0.0539	0.2287	0.4812	1.7570	0.3887
1156	FKNHTSPDV	0.0590	0.2503	0.8781	0.1230	0.3882
821	LLFNKVTLA	0.0668	0.2835	0.8590	-0.5390	0.3854

235	ITRFQTLLA	0.0734	0.3116	0.5940	-0.3440	0.3835
639	GSNVFQTRA	0.0832	0.3532	0.4521	-0.7580	0.3831
362	VADYSVLYN	0.1056	0.4482	0.0270	-1.3840	0.3831
1094	VFVSNGTHW	0.0461	0.1957	0.8577	1.1620	0.3824
814	KRSFIEDLL	0.0540	0.2291	0.5818	1.3180	0.3823
1225	IAIVMVTIM	0.0631	0.2681	0.5943	0.4970	0.3820
890	AGAALQIPF	0.0578	0.2455	0.1086	2.3990	0.3817
706	AYSNNSIAI	0.0573	0.2433	0.5910	0.9860	0.3813
448	NYNYLYRLF	0.0481	0.2044	0.2741	2.7120	0.3811
861	LPPLLTDEM	0.0575	0.2442	0.8939	0.0360	0.3801
314	QTSNFRVQP	0.0867	0.3682	0.0601	0.0400	0.3792
402	IRGDEVRQI	0.0510	0.2164	0.8674	0.6480	0.3789
300	KCTLKSFTV	0.0605	0.2567	0.6747	0.4160	0.3787
395	VYADSFVIR	0.0499	0.2120	0.4541	1.9690	0.3786
259	TAGAAAYYV	0.0769	0.3263	0.3052	0.1270	0.3785
1208	QYIKWPWYI	0.0568	0.2411	0.6768	0.7140	0.3783
40	DKVFRSSVL	0.0475	0.2019	0.9661	0.6210	0.3778
186	FKNLREFVF	0.0544	0.2309	0.1337	2.5360	0.3778
616	NCTEVPVAI	0.0529	0.2248	0.8124	0.6030	0.3768
1034	LGQSKRVDF	0.0515	0.2189	0.2903	2.2870	0.3768
826	VTLADAGFI	0.0776	0.3294	0.0776	0.6940	0.3758
268	GYLQPRTFL	0.0437	0.1857	0.8981	1.1050	0.3757
1217	WLGFIAGLI	0.0794	0.3372	0.1312	0.3730	0.3755
467	DISTEIYQA	0.0656	0.2785	0.8429	-0.5950	0.3752
901	QMAYRFNGI	0.0748	0.3177	0.1414	0.7220	0.3750
856	NGLTVLPPL	0.0548	0.2327	0.7122	0.6380	0.3714
1011	QLIRAAEIR	0.0643	0.2729	0.1420	1.5370	0.3711
1206	YEQYIKWPW	0.0608	0.2581	0.4864	0.7980	0.3709
1042	FCGKGYHLM	0.0666	0.2827	0.6099	-0.0720	0.3706
56	LPFFSNVTW	0.0461	0.1958	0.9649	0.5910	0.3701
1024	LAATKMSEC	0.0836	0.3549	0.0253	0.2290	0.3701
1078	APAICHDGK	0.0536	0.2277	0.8363	0.3310	0.3697
851	CAQKFNGLT	0.0941	0.3995	0.0381	-0.7170	0.3694
298	ETKCTLKSF	0.0487	0.2070	0.2949	2.3610	0.3693
1003	SLQTYVTQQ	0.0798	0.3387	0.2389	-0.1140	0.3689
250	TPGDSSSGW	0.0483	0.2051	0.9333	0.4440	0.3673
354	NRKRISNCV	0.0503	0.2136	0.8539	0.5000	0.3667
54	LFLPFFSNV	0.0565	0.2398	0.7233	0.3650	0.3665
8	LPLVSSQCV	0.0517	0.2194	0.9525	0.0800	0.3663
97	KSNIIRGWI	0.0779	0.3308	0.0956	0.4210	0.3662
169	EYVSQPFLM	0.0484	0.2055	0.9567	0.3380	0.3659

643	FQTRAGCLI	0.0726	0.3081	0.2043	0.5260	0.3651
281	ENGTITDAV	0.0605	0.2567	0.7430	-0.0640	0.3650
1062	FLHVTYVPA	0.0643	0.2731	0.7894	-0.5340	0.3649
277	LKYNENGTI	0.0628	0.2665	0.3799	0.8230	0.3647
504	GYQPYRVVV	0.0479	0.2035	0.9353	0.4150	0.3645
766	ALTGIAVEQ	0.0660	0.2802	0.5096	0.1540	0.3643
135	FCNDPFLGV	0.0685	0.2910	0.4509	0.0970	0.3634
1247	CCSCGSCCK	0.0578	0.2454	0.6458	0.4100	0.3628
579	PQTLEILDI	0.0563	0.2392	0.8374	-0.0450	0.3625
1158	NHTSPDVDL	0.0500	0.2124	0.6432	1.0640	0.3620
601	GTNTSNQVA	0.0903	0.3836	0.1793	-0.9750	0.3618
774	QDKNTQEVF	0.0442	0.1876	0.4698	2.0710	0.3617
658	NSYECDIPI	0.0669	0.2838	0.2048	0.9400	0.3616
333	TNLCPFGEV	0.0613	0.2602	0.6142	0.1680	0.3607
168	FEYVSQPFL	0.0550	0.2336	0.5403	0.9210	0.3607
1161	SPDVDLGDI	0.0591	0.2509	0.6903	0.1170	0.3603
61	NVTWFHAIH	0.0746	0.3169	0.4024	-0.3420	0.3602
1007	YVTQQLIRA	0.0764	0.3243	0.3867	-0.4430	0.3602
342	FNATRFASV	0.0708	0.3008	0.3491	0.1310	0.3597
92	FASTEKSNI	0.0777	0.3300	0.0517	0.4360	0.3596
182	KQGNFKNLR	0.0615	0.2611	0.1642	1.4740	0.3594
944	ALGKLQDVV	0.0684	0.2906	0.3333	0.3690	0.3590
780	EVFAQVKQI	0.0526	0.2233	0.7272	0.5320	0.3589
614	DVNCTEVPV	0.0586	0.2487	0.7354	-0.0060	0.3588
338	FGEVFNATR	0.0664	0.2820	0.2306	0.8340	0.3583
1029	MSECVLGQS	0.1088	0.4621	0.0417	-2.2010	0.3583
450	NYLYRLFRK	0.0491	0.2083	0.7517	0.7320	0.3576
511	VVLSFELLH	0.0781	0.3316	0.2611	-0.2700	0.3573
208	TPINLVRDL	0.0545	0.2314	0.6102	0.6750	0.3566
512	VLSFELLHA	0.0703	0.2984	0.5214	-0.4110	0.3561
1151	ELDKYFKNH	0.0802	0.3405	0.4566	-1.0630	0.3559
1075	FTTAPAICH	0.0869	0.3690	0.0952	-0.5470	0.3559
679	NSPRRARSV	0.0641	0.2721	0.4289	0.3870	0.3558
526	GPKKSTNLV	0.0527	0.2235	0.9405	-0.1780	0.3557
1260	DSEPVLKGV	0.0645	0.2741	0.6237	-0.2440	0.3554
112	SKTQSLLIV	0.0678	0.2880	0.3660	0.2420	0.3550
782	FAQVKQIYK	0.0706	0.2998	0.2220	0.4370	0.3549
475	AGSTPCNGV	0.0565	0.2399	0.7193	0.1390	0.3547
744	GDSTECSNL	0.0495	0.2103	0.8057	0.4570	0.3540
1171	GINASVVNI	0.0587	0.2494	0.5491	0.4440	0.3540
1083	HDGKAHFPR	0.0602	0.2555	0.3516	0.9160	0.3540

198	DGYFKIYSK	0.0505	0.2145	0.8981	0.0780	0.3531
243	ALHRSYLTP	0.0693	0.2942	0.2766	0.3460	0.3530
118	LIVNNATNV	0.0664	0.2821	0.2805	0.5730	0.3528
969	NFGAISSVL	0.0534	0.2266	0.4907	1.0500	0.3527
686	SVASQSIIA	0.0796	0.3379	0.1639	-0.1950	0.3527
1073	KNFTTAPAI	0.0536	0.2276	0.6894	0.4340	0.3527
139	PFLGVYYHK	0.0486	0.2062	0.9634	0.0310	0.3523
48	LHSTQDLFL	0.0625	0.2656	0.2398	1.0090	0.3520
166	CTFEYVSQP	0.0732	0.3106	0.1732	0.2950	0.3513
320	VQPTESIVR	0.0582	0.2471	0.1142	1.7390	0.3512
1106	QRNFYEPQI	0.0504	0.2140	0.6906	0.6700	0.3511
41	KVFRSSVLH	0.0613	0.2601	0.7118	-0.3170	0.3510
306	FTVEKGIYQ	0.0821	0.3487	0.0415	-0.0900	0.3505
979	DILSRLDKV	0.0633	0.2687	0.4475	0.2860	0.3501
1229	MVTIMLCCM	0.0664	0.2818	0.2884	0.5010	0.3501
1052	FPQSAPHGV	0.0572	0.2429	0.7286	-0.0630	0.3490
110	LDSKTQSLL	0.0604	0.2566	0.3961	0.6570	0.3489
1057	PHGVVFLHV	0.0537	0.2281	0.8496	-0.1670	0.3472
1257	DEDDSEPVL	0.0456	0.1934	0.9506	0.2220	0.3471
1028	KMSECVLGQ	0.0783	0.3323	0.0522	0.1290	0.3466
806	LPDPSKPSK	0.0483	0.2050	0.9443	-0.0030	0.3465
938	LSSTASALG	0.0952	0.4044	0.0456	-1.3010	0.3462
1071	QEKNFTTAP	0.0549	0.2330	0.7075	0.1340	0.3458
850	ICAQKFNGL	0.0558	0.2369	0.3692	1.0630	0.3454
943	SALGKLQDV	0.0630	0.2675	0.3167	0.5920	0.3446
225	PLVDLPIGI	0.0528	0.2240	0.8079	-0.0150	0.3445
948	LQDVVNQNA	0.0814	0.3455	0.1672	-0.5310	0.3440
922	LIANQFNSA	0.0720	0.3056	0.3495	-0.2850	0.3438
904	YRFNGIGVT	0.0656	0.2786	0.5184	-0.2530	0.3437
58	FFSNVTWFH	0.0823	0.3495	0.1609	-0.6120	0.3431
150	KSWMESEFR	0.0553	0.2349	0.0657	1.9450	0.3420
1050	MSFPQSAPH	0.0768	0.3260	0.2006	-0.2880	0.3417
797	FGGFNFSQI	0.0544	0.2309	0.7783	-0.1290	0.3412
888	FGAGAALQI	0.0730	0.3099	0.1370	0.2000	0.3405
1025	AATKMSECV	0.0646	0.2742	0.2634	0.5230	0.3399
997	ITGRLQSLQ	0.0814	0.3455	0.0291	-0.2190	0.3389
728	PVSMTKTSV	0.0586	0.2490	0.5910	0.0170	0.3385
1154	KYFKNHTSP	0.0479	0.2032	0.7514	0.4490	0.3384
525	CGPKKSTNL	0.0559	0.2375	0.4442	0.6790	0.3381
155	SEFRVYSSA	0.0571	0.2424	0.8593	-0.6730	0.3377
18	LTTRTQLPP	0.0760	0.3227	0.0343	0.1920	0.3375

1030	SECVLGQSK	0.0597	0.2533	0.4194	0.4270	0.3375
662	CDIPIGAGI	0.0529	0.2246	0.7201	0.0880	0.3370
530	STNLVKNKC	0.0776	0.3295	0.0281	0.0530	0.3363
677	QTNSPRRAR	0.0551	0.2338	0.2172	1.3920	0.3360
1121	FVSGNCDVV	0.0715	0.3035	0.0945	0.3640	0.3359
189	LREFVFKNI	0.0492	0.2087	0.5983	0.7420	0.3356
1216	IWLGFIAGL	0.0541	0.2296	0.3027	1.1900	0.3345
1122	VSGNCDVVI	0.0709	0.3012	0.0896	0.3690	0.3331
760	CTQLNRALT	0.0881	0.3742	0.0273	-0.9050	0.3330
1159	HTSPDVDLG	0.0921	0.3910	0.0692	-1.3710	0.3328
809	PSKPSKRSF	0.0415	0.1760	0.3885	1.9540	0.3320
741	YICGDSTEC	0.0746	0.3167	0.0459	0.1680	0.3320
304	KSFTVEKGI	0.0559	0.2372	0.3492	0.8420	0.3316
529	KSTNLVKNK	0.0576	0.2444	0.3464	0.6770	0.3302
1190	AKNLNESLI	0.0559	0.2373	0.3904	0.6810	0.3299
1104	VTQRNFYEP	0.0763	0.3241	0.0356	0.0080	0.3298
642	VFQTRAGCL	0.0552	0.2345	0.2033	1.2930	0.3297
637	STGSNVFQT	0.0849	0.3604	0.0900	-0.8890	0.3294
223	LEPLVDLPI	0.0602	0.2557	0.3083	0.5420	0.3290
428	DFTGCVIAW	0.0454	0.1926	0.7136	0.5610	0.3277
96	EKSNIIRGW	0.0487	0.2069	0.5544	0.7360	0.3269
16	VNLTTRTQL	0.0561	0.2381	0.2980	0.8750	0.3266
218	QGFSALEPL	0.0539	0.2290	0.4222	0.6680	0.3257
33	TRGVYYPDK	0.0581	0.2467	0.3908	0.3820	0.3245
132	EFQFCNDPF	0.0448	0.1901	0.0759	2.4590	0.3244
330	PNITNLCPF	0.0480	0.2040	0.1803	1.8660	0.3243
49	HSTQDLFLP	0.0746	0.3165	0.0279	0.0660	0.3240
836	QYGDCLGDI	0.0542	0.2300	0.3687	0.7500	0.3228
803	SQILPDPSK	0.0561	0.2381	0.3755	0.5570	0.3223
416	GKIADYNYK	0.0515	0.2187	0.5496	0.4140	0.3218
1228	VMVTIMLCC	0.0711	0.3019	0.0364	0.2850	0.3216
350	VYAWNRKRI	0.0492	0.2091	0.4390	0.9320	0.3215
989	AEVQIDRLI	0.0555	0.2357	0.4169	0.4540	0.3210
841	LGDIAARDL	0.0555	0.2357	0.3981	0.5090	0.3209
1056	APHGVVFLH	0.0601	0.2552	0.6418	-0.6120	0.3209
1088	HFPREGVFV	0.0602	0.2555	0.2574	0.5300	0.3206
648	GCLIGAEHV	0.0526	0.2234	0.5365	0.3280	0.3203
621	PVAIHADQL	0.0598	0.2540	0.2160	0.6770	0.3203
1014	RAAEIRASA	0.0613	0.2602	0.4718	-0.2130	0.3203
1141	LQPELDSFK	0.0616	0.2614	0.2626	0.3840	0.3200
359	SNCVADYSV	0.0622	0.2642	0.2911	0.2330	0.3195

1222	AGLIAIVMV	0.0601	0.2550	0.3551	0.2190	0.3192
833	FIKQYGDCL	0.0602	0.2555	0.1025	0.9560	0.3187
1204	GKYEQYIKW	0.0469	0.1989	0.4625	0.9900	0.3178
255	SSGWTAGAA	0.0801	0.3401	0.0320	-0.5510	0.3174
600	PGTNTSNQV	0.0515	0.2186	0.8111	-0.4580	0.3173
1037	SKRVDFCGK	0.0515	0.2187	0.4622	0.5670	0.3163
161	SSANNCTFE	0.0911	0.3868	0.0289	-1.5070	0.3158
1120	TFVSGNCDV	0.0594	0.2520	0.1970	0.6800	0.3156
1053	PQSAPHGVV	0.0547	0.2322	0.6207	-0.1990	0.3154
1182	EIDRLNEVA	0.0726	0.3084	0.2718	-0.6860	0.3149
227	VDLPIGINI	0.0530	0.2249	0.4750	0.3550	0.3139
546	LTGTGVLTE	0.0929	0.3945	0.0472	-1.7760	0.3128
907	NGIGVTQNV	0.0567	0.2406	0.4641	0.0490	0.3127
365	YSVLYNSAS	0.0994	0.4220	0.0318	-2.2860	0.3125
871	AQYTSALLA	0.0721	0.3061	0.0779	-0.1200	0.3118
936	DSLSSTASA	0.0583	0.2475	0.7081	-0.8420	0.3116
1109	FYEPQIITT	0.0586	0.2490	0.5961	-0.5390	0.3115
731	MTKTSVDCT	0.0798	0.3390	0.0244	-0.6280	0.3113
34	RGVYYPDKV	0.0539	0.2289	0.4171	0.3800	0.3104
1177	VNIQKEIDR	0.0536	0.2275	0.0468	1.5100	0.3100
399	SFVIRGDEV	0.0582	0.2472	0.2575	0.4740	0.3095
1227	IVMVTIMLC	0.0665	0.2822	0.0504	0.3950	0.3095
1149	KEELDKYFK	0.0494	0.2097	0.6000	0.1870	0.3090
688	ASQSIIAYT	0.0768	0.3261	0.0533	-0.5060	0.3088
868	EMIAQYTSA	0.0628	0.2667	0.5082	-0.6880	0.3085
460	NLKPFERDI	0.0505	0.2143	0.4125	0.6430	0.3083
502	GVGYQPYRV	0.0592	0.2511	0.2931	0.2520	0.3077
121	NNATNVVIK	0.0612	0.2598	0.1813	0.4130	0.3076
1070	AQEKNFTTA	0.0665	0.2823	0.3680	-0.6050	0.3073
360	NCVADYSVL	0.0506	0.2150	0.2334	1.1440	0.3072
913	QNVLYENQK	0.0552	0.2344	0.3460	0.4130	0.3070
163	ANNCTFEYV	0.0590	0.2504	0.2859	0.2660	0.3066
618	TEVPVAIHA	0.0557	0.2364	0.6744	-0.6210	0.3065
730	SMTKTSVDC	0.0684	0.2905	0.0318	0.2000	0.3053
371	SASFSTFKC	0.0654	0.2778	0.0974	0.2570	0.3053
1086	KAHFPREGV	0.0561	0.2383	0.2886	0.4740	0.3053
120	VNNATNVVI	0.0572	0.2429	0.2952	0.3590	0.3052
545	GLTGTGVLT	0.0602	0.2556	0.5832	-0.7600	0.3051
76	TKRFDNPVL	0.0449	0.1907	0.4284	1.0020	0.3050
214	RDLPQGFSA	0.0503	0.2135	0.7862	-0.5340	0.3048
581	TLEILDITP	0.0700	0.2974	0.0758	-0.0900	0.3043

1261	SEPVLKGVK	0.0563	0.2390	0.3575	0.2280	0.3041
1202	ELGKYEQYI	0.0630	0.2676	0.1956	0.1380	0.3038
222	ALEPLVDLP	0.0686	0.2913	0.0237	0.1780	0.3037
55	FLPFFSNVT	0.0665	0.2825	0.3889	-0.7480	0.3034
759	FCTQLNRAL	0.0578	0.2454	0.0807	0.8980	0.3024
181	GKQGNFKNL	0.0444	0.1886	0.5457	0.6260	0.3017
1031	ECVLGQSKR	0.0516	0.2190	0.0729	1.4330	0.3015
133	FQFCNDPFL	0.0557	0.2364	0.1396	0.8800	0.3013
105	IFGTTLDSK	0.0564	0.2394	0.2656	0.4360	0.3010
626	ADQLTPTWR	0.0502	0.2131	0.1625	1.2660	0.3008
17	NLTTRTQLP	0.0670	0.2845	0.0426	0.1720	0.2994
623	AIHADQLTP	0.0639	0.2715	0.0338	0.4540	0.2993
1233	MLCCMTSCC	0.0683	0.2898	0.0300	0.0990	0.2993
219	GFSALEPLV	0.0539	0.2289	0.3858	0.2500	0.2992
660	YECDIPIGA	0.0684	0.2904	0.2651	-0.6280	0.2988
145	YHKNNKSWM	0.0532	0.2259	0.4032	0.2380	0.2982
3	VFLVLLPLV	0.0559	0.2372	0.2534	0.4570	0.2981
1002	QSLQTYVTQ	0.0662	0.2809	0.0921	0.0140	0.2954
693	IAYTMSLGA	0.0662	0.2812	0.1772	-0.2640	0.2946
752	LLLQYGSFC	0.0661	0.2806	0.0255	0.1970	0.2942
720	ISVTTEILP	0.0654	0.2777	0.0244	0.2560	0.2941
501	NGVGYQPYR	0.0510	0.2166	0.0690	1.3430	0.2941
6	VLLPLVSSQ	0.0577	0.2451	0.3101	0.0420	0.2937
325	SIVRFPNIT	0.0622	0.2641	0.4000	-0.6130	0.2935
664	IPIGAGICA	0.0522	0.2217	0.7732	-0.8870	0.2933
503	VGYQPYRVV	0.0497	0.2111	0.4547	0.2670	0.2926
170	YVSQPFLMD	0.0851	0.3611	0.1604	-1.8680	0.2918
858	LTVLPPLLT	0.0754	0.3202	0.0335	-0.6750	0.2915
142	GVYYHKNNK	0.0535	0.2274	0.2068	0.6590	0.2913
77	KRFDNPVLP	0.0522	0.2215	0.2842	0.5400	0.2912
158	RVYSSANNC	0.0613	0.2601	0.0403	0.4790	0.2901
957	QALNTLVKQ	0.0597	0.2533	0.1959	0.1320	0.2893
1048	HLMSFPQSA	0.0723	0.3071	0.0778	-0.5940	0.2891
864	LLTDEMIAQ	0.0686	0.2914	0.0717	-0.2630	0.2890
462	KPFERDIST	0.0518	0.2199	0.7079	-0.7430	0.2889
754	LQYGSFCTQ	0.0553	0.2346	0.2915	0.2090	0.2888
101	IRGWIFGTT	0.0611	0.2595	0.4046	-0.6300	0.2887
810	SKPSKRSFI	0.0596	0.2531	0.0772	0.4770	0.2886
307	TVEKGIYQT	0.0693	0.2944	0.2393	-0.8370	0.2884
543	FNGLTGTGV	0.0608	0.2580	0.1869	0.0450	0.2883
519	HAPATVCGP	0.0655	0.2779	0.0323	0.0990	0.2877

903	AYRFNGIGV	0.0479	0.2033	0.2890	0.8080	0.2871
1124	GNCDVVIGI	0.0561	0.2384	0.2334	0.2660	0.2867
1256	FDEDDSEPV	0.0592	0.2512	0.2746	-0.1310	0.2858
263	AAYYVGYLQ	0.0610	0.2592	0.0319	0.4320	0.2856
471	EIYQAGSTP	0.0596	0.2530	0.1387	0.2260	0.2851
1232	IMLCCMTSC	0.0624	0.2651	0.0702	0.1870	0.2850
187	KNLREFVFK	0.0550	0.2335	0.2318	0.3320	0.2848
839	DCLGDIAAR	0.0504	0.2141	0.0770	1.1770	0.2845
175	FLMDLEGKQ	0.0687	0.2918	0.0385	-0.2610	0.2845
796	DFGGFNFSQ	0.0506	0.2149	0.5678	-0.3160	0.2843
521	PATVCGPKK	0.0524	0.2226	0.3579	0.1550	0.2840
324	ESIVRFPNI	0.0559	0.2374	0.1605	0.4500	0.2839
179	LEGKQGNFK	0.0597	0.2533	0.1923	0.0310	0.2837
711	SIAIPTNFT	0.0697	0.2958	0.0342	-0.3660	0.2826
280	NENGTITDA	0.0541	0.2297	0.6125	-0.7800	0.2826
436	WNSNNLDSK	0.0587	0.2491	0.0771	0.4350	0.2824
985	DKVEAEVQI	0.0522	0.2215	0.2716	0.3940	0.2819
602	TNTSNQVAV	0.0588	0.2497	0.1879	0.0750	0.2817
1221	IAGLIAIVM	0.0614	0.2608	0.1024	0.1120	0.2817
1258	EDDSEPVLK	0.0596	0.2532	0.3103	-0.3620	0.2816
364	DYSVLYNSA	0.0476	0.2022	0.6742	-0.4370	0.2815
950	DVVNQNAQA	0.0581	0.2468	0.4587	-0.6840	0.2814
344	ATRFASVYA	0.0583	0.2477	0.2996	-0.2230	0.2814
418	IADYNYKLP	0.0640	0.2717	0.0235	0.1050	0.2805
138	DPFLGVYYH	0.0464	0.1971	0.8558	-0.9030	0.2803
807	PDPSKPSKR	0.0457	0.1942	0.3295	0.7300	0.2802
513	LSFELLHAP	0.0599	0.2543	0.0704	0.3080	0.2802
100	IIRGWIFGT	0.0566	0.2402	0.3840	-0.3640	0.2796
567	RDIADTTDA	0.0540	0.2293	0.5196	-0.5600	0.2792
514	SFELLHAPA	0.0518	0.2200	0.5317	-0.4110	0.2792
705	VAYSNNSIA	0.0640	0.2715	0.1060	-0.1800	0.2784
673	SYQTQTNSP	0.0527	0.2238	0.1808	0.5440	0.2781
837	YGDCLGDIA	0.0756	0.3208	0.0632	-1.0470	0.2779
893	ALQIPFAMQ	0.0551	0.2341	0.2289	0.1800	0.2774
455	LFRKSNLKP	0.0551	0.2339	0.1349	0.4570	0.2770
264	AYYVGYLQP	0.0507	0.2151	0.1273	0.8540	0.2769
786	KQIYKTPPI	0.0517	0.2194	0.1009	0.8330	0.2762
843	DIAARDLIC	0.0664	0.2817	0.0232	-0.1860	0.2759
897	PFAMQMAYR	0.0497	0.2111	0.0295	1.1980	0.2754
527	PKKSTNLVK	0.0498	0.2116	0.4238	0.0020	0.2752
1132	IVNNTVYDP	0.0612	0.2600	0.0540	0.1420	0.2752

895	QIPFAMQMA	0.0644	0.2733	0.1297	-0.3610	0.2747
1018	IRASANLAA	0.0660	0.2804	0.1131	-0.4560	0.2746
978	NDILSRLDK	0.0569	0.2418	0.1133	0.3110	0.2743
39	PDKVFRSSV	0.0459	0.1947	0.7065	-0.5270	0.2743
123	ATNVVIKVC	0.0602	0.2557	0.0620	0.1840	0.2742
768	TGIAVEQDK	0.0572	0.2430	0.1095	0.2940	0.2741
328	RFPNITNLC	0.0576	0.2445	0.0435	0.4580	0.2739
1049	LMSFPQSAP	0.0606	0.2572	0.0435	0.2030	0.2738
549	TGVLTESNK	0.0518	0.2200	0.2805	0.2250	0.2733
1219	GFIAGLIAI	0.0537	0.2281	0.0861	0.6400	0.2730
698	SLGAENSVA	0.0669	0.2840	0.1225	-0.5900	0.2729
1230	VTIMLCCMT	0.0691	0.2935	0.0494	-0.5600	0.2729
291	CALDPLSET	0.0596	0.2529	0.4035	-0.8150	0.2727
194	FKNIDGYFK	0.0548	0.2328	0.1407	0.3720	0.2725
589	PCSFGGVSV	0.0504	0.2138	0.4363	-0.1390	0.2723
126	VVIKVCEFQ	0.0593	0.2519	0.0323	0.3050	0.2720
884	SGWTFGAGA	0.0590	0.2503	0.3271	-0.5670	0.2711
656	VNNSYECDI	0.0581	0.2467	0.0512	0.3270	0.2708
115	QSLLIVNNA	0.0673	0.2855	0.0525	-0.4540	0.2707
1	MFVFLVLLP	0.0578	0.2456	0.0342	0.3950	0.2704
645	TRAGCLIGA	0.0594	0.2524	0.2456	-0.3820	0.2701
7	LLPLVSSQC	0.0612	0.2600	0.0419	0.0700	0.2698
747	TECSNLLLQ	0.0645	0.2739	0.0382	-0.1990	0.2696
680	SPRRARSVA	0.0500	0.2124	0.5717	-0.5970	0.2683
283	GTITDAVDC	0.0606	0.2572	0.0655	0.0240	0.2683
1164	VDLGDISGI	0.0512	0.2173	0.2843	0.1680	0.2683
1135	NTVYDPLQP	0.0571	0.2424	0.0524	0.3390	0.2672
941	TASALGKLQ	0.0623	0.2647	0.0241	-0.0240	0.2671
446	GGNYNYLYR	0.0490	0.2081	0.0571	0.9920	0.2663
911	VTQNVLYEN	0.0771	0.3275	0.0295	-1.3370	0.2651
804	QILPDPSKP	0.0533	0.2264	0.1470	0.3180	0.2643
862	PPLLTDEMI	0.0480	0.2037	0.4627	-0.1840	0.2639
31	SFTRGVYYP	0.0528	0.2243	0.1127	0.4300	0.2627
287	DAVDCALDP	0.0617	0.2620	0.0264	-0.0710	0.2624
726	ILPVSMTKT	0.0627	0.2664	0.2498	-0.8450	0.2616
256	SGWTAGAAA	0.0580	0.2462	0.2771	-0.5230	0.2616
716	TNFTISVTT	0.0573	0.2434	0.4195	-0.9120	0.2608
237	RFQTLLALH	0.0588	0.2499	0.1182	-0.1460	0.2603
393	TNVYADSFV	0.0581	0.2468	0.0507	0.1140	0.2601
1093	GVFVSNGTH	0.0641	0.2720	0.1313	-0.6310	0.2601
1008	VTQQLIRAA	0.0655	0.2780	0.0669	-0.5790	0.2591

580	QTLEILDIT	0.0693	0.2943	0.0407	-0.8410	0.2583
1183	IDRLNEVAK	0.0476	0.2023	0.3485	0.0620	0.2576
609	AVLYQDVNC	0.0553	0.2347	0.0266	0.3620	0.2568
669	GICASYQTQ	0.0576	0.2446	0.0767	0.0070	0.2564
322	PTESIVRFP	0.0664	0.2818	0.0248	-0.5900	0.2560
424	KLPDDFTGC	0.0548	0.2329	0.1287	0.0740	0.2559
629	LTPTWRVYS	0.0860	0.3652	0.0460	-2.3270	0.2558
440	NLDSKVGGN	0.0746	0.3169	0.0239	-1.2940	0.2557
842	GDIAARDLI	0.0551	0.2339	0.0887	0.1650	0.2555
116	SLLIVNNAT	0.0664	0.2821	0.0431	-0.6640	0.2553
43	FRSSVLHST	0.0624	0.2650	0.1128	-0.5510	0.2544
1076	TTAPAICHD	0.0801	0.3400	0.0767	-1.9460	0.2542
854	KFNGLTVLP	0.0545	0.2313	0.0729	0.2360	0.2541
641	NVFQTRAGC	0.0547	0.2320	0.0270	0.3560	0.2539
539	VNFNFNGLT	0.0659	0.2797	0.0583	-0.6910	0.2539
266	YVGYLQPRT	0.0681	0.2891	0.0280	-0.8020	0.2532
414	QTGKIADYN	0.0764	0.3245	0.0279	-1.5170	0.2529
1019	RASANLAAT	0.0627	0.2662	0.0393	-0.4040	0.2519
902	MAYRFNGIG	0.0718	0.3050	0.0308	-1.1580	0.2518
947	KLQDVVNQN	0.0602	0.2557	0.3906	-1.2510	0.2517
771	AVEQDKNTQ	0.0571	0.2426	0.0451	0.0420	0.2515
477	STPCNGVEG	0.0725	0.3080	0.0926	-1.4090	0.2514
682	RRARSVASQ	0.0548	0.2329	0.0500	0.2170	0.2512
845	AARDLICAQ	0.0543	0.2304	0.0871	0.1490	0.2509
389	DLCFTNVYA	0.0595	0.2526	0.2154	-0.6820	0.2508
553	TESNKKFLP	0.0579	0.2459	0.0343	-0.0080	0.2507
1235	CCMTSCCSC	0.0568	0.2414	0.0251	0.1070	0.2505
974	SSVLNDILS	0.0815	0.3460	0.0515	-2.0650	0.2504
758	SFCTQLNRA	0.0609	0.2587	0.0549	-0.3360	0.2502
1107	RNFYEPQII	0.0477	0.2026	0.0847	0.6890	0.2498
605	SNQVAVLYQ	0.0599	0.2544	0.0417	-0.2270	0.2493
690	QSIIAYTMS	0.0812	0.3449	0.0521	-2.0690	0.2492
531	TNLVKNKCV	0.0508	0.2155	0.1532	0.2130	0.2491
228	DLPIGINIT	0.0576	0.2446	0.3535	-0.9700	0.2491
739	TMYICGDST	0.0622	0.2640	0.0421	-0.4250	0.2490
383	SPTKLNDLC	0.0604	0.2563	0.0265	-0.2260	0.2490
1193	LNESLIDLQ	0.0606	0.2575	0.0289	-0.2570	0.2490
185	NFKNLREFV	0.0499	0.2118	0.0802	0.4870	0.2482
855	FNGLTVLPP	0.0597	0.2536	0.0290	-0.2050	0.2477
877	LLAGTITSG	0.0639	0.2712	0.2412	-1.2010	0.2474
784	QVKQIYKTP	0.0517	0.2194	0.0480	0.4080	0.2470

561	PFQQFGRDI	0.0472	0.2003	0.2316	0.2380	0.2469
596	SVITPGTNT	0.0617	0.2618	0.0306	-0.3980	0.2465
517	LLHAPATVC	0.0574	0.2436	0.0497	-0.0990	0.2461
1017	EIRASANLA	0.0584	0.2482	0.1217	-0.4080	0.2460
313	YQTSNFRVQ	0.0578	0.2455	0.0259	-0.0770	0.2455
1242	SCLKGCCSC	0.0553	0.2347	0.0390	0.0990	0.2455
964	KQLSSNFGA	0.0586	0.2490	0.0691	-0.2880	0.2450
491	PLQSYGFQP	0.0613	0.2604	0.0584	-0.4930	0.2445
375	STFKCYGVS	0.0825	0.3501	0.0306	-2.2070	0.2444
220	FSALEPLVD	0.0789	0.3349	0.0226	-1.8830	0.2441
1061	VFLHVTYVP	0.0476	0.2023	0.1425	0.3990	0.2436
905	RFNGIGVTQ	0.0516	0.2193	0.1066	0.1600	0.2433
875	SALLAGTIT	0.0616	0.2617	0.0662	-0.5720	0.2431
21	RTQLPPAYT	0.0610	0.2590	0.0621	-0.5050	0.2431
832	GFIKQYGDC	0.0537	0.2282	0.0232	0.2260	0.2430
279	YNENGTITD	0.0733	0.3113	0.2215	-2.0310	0.2429
592	FGGVSVITP	0.0605	0.2568	0.0277	-0.3660	0.2426
1012	LIRAAEIRA	0.0577	0.2450	0.1172	-0.4030	0.2425
654	EHVNNSYEC	0.0512	0.2172	0.1485	0.0500	0.2420
15	CVNLTTRTQ	0.0573	0.2435	0.0346	-0.1510	0.2411
131	CEFQFCNDP	0.0545	0.2314	0.0285	0.0870	0.2401
953	NQNAQALNT	0.0631	0.2678	0.0278	-0.6400	0.2400
226	LVDLPIGIN	0.0728	0.3091	0.0370	-1.4940	0.2400
1255	KFDEDDSEP	0.0529	0.2247	0.0368	0.1960	0.2400
1239	SCCSCLKGC	0.0544	0.2311	0.0240	0.1070	0.2400
429	FTGCVIAWN	0.0755	0.3205	0.0402	-1.7340	0.2399
14	QCVNLTTRT	0.0583	0.2474	0.1297	-0.5400	0.2399
1035	GQSKRVDFC	0.0562	0.2388	0.0227	-0.0450	0.2399
933	KIQDSLSST	0.0573	0.2433	0.1526	-0.5320	0.2396
19	TTRTQLPPA	0.0596	0.2532	0.0691	-0.4810	0.2395
571	DTTDAVRDP	0.0558	0.2369	0.0487	-0.1150	0.2385
315	TSNFRVQPT	0.0646	0.2744	0.0358	-0.8290	0.2383
368	LYNSASFST	0.0522	0.2217	0.2697	-0.4840	0.2380
1074	NFTTAPAIC	0.0516	0.2191	0.0298	0.2840	0.2378
1128	VVIGIVNNT	0.0582	0.2472	0.1261	-0.5750	0.2374
242	LALHRSYLT	0.0593	0.2516	0.0981	-0.5830	0.2372
891	GAALQIPFA	0.0604	0.2566	0.0334	-0.4910	0.2370
670	ICASYQTQT	0.0616	0.2615	0.0613	-0.6750	0.2370
284	TITDAVDCA	0.0572	0.2429	0.1243	-0.4940	0.2368
1240	CCSCLKGCC	0.0543	0.2307	0.0234	0.0440	0.2364
1213	PWYIWLGFI	0.0484	0.2057	0.0601	0.4320	0.2363

663	DIPIGAGIC	0.0566	0.2405	0.0332	-0.1950	0.2358
582	LEILDITPC	0.0536	0.2278	0.0662	-0.0380	0.2358
753	LLQYGSFCT	0.0583	0.2473	0.1443	-0.6660	0.2357
201	FKIYSKHTP	0.0538	0.2283	0.0238	0.0760	0.2357
981	LSRLDKVEA	0.0531	0.2253	0.2131	-0.4330	0.2356
493	QSYGFQPTN	0.0630	0.2675	0.1289	-1.0270	0.2355
916	LYENQKLIA	0.0566	0.2402	0.0890	-0.3640	0.2353
1172	INASVVNIQ	0.0572	0.2428	0.0313	-0.2480	0.2351
113	KTQSLLIVN	0.0684	0.2903	0.0252	-1.2080	0.2337
1115	ITTDNTFVS	0.0769	0.3264	0.1382	-2.2710	0.2336
960	NTLVKQLSS	0.0781	0.3314	0.0681	-2.1640	0.2334
239	QTLLALHRS	0.0751	0.3190	0.2090	-2.3410	0.2333
647	AGCLIGAEH	0.0597	0.2536	0.0365	-0.5170	0.2332
427	DDFTGCVIA	0.0493	0.2091	0.5569	-1.1920	0.2331
376	TFKCYGVSP	0.0502	0.2131	0.0296	0.3030	0.2327
1200	LQELGKYEQ	0.0563	0.2389	0.0617	-0.3090	0.2327
1082	CHDGKAHFP	0.0550	0.2336	0.0252	-0.0960	0.2326
66	HAIHVSGTN	0.0678	0.2880	0.0933	-1.3900	0.2325
912	TQNVLYENQ	0.0550	0.2333	0.0251	-0.0980	0.2322
1263	PVLKGVKLH	0.0531	0.2255	0.3865	-1.0260	0.2322
783	AQVKQIYKT	0.0579	0.2457	0.0772	-0.5130	0.2317
883	TSGWTFGAG	0.0699	0.2970	0.0714	-1.5300	0.2312
1045	KGYHLMSFP	0.0520	0.2206	0.0367	0.1020	0.2312
336	CPFGEVFNA	0.0509	0.2161	0.3385	-0.7210	0.2309
622	VAIHADQLT	0.0613	0.2604	0.0237	-0.6670	0.2306
799	GFNFSQILP	0.0504	0.2138	0.1213	-0.0310	0.2305
620	VPVAIHADQ	0.0511	0.2168	0.2370	-0.4370	0.2305
248	YLTPGDSSS	0.0725	0.3078	0.2275	-2.2280	0.2305
1105	TQRNFYEPQ	0.0525	0.2231	0.0315	0.0450	0.2300
889	GAGAALQIP	0.0567	0.2407	0.0246	-0.2900	0.2299
801	NFSQILPDP	0.0505	0.2143	0.0355	0.2000	0.2296
1211	KWPWYIWLG	0.0631	0.2680	0.0916	-1.0480	0.2294
887	TFGAGAALQ	0.0552	0.2345	0.0233	-0.1780	0.2291
1063	LHVTYVPAQ	0.0512	0.2172	0.1192	-0.1210	0.2291
1038	KRVDFCGKG	0.0551	0.2338	0.2807	-0.9500	0.2284
630	TPTWRVYST	0.0527	0.2239	0.3649	-1.0190	0.2276
1079	PAICHDGKA	0.0570	0.2419	0.1515	-0.7400	0.2276
692	IIAYTMSLG	0.0663	0.2816	0.0292	-1.1730	0.2274
785	VKQIYKTPP	0.0509	0.2163	0.0268	0.1350	0.2270
128	IKVCEFQFC	0.0501	0.2127	0.0242	0.2120	0.2270
671	CASYQTQTN	0.0672	0.2855	0.0415	-1.2990	0.2268

1238	TSCCSCLKG	0.0664	0.2818	0.0770	-1.3310	0.2268
1001	LQSLQTYVT	0.0566	0.2402	0.0760	-0.4980	0.2267
1046	GYHLMSFPQ	0.0463	0.1966	0.1604	0.1110	0.2262
329	FPNITNLCP	0.0547	0.2321	0.0453	-0.2570	0.2260
1097	SNGTHWFVT	0.0626	0.2660	0.0390	-0.9210	0.2258
844	IAARDLICA	0.0565	0.2401	0.0966	-0.5820	0.2255
610	VLYQDVNCT	0.0537	0.2279	0.0779	-0.2830	0.2254
353	WNRKRISNC	0.0517	0.2195	0.0233	0.0380	0.2249
1215	YIWLGFIAG	0.0633	0.2689	0.0929	-1.1710	0.2243
68	IHVSGTNGT	0.0493	0.2093	0.2442	-0.4430	0.2238
217	PQGFSALEP	0.0558	0.2369	0.0494	-0.4150	0.2236
1245	KGCCSCGSC	0.0527	0.2238	0.0271	-0.0880	0.2235
401	VIRGDEVRQ	0.0493	0.2091	0.0840	0.0290	0.2232
432	CVIAWNSNN	0.0651	0.2766	0.0343	-1.1790	0.2228
209	PINLVRDLP	0.0553	0.2346	0.0298	-0.3310	0.2225
1246	GCCSCGSCC	0.0519	0.2203	0.0257	-0.0340	0.2225
403	RGDEVRQIA	0.0566	0.2402	0.1171	-0.7250	0.2215
1223	GLIAIVMVT	0.0581	0.2468	0.0446	-0.6420	0.2214
667	GAGICASYQ	0.0557	0.2365	0.0245	-0.3860	0.2209
1098	NGTHWFVTQ	0.0475	0.2016	0.2300	-0.3170	0.2203
715	PTNFTISVT	0.0659	0.2797	0.0341	-1.2920	0.2202
863	PLLTDEMIA	0.0533	0.2265	0.3241	-1.1230	0.2189
159	VYSSANNCT	0.0532	0.2259	0.0375	-0.2510	0.2189
472	IYQAGSTPC	0.0479	0.2034	0.0676	0.1030	0.2187
613	QDVNCTEVP	0.0525	0.2228	0.0248	-0.1640	0.2183
299	TKCTLKSFT	0.0604	0.2564	0.0232	-0.8350	0.2182
598	ITPGTNTSN	0.0637	0.2704	0.0761	-1.2820	0.2177
657	NNSYECDIP	0.0500	0.2123	0.0226	0.0320	0.2172
499	PTNGVGYQP	0.0564	0.2394	0.0306	-0.5400	0.2170
876	ALLAGTITS	0.0653	0.2773	0.2669	-2.0100	0.2169
708	SNNSIAIPT	0.0613	0.2601	0.0283	-0.9510	0.2168
253	DSSSGWTAG	0.0694	0.2945	0.0505	-1.7050	0.2168
174	PFLMDLEGK	0.0460	0.1952	0.1028	0.1210	0.2167
1214	WYIWLGFIA	0.0541	0.2297	0.0356	-0.3740	0.2163
140	FLGVYYHKN	0.0688	0.2920	0.0603	-1.6990	0.2161
1205	KYEQYIKWP	0.0465	0.1973	0.0247	0.3020	0.2161
37	YYPDKVFRS	0.0615	0.2611	0.3541	-1.9690	0.2158
358	ISNCVADYS	0.0752	0.3192	0.0233	-2.1440	0.2155
275	FLLKYNENG	0.0665	0.2823	0.0333	-1.4430	0.2151
451	YLYRLFRKS	0.0663	0.2814	0.1964	-1.9180	0.2150
594	GVSVITPGT	0.0554	0.2352	0.1247	-0.7800	0.2149

129	KVCEFQFCN	0.0620	0.2631	0.0294	-1.0520	0.2149
117	LLIVNNATN	0.0634	0.2694	0.0257	-1.1690	0.2148
355	RKRISNCVA	0.0486	0.2064	0.0707	-0.0520	0.2144
990	EVQIDRLIT	0.0570	0.2420	0.0281	-0.6490	0.2138
591	SFGGVSVIT	0.0554	0.2354	0.0738	-0.6550	0.2138
74	NGTKRFDNP	0.0510	0.2165	0.0258	-0.1510	0.2128
676	TQTNSPRRA	0.0524	0.2224	0.1235	-0.5660	0.2127
779	QEVFAQVKQ	0.0499	0.2117	0.0508	-0.1400	0.2123
426	PDDFTGCVI	0.0449	0.1908	0.2701	-0.3810	0.2123
466	RDISTEIYQ	0.0515	0.2188	0.0440	-0.2670	0.2120
340	EVFNATRFA	0.0541	0.2298	0.0532	-0.5190	0.2118
1134	NNTVYDPLQ	0.0514	0.2180	0.0243	-0.2100	0.2112
1015	AAEIRASAN	0.0622	0.2641	0.0240	-1.1310	0.2112
85	PFNDGVYFA	0.0441	0.1871	0.4569	-0.8940	0.2109
900	MQMAYRFNG	0.0612	0.2597	0.0234	-1.0540	0.2106
1196	SLIDLQELG	0.0653	0.2771	0.0297	-1.4210	0.2105
485	GFNCYFPLQ	0.0497	0.2108	0.1232	-0.3780	0.2104
1203	LGKYEQYIK	0.0469	0.1989	0.0657	0.0330	0.2104
1069	PAQEKNFTT	0.0568	0.2410	0.1658	-1.1180	0.2100
38	YPDKVFRSS	0.0688	0.2920	0.3851	-2.7980	0.2098
1108	NFYEPQIIT	0.0501	0.2128	0.0407	-0.1820	0.2098
738	CTMYICGDS	0.0765	0.3247	0.0258	-2.3820	0.2095
724	TEILPVSMT	0.0551	0.2342	0.0953	-0.7830	0.2093
480	CNGVEGFNC	0.0541	0.2298	0.0238	-0.4870	0.2090
927	FNSAIGKIQ	0.0530	0.2251	0.0271	-0.4060	0.2089
293	LDPLSETKC	0.0495	0.2101	0.0402	-0.1500	0.2086
541	FNFNGLTGT	0.0557	0.2365	0.0455	-0.7000	0.2083
881	TITSGWTFG	0.0616	0.2617	0.0948	-1.3540	0.2082
498	QPTNGVGYQ	0.0530	0.2251	0.0610	-0.5380	0.2073
165	NCTFEYVSQ	0.0472	0.2002	0.0276	0.0570	0.2072
562	FQQFGRDIA	0.0562	0.2386	0.0313	-0.7320	0.2067
386	KLNDLCFTN	0.0608	0.2583	0.0807	-1.2730	0.2067
373	SFSTFKCYG	0.0594	0.2522	0.0372	-1.0250	0.2065
599	TPGTNTSNQ	0.0518	0.2199	0.1044	-0.5850	0.2063
483	VEGFNCYFP	0.0494	0.2096	0.0322	-0.1620	0.2063
668	AGICASYQT	0.0541	0.2296	0.1069	-0.7890	0.2062
210	INLVRDLPQ	0.0511	0.2171	0.0467	-0.3620	0.2060
106	FGTTLDSKT	0.0603	0.2561	0.0309	-1.0940	0.2060
99	NIIRGWIFG	0.0607	0.2575	0.0245	-1.1070	0.2059
107	GTTLDSKTQ	0.0518	0.2197	0.0563	-0.4470	0.2058
761	TQLNRALTG	0.0621	0.2637	0.0508	-1.3300	0.2048

252	GDSSSGWTA	0.0582	0.2472	0.1046	-1.1710	0.2044
23	QLPPAYTNS	0.0652	0.2766	0.2578	-2.2180	0.2044
423	YKLPDDFTG	0.0608	0.2582	0.0403	-1.2070	0.2039
1103	FVTQRNFYE	0.0662	0.2812	0.0227	-1.6140	0.2039
961	TLVKQLSSN	0.0594	0.2522	0.0424	-1.0980	0.2036
756	YGSFCTQLN	0.0663	0.2814	0.0332	-1.6550	0.2036
473	YQAGSTPCN	0.0615	0.2613	0.0263	-1.2340	0.2036
377	FKCYGVSPT	0.0556	0.2360	0.0280	-0.7320	0.2036
624	IHADQLTPT	0.0507	0.2153	0.0892	-0.5040	0.2034
278	KYNENGTIT	0.0472	0.2005	0.1459	-0.3800	0.2034
490	FPLQSYGFQ	0.0529	0.2247	0.0315	-0.5390	0.2024
1072	EKNFTTAPA	0.0485	0.2061	0.2529	-0.8330	0.2024
470	TEIYQAGST	0.0554	0.2352	0.0257	-0.7390	0.2021
207	HTPINLVRD	0.0676	0.2870	0.0482	-1.8420	0.2021
411	APGQTGKIA	0.0490	0.2079	0.2443	-0.8500	0.2020
1110	YEPQIITTD	0.0661	0.2806	0.1719	-2.0910	0.2018
86	FNDGVYFAS	0.0793	0.3368	0.0360	-2.8140	0.2015
1265	LKGVKLHYT	0.0522	0.2217	0.1187	-0.7680	0.2011
564	QFGRDIADT	0.0548	0.2328	0.0295	-0.7370	0.2004
615	VNCTEVPVA	0.0539	0.2287	0.0599	-0.7560	0.1999
523	TVCGPKKST	0.0504	0.2141	0.0467	-0.4250	0.1999
805	ILPDPSKPS	0.0502	0.2132	0.6659	-2.2670	0.1998
301	CTLKSFTVE	0.0638	0.2707	0.0299	-1.5090	0.1998
595	VSVITPGTN	0.0597	0.2533	0.0297	-1.1650	0.1995
492	LQSYGFQPT	0.0539	0.2289	0.0603	-0.7750	0.1992
434	IAWNSNNLD	0.0604	0.2565	0.1235	-1.5210	0.1990
346	RFASVYAWN	0.0563	0.2390	0.0234	-0.8770	0.1987
231	IGINITRFQ	0.0508	0.2156	0.0271	-0.4250	0.1985
188	NLREFVFKN	0.0583	0.2474	0.0390	-1.1090	0.1978
994	DRLITGRLQ	0.0473	0.2009	0.0410	-0.1880	0.1977
1166	LGDISGINA	0.0575	0.2442	0.0607	-1.1130	0.1977
991	VQIDRLITG	0.0583	0.2476	0.1177	-1.3560	0.1975
506	QPYRVVVLS	0.0576	0.2448	0.4855	-2.4020	0.1975
776	KNTQEVFAQ	0.0494	0.2096	0.0262	-0.3260	0.1972
555	SNKKFLPFQ	0.0487	0.2068	0.0607	-0.3740	0.1972
644	QTRAGCLIG	0.0625	0.2654	0.0316	-1.4600	0.1971
104	WIFGTTLDS	0.0674	0.2861	0.0858	-2.0620	0.1959
385	TKLNDLCFT	0.0545	0.2316	0.0289	-0.8040	0.1957
765	RALTGIAVE	0.0569	0.2417	0.0621	-1.1100	0.1955
199	GYFKIYSKH	0.0472	0.2003	0.1244	-0.4770	0.1951
713	AIPTNFTIS	0.0645	0.2737	0.1350	-1.9790	0.1950

942	ASALGKLQD	0.0638	0.2708	0.0291	-1.6040	0.1949
537	KCVNFNFNG	0.0571	0.2423	0.0337	-1.0490	0.1949
696	TMSLGAENS	0.0708	0.3005	0.0272	-2.2010	0.1945
995	RLITGRLQS	0.0687	0.2918	0.0347	-2.0520	0.1944
1080	AICHDGKAH	0.0495	0.2102	0.0254	-0.3990	0.1941
406	EVRQIAPGQ	0.0479	0.2032	0.0259	-0.2610	0.1940
631	PTWRVYSTG	0.0569	0.2417	0.2508	-1.7150	0.1936
408	RQIAPGQTG	0.0552	0.2343	0.0484	-0.9600	0.1936
200	YFKIYSKHT	0.0509	0.2161	0.0437	-0.5900	0.1932
1188	EVAKNLNES	0.0676	0.2871	0.0916	-2.1530	0.1932
1160	TSPDVDLGD	0.0660	0.2802	0.0314	-1.8340	0.1932
703	NSVAYSNNS	0.0708	0.3006	0.0315	-2.2570	0.1925
407	VRQIAPGQT	0.0516	0.2192	0.0342	-0.6360	0.1925
114	TQSLLIVNN	0.0581	0.2467	0.0269	-1.1640	0.1925
1218	LGFIAGLIA	0.0530	0.2252	0.0278	-0.7420	0.1922
967	SSNFGAISS	0.0693	0.2941	0.0522	-2.1980	0.1920
823	FNKVTLADA	0.0523	0.2219	0.0453	-0.7370	0.1918
946	GKLQDVVNQ	0.0458	0.1946	0.0372	-0.1690	0.1917
404	GDEVRQIAP	0.0489	0.2076	0.0248	-0.3940	0.1916
91	YFASTEKSN	0.0573	0.2431	0.0228	-1.1040	0.1914
244	LHRSYLTPG	0.0502	0.2131	0.2486	-1.1810	0.1914
763	LNRALTGIA	0.0516	0.2189	0.0417	-0.6840	0.1909
515	FELLHAPAT	0.0540	0.2295	0.0306	-0.8640	0.1909
565	FGRDIADTT	0.0547	0.2322	0.0793	-1.0670	0.1907
232	GINITRFQT	0.0530	0.2252	0.0504	-0.8410	0.1907
1058	HGVVFLHVT	0.0561	0.2383	0.0580	-1.1300	0.1905
65	FHAIHVSGT	0.0531	0.2255	0.0427	-0.8320	0.1903
1118	DNTFVSGNC	0.0496	0.2107	0.0240	-0.4800	0.1903
749	CSNLLLQYG	0.0629	0.2670	0.0402	-1.6570	0.1902
816	SFIEDLLFN	0.0544	0.2308	0.0259	-0.8940	0.1900
146	HKNNKSWME	0.0642	0.2726	0.0338	-1.7530	0.1900
917	YENQKLIAN	0.0613	0.2602	0.0329	-1.5170	0.1893
566	GRDIADTTD	0.0526	0.2231	0.4627	-2.0690	0.1891
899	AMQMAYRFN	0.0576	0.2444	0.0224	-1.1870	0.1885
176	LMDLEGKQG	0.0615	0.2609	0.0314	-1.5510	0.1881
918	ENQKLIANQ	0.0493	0.2093	0.0343	-0.5460	0.1872
949	QDVVNQNAQ	0.0463	0.1964	0.0295	-0.2740	0.1871
67	AIHVSGTNG	0.0556	0.2361	0.0451	-1.1160	0.1871
608	VAVLYQDVN	0.0569	0.2417	0.0259	-1.1720	0.1870
173	QPFLMDLEG	0.0583	0.2475	0.1033	-1.5210	0.1870
1077	TAPAICHDG	0.0568	0.2411	0.0941	-1.3690	0.1867

770	IAVEQDKNT	0.0498	0.2114	0.0290	-0.5820	0.1866
556	NKKFLPFQQ	0.0412	0.1747	0.0672	0.0330	0.1865
27	AYTNSFTRG	0.0500	0.2122	0.2181	-1.1680	0.1865
872	QYTSALLAG	0.0542	0.2300	0.0421	-1.0000	0.1863
1040	VDFCGKGYH	0.0482	0.2046	0.0565	-0.5370	0.1862
1027	TKMSECVLG	0.0561	0.2380	0.0351	-1.1480	0.1859
468	ISTEIYQAG	0.0571	0.2424	0.1249	-1.5070	0.1858
885	GWTFGAGAA	0.0468	0.1987	0.0619	-0.4500	0.1855
1023	NLAATKMSE	0.0579	0.2457	0.0439	-1.3380	0.1854
45	SSVLHSTQD	0.0587	0.2492	0.0763	-1.5120	0.1851
729	VSMTKTSVD	0.0635	0.2694	0.0323	-1.7930	0.1846
249	LTPGDSSSG	0.0563	0.2390	0.1257	-1.4800	0.1838
548	GTGVLTESN	0.0618	0.2625	0.0285	-1.6690	0.1833
528	KKSTNLVKN	0.0571	0.2424	0.0401	-1.3080	0.1830
332	ITNLCPFGE	0.0602	0.2555	0.0224	-1.5170	0.1830
367	VLYNSASFS	0.0644	0.2734	0.0370	-1.9230	0.1828
22	TQLPPAYTN	0.0515	0.2188	0.1301	-1.1110	0.1828
172	SQPFLMDLE	0.0588	0.2494	0.0230	-1.4050	0.1826
650	LIGAEHVNN	0.0578	0.2455	0.0306	-1.3530	0.1824
1100	THWFVTQRN	0.0501	0.2129	0.1921	-1.1870	0.1824
879	AGTITSGWT	0.0514	0.2184	0.0300	-0.8160	0.1821
646	RAGCLIGAE	0.0588	0.2496	0.0237	-1.4220	0.1820
1163	DVDLGDISG	0.0637	0.2703	0.0413	-1.8940	0.1818
649	CLIGAEHVN	0.0558	0.2368	0.0653	-1.2980	0.1817
1174	ASVVNIQKE	0.0552	0.2345	0.0354	-1.1620	0.1817
597	VITPGTNTS	0.0599	0.2543	0.1946	-2.0370	0.1816
802	FSQILPDPS	0.0676	0.2868	0.0259	-2.1910	0.1812
767	LTGIAVEQD	0.0631	0.2679	0.0344	-1.8420	0.1810
790	KTPPIKDFG	0.0578	0.2456	0.0244	-1.3770	0.1804
508	YRVVVLSFE	0.0581	0.2468	0.0393	-1.4460	0.1804
970	FGAISSVLN	0.0592	0.2515	0.0289	-1.5190	0.1799
274	TFLLKYNEN	0.0532	0.2258	0.0314	-1.0190	0.1796
326	IVRFPNITN	0.0515	0.2187	0.0885	-1.0510	0.1794
775	DKNTQEVFA	0.0517	0.2193	0.0480	-0.9450	0.1793
952	VNQNAQALN	0.0574	0.2438	0.0299	-1.3910	0.1787
1150	EELDKYFKN	0.0499	0.2119	0.2904	-1.5360	0.1786
246	RSYLTPGDS	0.0615	0.2613	0.0442	-1.7970	0.1781
488	CYFPLQSYG	0.0463	0.1966	0.1825	-0.9190	0.1780
534	VKNKCVNFN	0.0557	0.2366	0.0241	-1.2500	0.1778
998	TGRLQSLQT	0.0511	0.2169	0.0482	-0.9350	0.1774
1231	TIMLCCMTS	0.0628	0.2668	0.0786	-2.0260	0.1773

672	ASYQTQTNS	0.0620	0.2632	0.0515	-1.8810	0.1768
558	KFLPFQQFG	0.0521	0.2210	0.0394	-1.0020	0.1768
87	NDGVYFAST	0.0537	0.2278	0.0707	-1.2440	0.1762
929	SAIGKIQDS	0.0619	0.2629	0.0632	-1.9300	0.1759
211	NLVRDLPQG	0.0514	0.2183	0.1347	-1.2520	0.1759
769	GIAVEQDKN	0.0564	0.2393	0.0227	-1.3390	0.1758
701	AENSVAYSN	0.0569	0.2415	0.0354	-1.4310	0.1753
64	WFHAIHVSG	0.0541	0.2297	0.0412	-1.2150	0.1751
849	LICAQKFNG	0.0566	0.2403	0.0277	-1.3930	0.1748
532	NLVKNKCVN	0.0540	0.2292	0.0266	-1.1710	0.1747
1252	SCCKFDEDD	0.0610	0.2589	0.0243	-1.7630	0.1743
838	GDCLGDIAA	0.0511	0.2170	0.0698	-1.0650	0.1742
984	LDKVEAEVQ	0.0434	0.1841	0.0266	-0.2790	0.1741
1165	DLGDISGIN	0.0599	0.2544	0.0400	-1.7280	0.1740
1241	CSCLKGCCS	0.0665	0.2823	0.0253	-2.2440	0.1739
1170	SGINASVVN	0.0567	0.2405	0.0346	-1.4510	0.1732
380	YGVSPTKLN	0.0575	0.2442	0.0340	-1.5310	0.1727
840	CLGDIAARD	0.0634	0.2690	0.0681	-2.1390	0.1723
476	GSTPCNGVE	0.0583	0.2477	0.0245	-1.5860	0.1721
866	TDEMIAQYT	0.0527	0.2236	0.0309	-1.1300	0.1717
1064	HVTYVPAQE	0.0570	0.2422	0.0324	-1.5060	0.1717
474	QAGSTPCNG	0.0577	0.2449	0.0241	-1.5450	0.1713
1131	GIVNNTVYD	0.0537	0.2282	0.2139	-1.7790	0.1713
910	GVTQNVLYE	0.0583	0.2474	0.0368	-1.6330	0.1712
633	WRVYSTGSN	0.0514	0.2180	0.0369	-1.0480	0.1712
276	LLKYNENGT	0.0486	0.2064	0.0287	-0.7890	0.1712
260	AGAAAYYVG	0.0561	0.2382	0.0499	-1.4910	0.1712
1068	VPAQEKNFT	0.0508	0.2159	0.0283	-0.9830	0.1710
860	VLPPLLTDE	0.0553	0.2347	0.0712	-1.4890	0.1709
536	NKCVNFNFN	0.0524	0.2225	0.0257	-1.1190	0.1705
134	QFCNDPFLG	0.0546	0.2317	0.0272	-1.3110	0.1702
920	QKLIANQFN	0.0521	0.2212	0.0234	-1.0940	0.1700
273	RTFLLKYNE	0.0553	0.2350	0.0255	-1.3760	0.1700
573	TDAVRDPQT	0.0507	0.2155	0.0311	-1.0070	0.1698
1243	CLKGCCSCG	0.0506	0.2148	0.1537	-1.3650	0.1696
53	DLFLPFFSN	0.0542	0.2301	0.1104	-1.5490	0.1692
32	FTRGVYYPD	0.0599	0.2542	0.0400	-1.8290	0.1688
824	NKVTLADAG	0.0545	0.2316	0.0248	-1.3360	0.1685
352	AWNRKRISN	0.0513	0.2177	0.0285	-1.0710	0.1684
356	KRISNCVAD	0.0534	0.2268	0.0428	-1.3010	0.1682
540	NFNFNGLTG	0.0523	0.2219	0.0534	-1.2380	0.1680

1186	LNEVAKNLN	0.0559	0.2372	0.0258	-1.4760	0.1673
4	FLVLLPLVS	0.0636	0.2700	0.0467	-2.1960	0.1672
1184	DRLNEVAKN	0.0527	0.2236	0.0904	-1.3980	0.1672
154	ESEFRVYSS	0.0674	0.2863	0.0368	-2.5170	0.1659
405	DEVRQIAPG	0.0528	0.2242	0.2087	-1.7990	0.1656
95	TEKSNIIRG	0.0552	0.2343	0.0405	-1.5000	0.1654
1051	SFPQSAPHG	0.0516	0.2189	0.0256	-1.1540	0.1650
351	YAWNRKRIS	0.0608	0.2581	0.0424	-1.9940	0.1648
1136	TVYDPLQPE	0.0499	0.2117	0.0608	-1.1200	0.1648
859	TVLPPLLTD	0.0553	0.2348	0.0760	-1.6320	0.1646
203	IYSKHTPIN	0.0477	0.2025	0.0569	-0.9310	0.1645
619	EVPVAIHAD	0.0555	0.2358	0.1527	-1.8860	0.1644
1036	QSKRVDFCG	0.0537	0.2279	0.0337	-1.3740	0.1643
297	SETKCTLKS	0.0639	0.2712	0.0572	-2.3160	0.1640
108	TTLDSKTQS	0.0647	0.2747	0.0276	-2.3000	0.1639
683	RARSVASQS	0.0585	0.2483	0.0258	-1.7680	0.1638
740	MYICGDSTE	0.0506	0.2147	0.0521	-1.1750	0.1637
1155	YFKNHTSPD	0.0557	0.2366	0.0363	-1.5690	0.1636
1089	FPREGVFVS	0.0516	0.2189	0.5008	-2.6190	0.1630
822	LFNKVTLAD	0.0569	0.2416	0.0374	-1.6900	0.1627
542	NFNGLTGTG	0.0528	0.2243	0.0516	-1.3900	0.1625
1091	REGVFVSNG	0.0561	0.2382	0.0270	-1.5950	0.1625
1092	EGVFVSNGT	0.0487	0.2067	0.0271	-0.9660	0.1624
1127	DVVIGIVNN	0.0522	0.2218	0.0777	-1.4220	0.1623
1251	GSCCKFDED	0.0602	0.2555	0.0259	-1.9520	0.1618
928	NSAIGKIQD	0.0573	0.2434	0.0645	-1.8260	0.1617
560	LPFQQFGRD	0.0528	0.2242	0.2835	-2.1090	0.1613
986	KVEAEVQID	0.0588	0.2499	0.0240	-1.8530	0.1608
422	NYKLPDDFT	0.0419	0.1781	0.0243	-0.4580	0.1588
1179	IQKEIDRLN	0.0491	0.2083	0.0280	-1.0780	0.1586
736	VDCTMYICG	0.0549	0.2330	0.0291	-1.5780	0.1585
337	PFGEVFNAT	0.0494	0.2099	0.0525	-1.1880	0.1584
963	VKQLSSNFG	0.0511	0.2168	0.0314	-1.2680	0.1581
848	DLICAQKFN	0.0529	0.2247	0.0258	-1.4110	0.1581
303	LKSFTVEKG	0.0520	0.2207	0.0266	-1.3360	0.1579
1066	TYVPAQEKN	0.0458	0.1946	0.0710	-0.9470	0.1579
921	KLIANQFNS	0.0597	0.2537	0.0591	-2.0990	0.1576
1119	NTFVSGNCD	0.0577	0.2450	0.0235	-1.8270	0.1572
435	AWNSNNLDS	0.0587	0.2494	0.0262	-1.9400	0.1564
331	NITNLCPFG	0.0526	0.2233	0.0242	-1.4110	0.1564
575	AVRDPQTLE	0.0509	0.2161	0.0589	-1.3730	0.1563

73	TNGTKRFDN	0.0558	0.2368	0.0228	-1.6820	0.1562
586	DITPCSFGG	0.0539	0.2290	0.0388	-1.5710	0.1562
245	HRSYLTPGD	0.0532	0.2258	0.0847	-1.6560	0.1557
157	FRVYSSANN	0.0509	0.2162	0.0274	-1.2930	0.1557
103	GWIFGTTLD	0.0492	0.2090	0.1551	-1.5470	0.1549
791	TPPIKDFGG	0.0499	0.2120	0.1631	-1.6350	0.1547
835	KQYGDCLGD	0.0518	0.2201	0.0416	-1.4370	0.1544
800	FNFSQILPD	0.0582	0.2473	0.0405	-1.9810	0.1543
518	LHAPATVCG	0.0506	0.2149	0.0356	-1.3190	0.1543
153	MESEFRVYS	0.0626	0.2658	0.0551	-2.3940	0.1543
524	VCGPKKSTN	0.0517	0.2194	0.0244	-1.3860	0.1538
722	VTTEILPVS	0.0605	0.2571	0.0396	-2.1870	0.1537
700	GAENSVAYS	0.0640	0.2719	0.0341	-2.4680	0.1536
606	NQVAVLYQD	0.0531	0.2257	0.0423	-1.5710	0.1535
906	FNGIGVTQN	0.0538	0.2286	0.0285	-1.5940	0.1532
1117	TDNTFVSGN	0.0553	0.2350	0.0297	-1.7280	0.1530
709	NNSIAIPTN	0.0512	0.2175	0.0291	-1.3810	0.1529
335	LCPFGEVFN	0.0488	0.2073	0.0255	-1.1650	0.1529
398	DSFVIRGDE	0.0537	0.2280	0.0239	-1.5750	0.1528
1126	CDVVIGIVN	0.0517	0.2195	0.0359	-1.4450	0.1527
593	GGVSVITPG	0.0534	0.2269	0.1271	-1.8680	0.1525
419	ADYNYKLPD	0.0552	0.2345	0.0462	-1.7830	0.1523
655	HVNNSYECD	0.0589	0.2502	0.0232	-2.0290	0.1522
611	LYQDVNCTE	0.0496	0.2106	0.0383	-1.2820	0.1522
309	EKGIYQTSN	0.0512	0.2173	0.0944	-1.5860	0.1522
1112	PQIITTDNT	0.0490	0.2079	0.0281	-1.2080	0.1517
191	EFVFKNIDG	0.0490	0.2083	0.0270	-1.2220	0.1512
982	SRLDKVEAE	0.0491	0.2084	0.0299	-1.2400	0.1509
323	TESIVRFPN	0.0535	0.2270	0.0315	-1.6210	0.1507
834	IKQYGDCLG	0.0515	0.2187	0.0319	-1.4600	0.1505
819	EDLLFNKVT	0.0479	0.2033	0.0410	-1.1900	0.1500
1138	YDPLQPELD	0.0585	0.2486	0.0459	-2.1110	0.1499
316	SNFRVQPTE	0.0512	0.2175	0.0625	-1.5430	0.1497
442	DSKVGGNYN	0.0525	0.2230	0.0253	-1.5450	0.1495
390	LCFTNVYAD	0.0550	0.2337	0.0419	-1.8180	0.1491
971	GAISSVLND	0.0573	0.2433	0.0287	-1.9780	0.1487
251	PGDSSSGWT	0.0529	0.2247	0.0270	-1.6180	0.1479
213	VRDLPQGFS	0.0566	0.2401	0.0831	-2.1050	0.1474
1194	NESLIDLQE	0.0526	0.2234	0.0328	-1.6200	0.1473
1191	KNLNESLID	0.0535	0.2269	0.0289	-1.6890	0.1468
156	EFRVYSSAN	0.0478	0.2029	0.0418	-1.2490	0.1467

9	PLVSSQCVN	0.0522	0.2217	0.0491	-1.6500	0.1466
1009	TQQLIRAAE	0.0513	0.2177	0.0233	-1.4960	0.1464
563	QQFGRDIAD	0.0529	0.2248	0.0483	-1.7200	0.1460
1152	LDKYFKNHT	0.0450	0.1912	0.0913	-1.1780	0.1460
459	SNLKPFERD	0.0541	0.2296	0.0404	-1.7960	0.1459
177	MDLEGKQGN	0.0509	0.2160	0.0358	-1.5100	0.1459
190	REFVFKNID	0.0515	0.2186	0.0279	-1.5470	0.1455
585	LDITPCSFG	0.0517	0.2195	0.0434	-1.6210	0.1450
924	ANQFNSAIG	0.0494	0.2096	0.0443	-1.4270	0.1449
795	KDFGGFNFS	0.0481	0.2042	0.3795	-2.3250	0.1449
1187	NEVAKNLNE	0.0513	0.2180	0.0253	-1.5380	0.1449
5	LVLLPLVSS	0.0546	0.2316	0.1509	-2.1900	0.1448
431	GCVIAWNSN	0.0490	0.2080	0.0407	-1.3860	0.1448
1142	QPELDSFKE	0.0484	0.2053	0.2439	-1.9530	0.1443
457	RKSNLKPFE	0.0494	0.2096	0.0228	-1.3810	0.1440
743	CGDSTECSN	0.0540	0.2293	0.0249	-1.7900	0.1435
659	SYECDIPIG	0.0452	0.1920	0.0315	-1.0810	0.1427
25	PPAYTNSFT	0.0488	0.2071	0.0431	-1.4250	0.1423
702	ENSVAYSNN	0.0504	0.2140	0.0265	-1.5220	0.1419
830	DAGFIKQYG	0.0510	0.2165	0.0561	-1.6680	0.1415
81	NPVLPFNDG	0.0495	0.2101	0.1170	-1.7240	0.1415
486	FNCYFPLQS	0.0620	0.2631	0.0252	-2.5140	0.1412
661	ECDIPIGAG	0.0532	0.2258	0.0335	-1.8070	0.1405
588	TPCSFGGVS	0.0603	0.2560	0.0772	-2.5530	0.1400
439	NNLDSKVGG	0.0489	0.2078	0.0244	-1.4390	0.1395
216	LPQGFSALE	0.0531	0.2253	0.1066	-2.0370	0.1395
494	SYGFQPTNG	0.0462	0.1961	0.0512	-1.2900	0.1393
1176	VVNIQKEID	0.0514	0.2181	0.0244	-1.6550	0.1390
294	DPLSETKCT	0.0456	0.1937	0.0500	-1.2600	0.1382
1123	SGNCDVVIG	0.0512	0.2173	0.0239	-1.6560	0.1381
42	VFRSSVLHS	0.0531	0.2253	0.0356	-1.8660	0.1373
640	SNVFQTRAG	0.0481	0.2042	0.0350	-1.4470	0.1371
653	AEHVNNSYE	0.0492	0.2088	0.0380	-1.5570	0.1367
980	ILSRLDKVE	0.0504	0.2139	0.0345	-1.6660	0.1357
79	FDNPVLPFN	0.0507	0.2152	0.0292	-1.6800	0.1356
164	NNCTFEYVS	0.0569	0.2414	0.0247	-2.1980	0.1352
148	NNKSWMESE	0.0496	0.2104	0.0248	-1.5820	0.1350
147	KNNKSWMES	0.0590	0.2504	0.0327	-2.4080	0.1349
811	KPSKRSFIE	0.0497	0.2110	0.0629	-1.7130	0.1348
1013	IRAAEIRAS	0.0538	0.2286	0.0248	-1.9510	0.1347
452	LYRLFRKSN	0.0428	0.1818	0.0334	-1.0500	0.1343

247	SYLTPGDSS	0.0498	0.2113	0.0796	-1.7780	0.1343
381	GVSPTKLND	0.0530	0.2249	0.0327	-1.9120	0.1342
391	CFTNVYADS	0.0553	0.2348	0.0299	-2.1070	0.1339
496	GFQPTNGVG	0.0476	0.2022	0.0411	-1.4910	0.1338
130	VCEFQFCND	0.0516	0.2190	0.0234	-1.7760	0.1337
793	PIKDFGGFN	0.0510	0.2166	0.0430	-1.8140	0.1324
1111	EPQIITTDN	0.0477	0.2024	0.1669	-1.9080	0.1320
183	QGNFKNLRE	0.0515	0.2188	0.0244	-1.8120	0.1319
1259	DDSEPVLKG	0.0514	0.2184	0.1527	-2.1900	0.1318
772	VEQDKNTQE	0.0472	0.2003	0.0399	-1.5170	0.1304
1085	GKAHFPREG	0.0489	0.2075	0.0321	-1.6470	0.1300
694	AYTMSLGAE	0.0437	0.1856	0.0230	-1.1840	0.1298
1254	CKFDEDDSE	0.0468	0.1988	0.0230	-1.4520	0.1297
977	LNDILSRLD	0.0542	0.2302	0.0263	-2.0930	0.1295
945	LGKLQDVVN	0.0458	0.1946	0.0383	-1.4260	0.1291
570	ADTTDAVRD	0.0530	0.2251	0.0274	-2.0060	0.1289
717	NFTISVTTE	0.0455	0.1930	0.0294	-1.3870	0.1281
1090	PREGVFVSN	0.0486	0.2062	0.0919	-1.8380	0.1281
438	SNNLDSKVG	0.0462	0.1963	0.0248	-1.4480	0.1277
289	VDCALDPLS	0.0566	0.2403	0.0246	-2.3280	0.1276
1180	QKEIDRLNE	0.0481	0.2042	0.0225	-1.6380	0.1256
1249	SCGSCCKFD	0.0511	0.2172	0.0231	-1.9120	0.1250
1234	LCCMTSCCS	0.0541	0.2295	0.0284	-2.1760	0.1250
430	TGCVIAWNS	0.0596	0.2530	0.0227	-2.6290	0.1249
1033	VLGQSKRVD	0.0508	0.2157	0.0285	-1.9040	0.1248
363	ADYSVLYNS	0.0534	0.2269	0.0408	-2.1750	0.1243
1250	CGSCCKFDE	0.0499	0.2120	0.0222	-1.8230	0.1242
124	TNVVIKVCE	0.0462	0.1960	0.0239	-1.5070	0.1242
308	VEKGIYQTS	0.0486	0.2063	0.2190	-2.3110	0.1236
737	DCTMYICGD	0.0529	0.2247	0.0248	-2.1000	0.1234
547	TGTGVLTES	0.0565	0.2397	0.0572	-2.5210	0.1223
141	LGVYYHKNN	0.0466	0.1980	0.0281	-1.6050	0.1220
788	IYKTPPIKD	0.0443	0.1879	0.0850	-1.5790	0.1217
290	DCALDPLSE	0.0487	0.2070	0.0262	-1.7970	0.1210
1157	KNHTSPDVD	0.0504	0.2139	0.0256	-1.9360	0.1209
1244	LKGCCSCGS	0.0549	0.2333	0.0339	-2.3620	0.1203
1022	ANLAATKMS	0.0526	0.2234	0.0383	-2.1820	0.1201
522	ATVCGPKKS	0.0530	0.2251	0.0247	-2.1900	0.1193
1047	YHLMSFPQS	0.0539	0.2289	0.0259	-2.2700	0.1192
90	VYFASTEKS	0.0482	0.2048	0.0361	-1.8370	0.1183
578	DPQTLEILD	0.0537	0.2282	0.0437	-2.3360	0.1180

341	VFNATRFAS	0.0538	0.2282	0.0226	-2.2770	0.1178
812	PSKRSFIED	0.0521	0.2212	0.0286	-2.1560	0.1176
143	VYYHKNNKS	0.0456	0.1935	0.0799	-1.7620	0.1174
397	ADSFVIRGD	0.0491	0.2083	0.0718	-2.0350	0.1173
1043	CGKGYHLMS	0.0573	0.2432	0.0449	-2.6600	0.1170
632	TWRVYSTGS	0.0514	0.2183	0.0286	-2.1160	0.1168
1199	DLQELGKYE	0.0488	0.2072	0.0253	-1.8850	0.1168
180	EGKQGNFKN	0.0468	0.1987	0.0312	-1.7400	0.1164
1153	DKYFKNHTS	0.0499	0.2120	0.1085	-2.2770	0.1144
286	TDAVDCALD	0.0509	0.2160	0.0264	-2.1180	0.1140
932	GKIQDSLSS	0.0519	0.2205	0.0482	-2.2970	0.1128
959	LNTLVKQLS	0.0524	0.2224	0.0268	-2.2760	0.1126
750	SNLLLQYGS	0.0528	0.2241	0.0319	-2.3340	0.1122
831	AGFIKQYGD	0.0468	0.1988	0.0773	-1.9650	0.1121
665	PIGAGICAS	0.0563	0.2389	0.0602	-2.7510	0.1103
651	IGAEHVNNS	0.0526	0.2235	0.0533	-2.4240	0.1103
88	DGVYFASTE	0.0476	0.2023	0.0243	-1.9290	0.1095
935	QDSLSSTAS	0.0526	0.2235	0.0844	-2.5350	0.1094
63	TWFHAIHVS	0.0466	0.1977	0.0313	-1.8610	0.1093
461	LKPFERDIS	0.0491	0.2086	0.0229	-2.0660	0.1087
317	NFRVQPTES	0.0473	0.2010	0.0250	-1.9380	0.1079
583	EILDITPCS	0.0507	0.2153	0.0344	-2.2550	0.1077
479	PCNGVEGFN	0.0463	0.1964	0.0247	-1.8590	0.1071
727	LPVSMTKTS	0.0521	0.2212	0.0514	-2.4590	0.1059
72	GTNGTKRFD	0.0483	0.2051	0.0230	-2.0560	0.1058
224	EPLVDLPIG	0.0455	0.1931	0.0278	-1.8380	0.1053
678	TNSPRRARS	0.0525	0.2230	0.0359	-2.4720	0.1048
867	DEMIAQYTS	0.0530	0.2251	0.0619	-2.6220	0.1033
272	PRTFLLKYN	0.0421	0.1786	0.0490	-1.6740	0.1022
808	DPSKPSKRS	0.0483	0.2050	0.2969	-3.0150	0.0988
742	ICGDSTECS	0.0503	0.2137	0.0342	-2.4170	0.0980
463	PFERDISTE	0.0440	0.1869	0.0426	-1.9210	0.0972
1167	GDISGINAS	0.0525	0.2231	0.0290	-2.6150	0.0967
1145	LDSFKEELD	0.0482	0.2048	0.0267	-2.2900	0.0943
197	IDGYFKIYS	0.0504	0.2141	0.0278	-2.5520	0.0906
420	DYNYKLPDD	0.0417	0.1769	0.0232	-1.8070	0.0900
282	NGTITDAVD	0.0459	0.1949	0.0371	-2.2480	0.0881
80	DNPVLPFND	0.0479	0.2036	0.0235	-2.4040	0.0869
931	IGKIQDSLS	0.0466	0.1980	0.0376	-2.3370	0.0867
412	PGQTGKIAD	0.0480	0.2037	0.0247	-2.4400	0.0854
52	QDLFLPFFS	0.0486	0.2066	0.0264	-2.5120	0.0849

1139	DPLQPELDS	0.0496	0.2106	0.1247	-2.9000	0.0843
1253	CCKFDEDDS	0.0450	0.1912	0.0224	-2.2140	0.0839
681	PRRARSVAS	0.0465	0.1972	0.0259	-2.4160	0.0803
1084	DGKAHFPRE	0.0436	0.1853	0.0239	-2.1730	0.0802
1143	PELDSFKEE	0.0416	0.1765	0.0278	-2.2120	0.0701
1162	PDVDLGDIS	0.0440	0.1866	0.0234	-2.9010	0.0451

Table S2. List of predicted CTL from SARS-CoV-2 Main protease. → -E represents the MHC ligands and top epitopes.

NetCTL-1.2 predictions using MHC supertype A1. Threshold 0.750000

Number of MHC ligands 8 identified. Number of peptides 298. Protein name Mpro

----

Residue No.	Peptide Sequence	Predicted MHC binding affinity	Rescale binding affinity	C-terminal cleavage affinity	Transport efficiency	Prediction score	Identified MHC ligand
174	GTDLEGNFY	0.7930	3.3669	0.6229	2.7020	3.5954	<-E
201	TVNVLAWLY	0.6255	2.6559	0.8852	2.9570	2.9365	<-E
146	GSVGFNIDY	0.3112	1.3211	0.9565	2.8570	1.6075	<-E
110	QTFSVLACY	0.2625	1.1146	0.9725	2.9980	1.4104	<-E
153	DYDCVSFCY	0.2097	0.8905	0.9722	2.7060	1.1717	<-E
93	TANPKTPKY	0.1676	0.7118	0.9755	2.7230	0.9942	<-E
46	SEDMLNPNY	0.1528	0.6489	0.8406	2.6760	0.9088	<-E
_286	LLEDEFTPF	0.1132	0.4807	0.9503	2.5680	0.7517	<-E
261	VLDMCASLK	0.1397	0.5933	0.7881	0.5240	0.7377	
231	NLVAMKYNY	0.1073	0.4555	0.8757	2.9540	0.7345	
118	YNGSPSGVY	0.0999	0.4243	0.9564	2.5820	0.6969	
256	QTGIAVLDM	0.1269	0.5388	0.9157	0.2870	0.6905	
23	GTTTLNGLW	0.1311	0.5566	0.4256	0.6410	0.6525	
253	LSAQTGIAV	0.1426	0.6054	0.0941	0.2310	0.6311	
24	TTTLNGLWL	0.1293	0.5489	0.2161	0.8460	0.6237	
225	TTLNDFNLV	0.1106	0.4694	0.9195	0.3000	0.6223	
81	SMQNCVLKL	0.0952	0.4042	0.9581	1.0740	0.6016	
157	VSFCYMHHM	0.0996	0.4230	0.9507	0.5120	0.5912	
195	GTDTTITVN	0.1527	0.6485	0.0525	-1.5380	0.5794	
229	DFNLVAMKY	0.0669	0.2841	0.9722	2.8230	0.5711	
242	LTQDHVDIL	0.1109	0.4711	0.3794	0.7600	0.5660	
219	FLNRFTTTL	0.0917	0.3893	0.9334	0.7290	0.5658	
29	GLWLDDVVY	0.0620	0.2632	0.9766	3.0780	0.5636	
185	FVDRQTAQA	0.1082	0.4593	0.7828	-0.8130	0.5361	
151	NIDYDCVSF	0.0733	0.3111	0.6732	2.4190	0.5331	
279	RTILGSALL	0.0762	0.3234	0.9504	1.2160	0.5268	
283	GSALLEDEF	0.0921	0.3911	0.0575	2.4610	0.5228	
297	VRQCSGVTF	0.0554	0.2353	0.9711	2.8180	0.5219	
80	HSMQNCVLK	0.0917	0.3893	0.6203	0.4490	0.5048	
19	QVTCGTTTL	0.0704	0.2990	0.9775	1.1170	0.5015	

254	SAQTGIAVL	0.0701	0.2975	0.9645	1.0760	0.4960
104	VRIQPGQTF	0.0531	0.2253	0.8613	2.7670	0.4929
197	DTTITVNVL	0.0792	0.3362	0.8423	0.5910	0.4921
49	MLNPNYEDL	0.0737	0.3130	0.9214	0.7880	0.4906
5	KMAFPSGKV	0.0729	0.3094	0.9651	0.6920	0.4887
69	QAGNVQLRV	0.0915	0.3886	0.5614	0.2640	0.4860
223	FTTTLNDFN	0.1278	0.5426	0.0225	-1.3360	0.4792
60	RKSNHNFLV	0.0798	0.3386	0.7271	0.6130	0.4783
67	LVQAGNVQL	0.0676	0.2870	0.9057	0.9600	0.4709
142	NGSCGSVGF	0.0518	0.2201	0.8785	2.3490	0.4693
209	YAAVINGDR	0.0880	0.3735	0.0818	1.5820	0.4648
222	RFTTTLNDF	0.0538	0.2283	0.5532	3.0510	0.4638
44	CTSEDMLNP	0.1073	0.4555	0.0243	0.0470	0.4615
226	TLNDFNLVA	0.0921	0.3910	0.6700	-0.6210	0.4605
164	HMELPTGVH	0.0871	0.3697	0.8244	-0.6720	0.4598
224	TTTLNDFNL	0.0908	0.3855	0.2364	0.7710	0.4595
260	AVLDMCASL	0.0588	0.2496	0.9666	1.2640	0.4578
200	ITVNVLAWL	0.0672	0.2851	0.7940	1.0440	0.4564
128	CAMRPNFTI	0.0687	0.2918	0.9030	0.5620	0.4554
215	GDRWFLNRF	0.0490	0.2080	0.8800	2.2380	0.4519
177	LEGNFYGPF	0.0617	0.2619	0.5531	2.1010	0.4499
95	NPKTPKYKF	0.0434	0.1845	0.9601	2.4190	0.4494
180	NFYGPFVDR	0.0496	0.2105	0.9661	1.8460	0.4477
97	KTPKYKFVR	0.0676	0.2868	0.5741	1.4100	0.4435
58	LIRKSNHNF	0.0641	0.2724	0.2109	2.7640	0.4422
129	AMRPNFTIK	0.0602	0.2557	0.9625	0.7720	0.4386
140	FLNGSCGSV	0.0719	0.3052	0.7902	0.1990	0.4337
106	IQPGQTFSV	0.0609	0.2586	0.9756	0.5300	0.4314
126	YQCAMRPNF	0.0684	0.2903	0.0536	2.6460	0.4307
12	KVEGCMVQV	0.0775	0.3290	0.5447	0.3860	0.4300
239	YEPLTQDHV	0.0695	0.2953	0.7922	0.2850	0.4283
211	AVINGDRWF	0.0593	0.2518	0.1557	3.0400	0.4271
205	LAWLYAAVI	0.0603	0.2559	0.8650	0.8100	0.4262
194	AGTDTTITV	0.0643	0.2730	0.8827	0.3100	0.4209
204	VLAWLYAAV	0.0794	0.3369	0.3587	0.5540	0.4184
234	AMKYNYEPL	0.0542	0.2302	0.8302	1.2650	0.4179
132	PNFTIKGSF	0.0483	0.2051	0.6862	2.1460	0.4154
192	QAAGTDTTI	0.0771	0.3272	0.3248	0.7710	0.4145
290	EFTPFDVVR	0.0479	0.2035	0.9544	1.2730	0.4103
59	IRKSNHNFL	0.0493	0.2093	0.9245	1.2300	0.4095
92	DTANPKTPK	0.0689	0.2925	0.6897	0.1910	0.4056

35	VVYCPRHVI	0.0524	0.2226	0.9571	0.7830	0.4053
198	TTITVNVLA	0.1007	0.4275	0.0571	-0.6480	0.4037
274	NGMNGRTIL	0.0556	0.2359	0.8713	0.7410	0.4036
278	GRTILGSAL	0.0483	0.2050	0.9609	1.0870	0.4034
74	QLRVIGHSM	0.0581	0.2467	0.8998	0.4330	0.4033
212	VINGDRWFL	0.0656	0.2784	0.4296	1.2050	0.4031
163	HHMELPTGV	0.0588	0.2498	0.8911	0.3320	0.4001
107	QPGQTFSVL	0.0536	0.2275	0.9780	0.5090	0.3997
31	WLDDVVYCP	0.0903	0.3832	0.1133	-0.0310	0.3987
159	FCYMHHMEL	0.0648	0.2752	0.3944	1.1210	0.3904
245	DHVDILGPL	0.0491	0.2084	0.9561	0.6750	0.3855
199	TITVNVLAW	0.0647	0.2748	0.4084	0.9660	0.3843
133	NFTIKGSFL	0.0473	0.2007	0.8908	0.9780	0.3832
263	DMCASLKEL	0.0637	0.2706	0.4633	0.8170	0.3810
42	VICTSEDML	0.0696	0.2953	0.1856	1.1310	0.3797
79	GHSMQNCVL	0.0483	0.2051	0.8993	0.7880	0.3794
41	HVICTSEDM	0.0688	0.2920	0.3478	0.5530	0.3718
271	LLQNGMNGR	0.0630	0.2676	0.2531	1.3090	0.3710
264	MCASLKELL	0.0712	0.3023	0.1443	0.9000	0.3689
214	NGDRWFLNR	0.0653	0.2772	0.2389	1.0880	0.3675
53	NYEDLLIRK	0.0555	0.2355	0.7029	0.4770	0.3648
68	VQAGNVQLR	0.0605	0.2568	0.1444	1.6870	0.3629
295	DVVRQCSGV	0.0587	0.2491	0.7132	0.1100	0.3616
50	LNPNYEDLL	0.0608	0.2581	0.4079	0.8270	0.3607
241	PLTQDHVDI	0.0528	0.2242	0.9424	-0.1290	0.3592
98	TPKYKFVRI	0.0469	0.1993	0.9573	0.3080	0.3583
289	DEFTPFDVV	0.0532	0.2258	0.9453	-0.1920	0.3580
251	GPLSAQTGI	0.0522	0.2218	0.8450	0.1170	0.3544
196	TDTTITVNV	0.0584	0.2481	0.7282	-0.0800	0.3534
246	HVDILGPLS	0.1113	0.4726	0.0349	-2.5130	0.3521
210	AAVINGDRW	0.0539	0.2287	0.4492	1.1070	0.3514
114	VLACYNGSP	0.0783	0.3323	0.0333	0.2570	0.3502
169	TGVHAGTDL	0.0506	0.2147	0.6985	0.6000	0.3495
165	MELPTGVHA	0.0570	0.2421	0.9187	-0.6290	0.3485
130	MRPNFTIKG	0.0668	0.2837	0.7644	-1.0030	0.3482
173	AGTDLEGNF	0.0482	0.2048	0.1675	2.3530	0.3475
156	CVSFCYMHH	0.0842	0.3577	0.1177	-0.5810	0.3463
117	CYNGSPSGV	0.0510	0.2167	0.6599	0.5980	0.3456
228	NDFNLVAMK	0.0550	0.2333	0.6255	0.3540	0.3449
233	VAMKYNYEP	0.0750	0.3185	0.0416	0.3320	0.3413
227	LNDFNLVAM	0.0594	0.2523	0.5904	-0.0100	0.3404

61	KSNHNFLVQ	0.0765	0.3246	0.0573	0.0800	0.3372
83	QNCVLKLKV	0.0627	0.2664	0.3735	0.2200	0.3334
268	LKELLQNGM	0.0548	0.2326	0.5735	0.2680	0.3320
265	CASLKELLQ	0.0777	0.3301	0.0293	-0.0620	0.3314
65	NFLVQAGNV	0.0555	0.2358	0.4617	0.5140	0.3307
90	KVDTANPKT	0.0834	0.3542	0.0364	-0.5880	0.3302
52	PNYEDLLIR	0.0479	0.2035	0.4507	1.1800	0.3301
22	CGTTTLNGL	0.0548	0.2327	0.4402	0.6210	0.3297
161	YMHHMELPT	0.0826	0.3506	0.0297	-0.5810	0.3260
82	MQNCVLKLK	0.0631	0.2677	0.1853	0.5470	0.3229
89	LKVDTANPK	0.0559	0.2371	0.3105	0.6970	0.3186
94	ANPKTPKYK	0.0517	0.2197	0.4933	0.4510	0.3162
189	QTAQAAGTD	0.0925	0.3928	0.0233	-1.6750	0.3126
4	RKMAFPSGK	0.0597	0.2533	0.1516	0.7180	0.3120
122	PSGVYQCAM	0.0703	0.2986	0.1694	-0.2440	0.3118
121	SPSGVYQCA	0.0519	0.2204	0.8655	-0.8210	0.3092
20	VTCGTTTLN	0.0842	0.3575	0.1114	-1.3150	0.3084
154	YDCVSFCYM	0.0640	0.2717	0.2884	-0.1530	0.3073
144	SCGSVGFNI	0.0626	0.2656	0.1383	0.4150	0.3071
32	LDDVVYCPR	0.0548	0.2325	0.1237	0.9490	0.2985
134	FTIKGSFLN	0.0829	0.3522	0.0460	-1.2220	0.2980
51	NPNYEDLLI	0.0560	0.2377	0.3186	0.2210	0.2965
291	FTPFDVVRQ	0.0687	0.2917	0.1581	-0.4040	0.2952
183	GPFVDRQTA	0.0468	0.1986	0.9554	-0.9590	0.2940
62	SNHNFLVQA	0.0606	0.2573	0.4079	-0.5040	0.2933
10	SGKVEGCMV	0.0541	0.2297	0.4221	-0.0460	0.2907
298	RQCSGVTFQ	0.0615	0.2610	0.1047	0.2700	0.2902
75	LRVIGHSMQ	0.0490	0.2081	0.4968	0.1340	0.2893
123	SGVYQCAMR	0.0498	0.2115	0.0690	1.2040	0.2820
66	FLVQAGNVQ	0.0643	0.2728	0.0472	0.0380	0.2818
34	DVVYCPRHV	0.0522	0.2217	0.3717	0.0760	0.2812
27	LNGLWLDDV	0.0594	0.2522	0.1852	0.0130	0.2806
252	PLSAQTGIA	0.0627	0.2664	0.4012	-1.0300	0.2751
285	ALLEDEFTP	0.0586	0.2490	0.0566	0.3400	0.2745
45	TSEDMLNPN	0.0803	0.3411	0.0282	-1.4470	0.2730
124	GVYQCAMRP	0.0594	0.2523	0.0355	0.3050	0.2729
96	PKTPKYKFV	0.0438	0.1859	0.5762	-0.0110	0.2718
167	LPTGVHAGT	0.0513	0.2177	0.7573	-1.2090	0.2708
203	NVLAWLYAA	0.0616	0.2617	0.1526	-0.2950	0.2699
86	VLKLKVDTA	0.0554	0.2350	0.3815	-0.4630	0.2691
120	GSPSGVYQC	0.0568	0.2414	0.1800	0.0020	0.2685

288	EDEFTPFDV	0.0513	0.2176	0.4200	-0.2930	0.2660
17	MVQVTCGTT	0.0672	0.2853	0.0501	-0.5590	0.2649
28	NGLWLDDVV	0.0566	0.2401	0.0923	0.1510	0.2615
25	TTLNGLWLD	0.0816	0.3465	0.0297	-1.8300	0.2594
78	IGHSMQNCV	0.0535	0.2273	0.1435	0.1480	0.2562
176	DLEGNFYGP	0.0589	0.2503	0.1471	-0.3260	0.2560
236	KYNYEPLTQ	0.0463	0.1968	0.3186	0.2080	0.2550
149	GFNIDYDCV	0.0451	0.1915	0.3232	0.2560	0.2528
16	CMVQVTCGT	0.0649	0.2754	0.0334	-0.5770	0.2516
218	WFLNRFTTT	0.0564	0.2394	0.2177	-0.4150	0.2513
70	AGNVQLRVI	0.0493	0.2094	0.1847	0.2690	0.2506
243	TQDHVDILG	0.0739	0.3136	0.0647	-1.4690	0.2498
191	AQAAGTDTT	0.0633	0.2689	0.0237	-0.4590	0.2495
235	MKYNYEPLT	0.0600	0.2546	0.0470	-0.2680	0.2483
273	QNGMNGRTI	0.0523	0.2221	0.0767	0.2800	0.2476
277	NGRTILGSA	0.0527	0.2239	0.3810	-0.6800	0.2470
258	GIAVLDMCA	0.0580	0.2462	0.1917	-0.5590	0.2470
181	FYGPFVDRQ	0.0550	0.2337	0.0743	0.0420	0.2469
160	CYMHHMELP	0.0538	0.2286	0.0262	0.2880	0.2469
77	VIGHSMQNC	0.0552	0.2342	0.0228	0.1740	0.2463
178	EGNFYGPFV	0.0520	0.2208	0.2363	-0.2020	0.2461
18	VQVTCGTTT	0.0615	0.2611	0.0462	-0.4540	0.2453
190	TAQAAGTDT	0.0656	0.2786	0.0243	-0.7550	0.2445
108	PGQTFSVLA	0.0562	0.2384	0.4881	-1.3630	0.2435
37	YCPRHVICT	0.0630	0.2677	0.0565	-0.6730	0.2425
9	PSGKVEGCM	0.0574	0.2438	0.1230	-0.4030	0.2421
72	NVQLRVIGH	0.0606	0.2574	0.0333	-0.4220	0.2413
257	TGIAVLDMC	0.0587	0.2492	0.0237	-0.2530	0.2401
272	LQNGMNGRT	0.0633	0.2687	0.0269	-0.6720	0.2391
292	TPFDVVRQC	0.0498	0.2115	0.2813	-0.3040	0.2385
88	KLKVDTANP	0.0506	0.2147	0.0636	0.2770	0.2381
85	CVLKLKVDT	0.0601	0.2551	0.0331	-0.4490	0.2376
1	SGFRKMAFP	0.0549	0.2332	0.0275	0.0010	0.2374
36	VYCPRHVIC	0.0493	0.2092	0.0416	0.3970	0.2353
148	VGFNIDYDC	0.0542	0.2303	0.0253	-0.0240	0.2329
202	VNVLAWLYA	0.0595	0.2528	0.0371	-0.5380	0.2315
275	GMNGRTILG	0.0618	0.2623	0.2592	-1.4060	0.2309
193	AAGTDTTIT	0.0617	0.2619	0.0276	-0.7210	0.2300
284	SALLEDEFT	0.0604	0.2563	0.0245	-0.6120	0.2294
244	QDHVDILGP	0.0542	0.2300	0.0414	-0.1500	0.2287
137	KGSFLNGSC	0.0536	0.2275	0.0300	-0.1400	0.2250

11	GKVEGCMVQ	0.0455	0.1932	0.2424	-0.1030	0.2245
30	LWLDDVVYC	0.0488	0.2070	0.0253	0.2530	0.2235
76	RVIGHSMQN	0.0593	0.2517	0.0792	-0.8160	0.2228
152	IDYDCVSFC	0.0506	0.2147	0.0337	0.0580	0.2227
249	ILGPLSAQT	0.0583	0.2477	0.1454	-0.9650	0.2212
217	RWFLNRFTT	0.0522	0.2216	0.0781	-0.2420	0.2212
109	GQTFSVLAC	0.0529	0.2248	0.0626	-0.2700	0.2207
8	FPSGKVEGC	0.0537	0.2278	0.0300	-0.2350	0.2206
207	WLYAAVING	0.0626	0.2658	0.0294	-1.0620	0.2171
14	EGCMVQVTC	0.0548	0.2326	0.0285	-0.4000	0.2169
102	KFVRIQPGQ	0.0476	0.2019	0.0349	0.1950	0.2169
247	VDILGPLSA	0.0533	0.2262	0.1825	-0.7350	0.2168
281	ILGSALLED	0.0670	0.2843	0.1918	-1.9260	0.2167
296	VVRQCSGVT	0.0549	0.2330	0.0824	-0.5950	0.2156
232	LVAMKYNYE	0.0647	0.2745	0.0261	-1.2650	0.2152
184	PFVDRQTAQ	0.0425	0.1803	0.3517	-0.3700	0.2146
103	FVRIQPGQT	0.0556	0.2359	0.0268	-0.5120	0.2143
266	ASLKELLQN	0.0626	0.2660	0.0272	-1.1200	0.2140
280	TILGSALLE	0.0645	0.2740	0.0276	-1.3080	0.2127
248	DILGPLSAQ	0.0513	0.2180	0.0402	-0.2280	0.2126
100	KYKFVRIQP	0.0449	0.1907	0.0347	0.3290	0.2123
188	RQTAQAAGT	0.0554	0.2354	0.0255	-0.5610	0.2111
127	QCAMRPNFT	0.0571	0.2424	0.0226	-0.7070	0.2104
238	NYEPLTQDH	0.0501	0.2127	0.1207	-0.4300	0.2093
143	GSCGSVGFN	0.0636	0.2701	0.0229	-1.3040	0.2084
155	DCVSFCYMH	0.0541	0.2297	0.1132	-0.8050	0.2065
57	LLIRKSNHN	0.0611	0.2593	0.1002	-1.3620	0.2062
7	AFPSGKVEG	0.0551	0.2341	0.1583	-1.0630	0.2047
91	VDTANPKTP	0.0479	0.2032	0.0261	-0.0760	0.2033
171	VHAGTDLEG	0.0610	0.2590	0.0517	-1.3390	0.1998
182	YGPFVDRQT	0.0577	0.2452	0.0597	-1.1120	0.1985
172	HAGTDLEGN	0.0629	0.2671	0.0235	-1.4700	0.1971
101	YKFVRIQPG	0.0577	0.2450	0.0599	-1.1530	0.1963
99	PKYKFVRIQ	0.0479	0.2032	0.0317	-0.3390	0.1910
135	TIKGSFLNG	0.0554	0.2354	0.1691	-1.3950	0.1910
13	VEGCMVQVT	0.0535	0.2272	0.0339	-0.8410	0.1902
166	ELPTGVHAG	0.0588	0.2497	0.1160	-1.5590	0.1891
26	TLNGLWLDD	0.0656	0.2784	0.0332	-1.9010	0.1883
119	NGSPSGVYQ	0.0470	0.1996	0.0459	-0.3910	0.1870
112	FSVLACYNG	0.0582	0.2471	0.0366	-1.3620	0.1845
87	LKLKVDTAN	0.0562	0.2385	0.0272	-1.1970	0.1827

237	YNYEPLTQD	0.0567	0.2406	0.1788	-1.7000	0.1824
21	TCGTTTLNG	0.0599	0.2544	0.0236	-1.5120	0.1823
56	DLLIRKSNH	0.0515	0.2187	0.0788	-0.9810	0.1815
116	ACYNGSPSG	0.0529	0.2246	0.0434	-0.9960	0.1813
43	ICTSEDMLN	0.0564	0.2396	0.0232	-1.2610	0.1801
216	DRWFLNRFT	0.0478	0.2031	0.0510	-0.6730	0.1771
105	RIQPGQTFS	0.0615	0.2613	0.0430	-1.8580	0.1748
269	KELLQNGMN	0.0549	0.2333	0.0352	-1.2780	0.1747
111	TFSVLACYN	0.0546	0.2318	0.0248	-1.2220	0.1744
267	SLKELLQNG	0.0532	0.2261	0.0444	-1.1710	0.1742
255	AQTGIAVLD	0.0570	0.2422	0.0684	-1.5780	0.1736
206	AWLYAAVIN	0.0502	0.2132	0.0224	-0.8590	0.1736
33	DDVVYCPRH	0.0478	0.2031	0.1436	-1.0330	0.1730
186	VDRQTAQAA	0.0481	0.2042	0.0681	-0.8370	0.1726
73	VQLRVIGHS	0.0618	0.2623	0.0330	-1.9490	0.1698
213	INGDRWFLN	0.0568	0.2413	0.0270	-1.5610	0.1673
187	DRQTAQAAG	0.0552	0.2343	0.0473	-1.4900	0.1669
64	HNFLVQAGN	0.0537	0.2279	0.0265	-1.3060	0.1666
221	NRFTTTLND	0.0547	0.2323	0.0319	-1.4120	0.1665
270	ELLQNGMNG	0.0543	0.2307	0.0469	-1.4610	0.1647
113	SVLACYNGS	0.0627	0.2663	0.0227	-2.1410	0.1627
170	GVHAGTDLE	0.0557	0.2364	0.0257	-1.5660	0.1619
54	YEDLLIRKS	0.0655	0.2783	0.0318	-2.4360	0.1613
138	GSFLNGSCG	0.0520	0.2209	0.0391	-1.3210	0.1608
48	DMLNPNYED	0.0598	0.2538	0.0934	-2.1590	0.1599
158	SFCYMHHME	0.0516	0.2189	0.0230	-1.2890	0.1579
147	SVGFNIDYD	0.0576	0.2444	0.0281	-1.8170	0.1578
3	FRKMAFPSG	0.0505	0.2146	0.0527	-1.3160	0.1567
115	LACYNGSPS	0.0606	0.2574	0.0228	-2.1330	0.1541
250	LGPLSAQTG	0.0537	0.2281	0.0595	-1.6640	0.1539
162	MHHMELPTG	0.0511	0.2171	0.0284	-1.3630	0.1532
125	VYQCAMRPN	0.0471	0.1999	0.0244	-1.0510	0.1510
230	FNLVAMKYN	0.0543	0.2306	0.0248	-1.6840	0.1501
276	MNGRTILGS	0.0637	0.2703	0.0382	-2.5440	0.1488
208	LYAAVINGD	0.0523	0.2220	0.0391	-1.5860	0.1486
262	LDMCASLKE	0.0546	0.2319	0.0273	-1.7570	0.1482
168	PTGVHAGTD	0.0623	0.2647	0.0340	-2.4320	0.1482
175	TDLEGNFYG	0.0528	0.2240	0.0636	-1.7360	0.1468
141	LNGSCGSVG	0.0547	0.2322	0.0238	-1.8120	0.1451
287	LEDEFTPFD	0.0552	0.2344	0.0852	-2.0750	0.1435
6	MAFPSGKVE	0.0476	0.2021	0.0253	-1.2530	0.1433

40	RHVICTSED	0.0506	0.2150	0.0535	-1.6310	0.1415
294	FDVVRQCSG	0.0534	0.2268	0.0473	-1.8650	0.1406
150	FNIDYDCVS	0.0609	0.2587	0.0275	-2.4540	0.1401
220	LNRFTTTLN	0.0493	0.2095	0.0405	-1.5200	0.1395
139	SFLNGSCGS	0.0554	0.2353	0.0320	-2.0370	0.1382
71	GNVQLRVIG	0.0483	0.2049	0.0258	-1.4950	0.1340
15	GCMVQVTCG	0.0499	0.2118	0.0306	-1.6560	0.1336
63	NHNFLVQAG	0.0463	0.1964	0.0270	-1.3910	0.1309
145	CGSVGFNID	0.0541	0.2298	0.0319	-2.0730	0.1309
259	IAVLDMCAS	0.0548	0.2325	0.0246	-2.1220	0.1301
282	LGSALLEDE	0.0507	0.2152	0.0228	-1.8230	0.1275
136	IKGSFLNGS	0.0557	0.2363	0.0375	-2.3260	0.1257
38	CPRHVICTS	0.0504	0.2141	0.2237	-2.5940	0.1180
240	EPLTQDHVD	0.0484	0.2055	0.1785	-2.3580	0.1144
84	NCVLKLKVD	0.0462	0.1962	0.0269	-1.7290	0.1138
2	GFRKMAFPS	0.0512	0.2174	0.0246	-2.1540	0.1134
39	PRHVICTSE	0.0447	0.1899	0.0785	-1.8160	0.1109
131	RPNFTIKGS	0.0466	0.1979	0.1733	-2.3640	0.1057
55	EDLLIRKSN	0.0455	0.1930	0.0250	-1.8610	0.1037
47	EDMLNPNYE	0.0456	0.1935	0.0259	-1.9580	0.0995
179	GNFYGPFVD	0.0437	0.1856	0.0480	-2.2020	0.0827
293	PFDVVRQCS	0.0460	0.1952	0.0282	-2.7240	0.0632

Table S3. List of predicted CTL from SARS-CoV-2 Nsp12 RdRp. → -E represents the MHC ligands and top epitopes.

NetCTL-1.2 predictions using MHC supertype A1. Threshold 0.750000

Number of MHC ligands 40 identified. Number of peptides 924. Protein name Nsp12

Residue No.	Peptide Sequence	Predicted MHC binding affinity	Rescale binding affinity	C-terminal cleavage affinity	Transport efficiency	Prediction score	Identified MHC ligand
738	DTDFVNEFY	0.7922	3.3634	0.8873	2.4580	3.6194	<-E
450	ISDYDYYRY	0.7054	2.9952	0.9586	2.9660	3.2873	<-E
907	LTNDNTSRY	0.6579	2.7931	0.9729	2.8490	3.0815	<-E
475	VVDKYFDCY	0.6001	2.5478	0.9439	2.7820	2.8284	<-E
681	SSGDATTAY	0.5267	2.2363	0.9582	2.8650	2.5232	<-E
895	LTGHMLDMY	0.5234	2.2221	0.1651	2.5770	2.3757	<-E
209	NQDLNGNWY	0.4556	1.9343	0.8075	2.7220	2.1915	<-E
538	TITQMNLKY	0.4248	1.8036	0.9448	2.8510	2.0878	<-E
366	LSFKELLVY	0.3898	1.6552	0.9676	3.2130	1.9610	<-E
27	STDVVYRAF	0.4019	1.7065	0.6174	2.4000	1.9191	<-E
869	LTKHPNQEY	0.3819	1.6213	0.8527	2.7600	1.8872	<-E
859	FVSLAIDAY	0.3709	1.5746	0.7669	3.0960	1.8444	<-E
666	MVMCGGSLY	0.3637	1.5441	0.9482	3.0080	1.8368	<-E
877	YADVFHLYL	0.3906	1.6583	0.9446	0.6440	1.8322	<-E
758	LSDDAVVCF	0.3143	1.3345	0.9556	2.4120	1.5985	<-E
606	YSDVENPHL	0.3278	1.3919	0.8931	0.8460	1.5681	<-E
447	NAAISDYDY	0.3079	1.3074	0.6450	3.0010	1.5542	<-E
686	TTAYANSVF	0.2963	1.2580	0.4772	2.6630	1.4627	<-E
762	AVVCFNSTY	0.2435	1.0339	0.9754	3.1460	1.3375	<-E
463	MCDIRQLLF	0.2518	1.0691	0.1005	2.4360	1.2060	<-E
233	VVDSYYSLL	0.2332	0.9901	0.7134	0.8340	1.1388	<-E
899	MLDMYSVML	0.2090	0.8873	0.9642	0.8220	1.0731	<-E
471	FVVEVVDKY	0.1781	0.7564	0.9738	3.0940	1.0571	<-E
700	VTANVNALL	0.2007	0.8523	0.9705	1.1660	1.0562	<-E
818	MLVKQGDDY	0.1793	0.7614	0.8328	3.0790	1.0403	<-E
823	GDDYVYLPY	0.1821	0.7733	0.8456	2.2130	1.0108	<-E
879	DVFHLYLQY	0.1677	0.7119	0.9529	3.0130	1.0055	<-E
876	EYADVFHLY	0.1624	0.6894	0.9603	2.9530	0.9811	<-E
275	FTEERLKLF	0.1977	0.8396	0.1147	2.1350	0.9635	<-E
388	LLDKRTTCF	0.1638	0.6956	0.9606	2.4300	0.9612	<-E
230	GVPVVDSYY	0.1504	0.6386	0.9521	2.9230	0.9276	<-E
265	YIKWDLLKY	0.1462	0.6206	0.9729	2.8860	0.9108	<-E

434	SVELKHFFF	0.1454	0.6176	0.9285	2.6360	0.8886	<-E
334	FVDGVPFVV	0.1739	0.7382	0.8437	0.1910	0.8743	<-E
645	CCSLSHRFY	0.1586	0.6732	0.2740	2.9100	0.8598	<-E
863	AIDAYPLTK	0.1609	0.6831	0.9559	0.6090	0.8569	<-E
114	ISRQRLTKY	0.1257	0.5338	0.9087	3.0140	0.8208	<-E
381	HAASGNLLL	0.1490	0.6325	0.9069	0.9690	0.8170	<-E
448	AAISDYDYY	0.1233	0.5234	0.6985	3.3310	0.7947	<-E
281	KLFDRYFKY	0.1052	0.4466	0.9756	3.2110	0.7535	<-E
141	TLKEILVTY	0.1082	0.4594	0.9796	2.7820	0.7455	
513	RLYYDSMSY	0.0976	0.4145	0.9722	3.5290	0.7367	
124	MADLVYALR	0.1201	0.5099	0.8435	1.4180	0.7073	
309	HCANFNVLF	0.1147	0.4872	0.5952	2.5290	0.7029	
121	KYTMADLVY	0.0908	0.3856	0.9703	3.2700	0.6947	
642	HTTCCSLSH	0.1673	0.7101	0.0889	-0.5830	0.6943	
802	ETDLTKGPH	0.1542	0.6546	0.4895	-0.9270	0.6817	
30	VVYRAFDIY	0.1002	0.4253	0.6458	3.1730	0.6808	
433	SSVELKHFF	0.1102	0.4680	0.5002	2.6760	0.6769	
768	STYASQGLV	0.1351	0.5735	0.4410	0.6740	0.6734	
61	EDDNLIDSY	0.1046	0.4440	0.8116	2.0980	0.6707	
587	VVIGTSKFY	0.0872	0.3703	0.9221	3.2040	0.6688	
286	YFKYWDQTY	0.0840	0.3565	0.8606	3.2020	0.6457	
685	ATTAYANSV	0.1324	0.5623	0.3838	0.2620	0.6330	
611	NPHLMGWDY	0.0849	0.3606	0.9693	2.5200	0.6320	
913	SRYWEPEFY	0.0745	0.3161	0.9357	3.3810	0.6255	
780	KNFKSVLYY	0.0795	0.3375	0.8665	3.0000	0.6175	
779	IKNFKSVLY	0.0782	0.3321	0.8730	2.9570	0.6109	
79	NYQHEETIY	0.0708	0.3006	0.9580	3.2480	0.6067	
566	MTNRQFHQK	0.1040	0.4416	0.9300	0.4060	0.6014	
65	LIDSYFVVK	0.1029	0.4370	0.8711	0.5620	0.5957	
720	VRNLQHRLY	0.0710	0.3016	0.9424	3.0470	0.5954	
590	GTSKFYGGW	0.1080	0.4584	0.5630	0.6890	0.5773	
148	TYNCCDDDY	0.0844	0.3585	0.3831	3.1210	0.5720	
258	DTDLTKPYI	0.1180	0.5008	0.4488	0.0680	0.5716	
399	AALTNNVAF	0.0696	0.2953	0.8950	2.7960	0.5694	
360	NLHSSRLSF	0.0762	0.3236	0.7322	2.6680	0.5668	
340	FVVSTGYHF	0.0785	0.3333	0.7174	2.5110	0.5665	
563	CSTMTNRQF	0.0946	0.4015	0.2593	2.5070	0.5657	
741	FVNEFYAYL	0.0926	0.3933	0.8931	0.7650	0.5655	
48	FLKTNCCRF	0.0684	0.2904	0.9745	2.5580	0.5644	
123	TMADLVYAL	0.0865	0.3673	0.9660	1.0200	0.5632	
301	CLDDRCILH	0.1318	0.5596	0.1936	-0.5920	0.5590	

520	SYEDQDALF	0.0659	0.2798	0.9277	2.7440	0.5562
804	DLTKGPHEF	0.0689	0.2927	0.9762	2.2120	0.5497
586	TVVIGTSKF	0.0623	0.2644	0.9378	2.8840	0.5493
365	RLSFKELLV	0.0889	0.3776	0.9309	0.6280	0.5486
508	KWGKARLYY	0.0663	0.2815	0.6897	3.2560	0.5478
472	VVEVVDKYF	0.0699	0.2966	0.8180	2.4860	0.5436
432	GSSVELKHF	0.0688	0.2920	0.9278	2.2250	0.5425
278	ERLKLFDRY	0.0618	0.2626	0.9403	2.7780	0.5425
229	SGVPVVDSY	0.0623	0.2647	0.9195	2.7590	0.5406
820	VKQGDDYVY	0.0574	0.2437	0.9491	3.0520	0.5386
667	VMCGGSLYV	0.0870	0.3694	0.9359	0.5430	0.5370
715	IADKYVRNL	0.0823	0.3496	0.9230	0.9550	0.5358
740	DFVNEFYAY	0.0581	0.2465	0.9612	2.8640	0.5339
241	LMPILTLTR	0.0711	0.3018	0.9707	1.6430	0.5296
785	VLYYQNNVF	0.0639	0.2712	0.7634	2.8570	0.5286
724	QHRLYECLY	0.0563	0.2392	0.8990	3.0420	0.5261
184	QALLKTVQF	0.0594	0.2524	0.9021	2.7500	0.5252
813	CSQHTMLVK	0.1094	0.4647	0.2931	0.3270	0.5250
397	SVAALTNNV	0.0928	0.3942	0.7128	0.4180	0.5220
338	VPFVVSTGY	0.0557	0.2366	0.9787	2.7640	0.5216
719	YVRNLQHRL	0.0743	0.3156	0.9559	1.2230	0.5202
564	STMTNRQFH	0.1258	0.5342	0.0466	-0.4460	0.5189
318	STVFPPTSF	0.0644	0.2735	0.7892	2.5390	0.5188
82	HEETIYNLL	0.0838	0.3556	0.9079	0.5350	0.5186
598	WHNMLKTVY	0.0633	0.2686	0.7395	2.7810	0.5186
375	AADPAMHAA	0.1047	0.4445	0.6792	-0.5670	0.5181
507	NKWGKARLY	0.0570	0.2418	0.8004	3.0780	0.5158
69	YFVVKRHTF	0.0563	0.2392	0.8967	2.8140	0.5144
917	EPEFYEAMY	0.0619	0.2626	0.9330	2.2160	0.5134
601	MLKTVYSDV	0.0803	0.3409	0.9567	0.5200	0.5104
647	SLSHRFYRL	0.0739	0.3137	0.9642	1.0160	0.5092
24	TGTSTDVVY	0.0617	0.2619	0.7862	2.5710	0.5084
379	AMHAASGNL	0.0726	0.3083	0.9593	1.1050	0.5074
72	VKRHTFSNY	0.0517	0.2195	0.8739	3.1320	0.5072
155	DYFNKKDWY	0.0534	0.2269	0.9142	2.8460	0.5063
861	SLAIDAYPL	0.0716	0.3038	0.9483	1.0910	0.5006
239	SLLMPILTL	0.0717	0.3043	0.9767	0.9930	0.5005
211	DLNGNWYDF	0.0625	0.2652	0.8554	2.0950	0.4983
313	FNVLFSTVF	0.0598	0.2541	0.7913	2.5060	0.4981
445	DGNAAISDY	0.0655	0.2779	0.7259	2.1990	0.4967
812	FCSQHTMLV	0.1007	0.4277	0.4321	0.0690	0.4960

748	YLRKHFSMM	0.0773	0.3280	0.9490	0.5070	0.4957
633	MASLVLARK	0.0759	0.3222	0.9620	0.5690	0.4949
624	RAMPNMLRI	0.0808	0.3429	0.7600	0.7570	0.4947
887	YIRKLHDEL	0.0760	0.3227	0.7832	1.0630	0.4934
96	VAKHDFFKF	0.0483	0.2051	0.9709	2.8010	0.4908
854	LMIERFVSL	0.0671	0.2849	0.9747	1.1590	0.4891
613	HLMGWDYPK	0.0799	0.3391	0.8954	0.3130	0.4891
245	LTLTRALTA	0.0962	0.4085	0.7075	-0.5260	0.4883
343	STGYHFREL	0.0748	0.3174	0.8465	0.8700	0.4879
167	ENPDILRVY	0.0517	0.2194	0.9189	2.6000	0.4872
383	ASGNLLLDK	0.0831	0.3529	0.6739	0.6400	0.4860
711	DGNKIADKY	0.0633	0.2688	0.6950	2.2580	0.4859
923	AMYTPHTVL	0.0650	0.2758	0.9790	1.2400	0.4847
699	AVTANVNAL	0.0665	0.2823	0.9598	1.1520	0.4839
429	FKEGSSVEL	0.0745	0.3164	0.8955	0.6600	0.4837
628	NMLRIMASL	0.0679	0.2882	0.9622	0.9860	0.4818
10	RVCGVSAAR	0.0607	0.2579	0.8170	2.0170	0.4813
898	HMLDMYSVM	0.0778	0.3303	0.8824	0.3230	0.4788
737	VDTDFVNEF	0.0554	0.2352	0.9128	2.1140	0.4778
279	RLKLFDRYF	0.0533	0.2265	0.7846	2.6290	0.4756
522	EDQDALFAY	0.0576	0.2446	0.7620	2.3290	0.4753
906	MLTNDNTSR	0.0647	0.2747	0.8098	1.5620	0.4743
353	VVHNQDVNL	0.0641	0.2720	0.9620	1.1550	0.4741
393	TTCFSVAAL	0.0883	0.3748	0.3103	1.0530	0.4740
307	ILHCANFNV	0.0734	0.3115	0.9487	0.3800	0.4728
882	HLYLQYIRK	0.0708	0.3006	0.9643	0.4750	0.4690
257	VDTDLTKPY	0.0501	0.2128	0.8244	2.6200	0.4675
249	RALTAESHV	0.0699	0.2970	0.9232	0.6390	0.4674
1	SADAQSFLN	0.1231	0.5225	0.0802	-1.3880	0.4652
237	YYSLLMPIL	0.0649	0.2756	0.8439	1.2490	0.4647
414	NFNKDFYDF	0.0486	0.2064	0.8468	2.6150	0.4642
364	SRLSFKELL	0.0627	0.2662	0.8847	1.2860	0.4633
113	HISRQRLTK	0.0852	0.3616	0.5544	0.3530	0.4624
199	AGIVGVLTL	0.0623	0.2643	0.9673	1.0420	0.4615
630	LRIMASLVL	0.0593	0.2519	0.9687	1.2700	0.4607
23	GTGTSTDVV	0.0931	0.3951	0.4539	-0.0560	0.4604
126	DLVYALRHF	0.0511	0.2171	0.7940	2.4700	0.4597
37	IYNDKVAGF	0.0455	0.1931	0.9098	2.5890	0.4590
64	NLIDSYFVV	0.0710	0.3014	0.9463	0.3010	0.4584
632	IMASLVLAR	0.0775	0.3289	0.2679	1.7780	0.4580
341	VVSTGYHFR	0.0721	0.3061	0.4640	1.5760	0.4545

196	MRNAGIVGV	0.0663	0.2817	0.9398	0.6280	0.4540
794	MSEAKCWTE	0.1262	0.5359	0.0266	-1.7270	0.4535
3	DAQSFLNRV	0.0777	0.3298	0.7659	0.1730	0.4533
500	KSAGFPFNK	0.0800	0.3395	0.5950	0.4900	0.4532
77	FSNYQHEET	0.1157	0.4911	0.0246	-0.8440	0.4526
449	AISDYDYYR	0.0669	0.2839	0.4895	1.9070	0.4526
358	DVNLHSSRL	0.0650	0.2762	0.9635	0.6340	0.4524
332	KIFVDGVPF	0.0507	0.2154	0.5755	3.0140	0.4524
640	RKHTTCCSL	0.0597	0.2537	0.8410	1.4320	0.4514
585	ATVVIGTSK	0.0699	0.2967	0.8169	0.6350	0.4510
119	LTKYTMADL	0.0779	0.3306	0.4421	1.0670	0.4503
912	TSRYWEPEF	0.0621	0.2636	0.3290	2.7440	0.4502
594	FYGGWHNML	0.0593	0.2517	0.9622	1.0620	0.4492
692	SVFNICQAV	0.0661	0.2807	0.8692	0.7590	0.4490
197	RNAGIVGVL	0.0594	0.2523	0.9302	1.0990	0.4468
707	LLSTDGNKI	0.0757	0.3215	0.5986	0.7080	0.4467
374	YAADPAMHA	0.0866	0.3677	0.6707	-0.4390	0.4463
835	SRILGAGCF	0.0584	0.2481	0.4124	2.7180	0.4459
848	VKTDGTLMI	0.0664	0.2817	0.8857	0.6190	0.4455
629	MLRIMASLV	0.0810	0.3440	0.4853	0.5710	0.4454
644	TCCSLSHRF	0.0678	0.2879	0.1975	2.5530	0.4451
492	QVIVNNLDK	0.0762	0.3234	0.5329	0.8260	0.4447
204	VLTLDNQDL	0.0617	0.2621	0.8914	0.9700	0.4443
420	YDFAVSKGF	0.0547	0.2323	0.6743	2.2140	0.4442
25	GTSTDVVYR	0.0724	0.3073	0.4793	1.2900	0.4437
462	TMCDIRQLL	0.0802	0.3404	0.3799	0.9190	0.4434
519	MSYEDQDAL	0.0699	0.2968	0.5488	1.2830	0.4433
534	NVIPTITQM	0.0623	0.2645	0.9611	0.6810	0.4427
575	LLKSIAATR	0.0596	0.2529	0.7149	1.6070	0.4405
875	QEYADVFHL	0.0571	0.2426	0.9255	1.1600	0.4394
165	FVENPDILR	0.0796	0.3380	0.2478	1.2450	0.4375
411	KPGNFNKDF	0.0495	0.2100	0.7719	2.2210	0.4369
654	RLANECAQV	0.0723	0.3071	0.6747	0.5430	0.4355
40	DKVAGFAKF	0.0507	0.2154	0.6375	2.4870	0.4354
407	FQTVKPGNF	0.0637	0.2705	0.2131	2.6330	0.4341
294	YHPNCVNCL	0.0602	0.2554	0.8737	0.9370	0.4333
323	PTSFGPLVR	0.0620	0.2634	0.8537	0.8340	0.4332
41	KVAGFAKFL	0.0660	0.2803	0.6586	1.0780	0.4330
581	ATRGATVVI	0.0586	0.2487	0.9347	0.8600	0.4319
537	PTITQMNLK	0.0976	0.4143	0.1013	0.0410	0.4315
552	NRARTVAGV	0.0607	0.2576	0.9303	0.6760	0.4309

363	SSRLSFKEL	0.0579	0.2458	0.8733	1.0740	0.4305
152	CDDDYFNKK	0.0755	0.3207	0.7520	-0.0670	0.4302
774	GLVASIKNF	0.0523	0.2221	0.5812	2.4040	0.4295
416	NKDFYDFAV	0.0828	0.3514	0.4804	0.1200	0.4294
83	EETIYNLLK	0.0700	0.2971	0.8579	0.0660	0.4291
665	EMVMCGGSL	0.0635	0.2696	0.7215	1.0040	0.4280
580	AATRGATVV	0.0644	0.2734	0.8679	0.4840	0.4278
225	TTPGSGVPV	0.0792	0.3363	0.5359	0.2080	0.4271
93	CPAVAKHDF	0.0506	0.2147	0.7256	2.0670	0.4269
188	KTVQFCDAM	0.0751	0.3187	0.5425	0.5160	0.4259
778	SIKNFKSVL	0.0598	0.2539	0.7147	1.2880	0.4255
855	MIERFVSLA	0.1020	0.4330	0.1008	-0.4560	0.4254
756	MILSDDAVV	0.0657	0.2791	0.7824	0.5440	0.4237
321	FPPTSFGPL	0.0611	0.2592	0.8924	0.5870	0.4224
643	TTCCSLSHR	0.0787	0.3342	0.1061	1.4280	0.4215
554	ARTVAGVSI	0.0562	0.2388	0.8894	0.9810	0.4212
883	LYLQYIRKL	0.0509	0.2161	0.9588	1.2180	0.4209
821	KQGDDYVYL	0.0548	0.2325	0.9149	1.0210	0.4208
34	AFDIYNDKV	0.0765	0.3246	0.4457	0.5820	0.4206
496	NNLDKSAGF	0.0504	0.2141	0.5453	2.4840	0.4201
263	KPYIKWDLL	0.0538	0.2282	0.9561	0.9540	0.4193
725	HRLYECLYR	0.0572	0.2429	0.6510	1.5690	0.4190
846	DIVKTDGTL	0.0646	0.2743	0.7272	0.7060	0.4187
849	KTDGTLMIE	0.1160	0.4925	0.0395	-1.6090	0.4180
166	VENPDILRV	0.0651	0.2764	0.9304	0.0420	0.4180
173	RVYANLGER	0.0659	0.2799	0.2075	2.1020	0.4161
33	RAFDIYNDK	0.0640	0.2716	0.6872	0.8210	0.4157
271	LKYDFTEER	0.0603	0.2559	0.4723	1.7730	0.4154
94	PAVAKHDFF	0.0597	0.2536	0.3522	2.1780	0.4153
501	SAGFPFNKW	0.0536	0.2275	0.9422	0.9280	0.4153
467	RQLLFVVEV	0.0673	0.2859	0.6391	0.6610	0.4148
312	NFNVLFSTV	0.0581	0.2468	0.9265	0.5790	0.4147
811	EFCSQHTML	0.0558	0.2367	0.8827	0.9040	0.4143
468	QLLFVVEVV	0.0640	0.2719	0.7826	0.4970	0.4141
215	NWYDFGDFI	0.0547	0.2324	0.8858	0.9750	0.4140
579	IAATRGATV	0.0817	0.3470	0.2874	0.4610	0.4132
401	LTNNVAFQT	0.1033	0.4385	0.0475	-0.6630	0.4125
561	SICSTMTNR	0.0675	0.2867	0.2595	1.7220	0.4117
418	DFYDFAVSK	0.0557	0.2363	0.9672	0.6040	0.4116
170	DILRVYANL	0.0571	0.2426	0.9102	0.6400	0.4111
253	AESHVDTDL	0.0555	0.2358	0.8447	0.9690	0.4109

98	KHDFFKFRI	0.0558	0.2371	0.9442	0.6270	0.4101
81	QHEETIYNL	0.0532	0.2258	0.9656	0.7790	0.4096
797	AKCWTETDL	0.0595	0.2528	0.7145	0.9900	0.4095
6	SFLNRVCGV	0.0596	0.2528	0.8761	0.5040	0.4095
922	EAMYTPHTV	0.0650	0.2758	0.8276	0.1760	0.4087
490	ANQVIVNNL	0.0542	0.2300	0.8391	1.0550	0.4086
157	FNKKDWYDF	0.0519	0.2202	0.5094	2.2330	0.4083
272	KYDFTEERL	0.0497	0.2112	0.9145	1.1980	0.4082
214	GNWYDFGDF	0.0482	0.2046	0.5144	2.5160	0.4075
409	TVKPGNFNK	0.0565	0.2397	0.9392	0.5310	0.4071
95	AVAKHDFFK	0.0800	0.3396	0.2427	0.6190	0.4070
372	LVYAADPAM	0.0613	0.2602	0.7496	0.6820	0.4068
149	YNCCDDDYF	0.0650	0.2759	0.0742	2.3810	0.4061
819	LVKQGDDYV	0.0598	0.2539	0.8496	0.4910	0.4059
47	KFLKTNCCR	0.0517	0.2194	0.5970	1.9310	0.4055
422	FAVSKGFFK	0.0712	0.3022	0.5648	0.3610	0.4050
251	LTAESHVDT	0.0989	0.4198	0.0815	-0.5520	0.4044
767	NSTYASQGL	0.0726	0.3084	0.3414	0.8910	0.4042
260	DLTKPYIKW	0.0543	0.2304	0.9699	0.5620	0.4039
497	NLDKSAGFP	0.0927	0.3937	0.0249	0.1260	0.4038
894	ELTGHMLDM	0.0753	0.3198	0.5771	-0.0550	0.4036
182	VRQALLKTV	0.0568	0.2412	0.8517	0.6830	0.4031
308	LHCANFNVL	0.0528	0.2241	0.8601	0.9840	0.4023
324	TSFGPLVRK	0.0578	0.2456	0.8832	0.4510	0.4006
569	RQFHQKLLK	0.0583	0.2477	0.6669	1.0370	0.3996
593	KFYGGWHNM	0.0522	0.2216	0.9078	0.8200	0.3988
174	VYANLGERV	0.0620	0.2632	0.6384	0.7900	0.3985
880	VFHLYLQYI	0.0548	0.2327	0.8829	0.6550	0.3979
567	TNRQFHQKL	0.0531	0.2253	0.8624	0.8660	0.3979
828	YLPYPDPSR	0.0597	0.2533	0.4569	1.5160	0.3977
380	MHAASGNLL	0.0679	0.2885	0.3264	1.1800	0.3964
786	LYYQNNVFM	0.0518	0.2199	0.9025	0.8070	0.3957
38	YNDKVAGFA	0.1015	0.4310	0.0564	-0.8750	0.3957
232	PVVDSYYSL	0.0511	0.2171	0.9414	0.7380	0.3952
282	LFDRYFKYW	0.0504	0.2139	0.8704	0.9810	0.3935
111	VPHISRQRL	0.0515	0.2187	0.8965	0.7780	0.3921
810	HEFCSQHTM	0.0576	0.2447	0.9654	0.0450	0.3918
487	CINANQVIV	0.0747	0.3170	0.3986	0.2780	0.3907
470	LFVVEVVDK	0.0538	0.2286	0.8192	0.7620	0.3896
733	RNRDVDTDF	0.0515	0.2188	0.2606	2.6230	0.3891
747	AYLRKHFSM	0.0494	0.2100	0.9665	0.6790	0.3889

427	GFFKEGSSV	0.0526	0.2232	0.9316	0.5160	0.3888
549	SAKNRARTV	0.0617	0.2620	0.6985	0.4270	0.3881
506	FNKWGKARL	0.0540	0.2291	0.8735	0.5600	0.3881
179	GERVRQALL	0.0499	0.2119	0.9242	0.7370	0.3874
723	LQHRLYECL	0.0547	0.2322	0.6258	1.2050	0.3864
688	AYANSVFNI	0.0563	0.2390	0.6462	0.9700	0.3844
267	KWDLLKYDF	0.0501	0.2125	0.2315	2.7210	0.3833
784	SVLYYQNNV	0.0728	0.3092	0.2841	0.6250	0.3830
659	CAQVLSEMV	0.0780	0.3312	0.3007	0.1270	0.3827
655	LANECAQVL	0.0627	0.2664	0.4199	1.0490	0.3819
498	LDKSAGFPF	0.0456	0.1935	0.4497	2.3700	0.3795
743	NEFYAYLRK	0.0578	0.2455	0.7165	0.5250	0.3793
749	LRKHFSMMI	0.0506	0.2150	0.8648	0.6910	0.3792
543	NLKYAISAK	0.0583	0.2474	0.6893	0.5550	0.3786
558	AGVSICSTM	0.0544	0.2310	0.8804	0.3070	0.3784
772	SQGLVASIK	0.0659	0.2798	0.4600	0.5840	0.3780
755	MMILSDDAV	0.0787	0.3342	0.0869	0.6060	0.3776
595	YGGWHNMLK	0.0790	0.3353	0.3064	-0.0900	0.3768
256	HVDTDLTKP	0.0844	0.3584	0.1041	0.0300	0.3755
680	TSSGDATTA	0.0763	0.3242	0.5252	-0.5500	0.3754
536	IPTITQMNL	0.0470	0.1996	0.9422	0.6840	0.3751
11	VCGVSAARL	0.0595	0.2528	0.4754	1.0060	0.3744
892	HDELTGHML	0.0612	0.2599	0.6047	0.4600	0.3736
709	STDGNKIAD	0.1090	0.4628	0.0404	-1.9120	0.3732
122	YTMADLVYA	0.0816	0.3463	0.3135	-0.4110	0.3728
236	SYYSLLMPI	0.0545	0.2316	0.6359	0.9000	0.3720
799	CWTETDLTK	0.0608	0.2582	0.5126	0.7350	0.3719
775	LVASIKNFK	0.0776	0.3293	0.0820	0.5940	0.3713
305	RCILHCANF	0.0529	0.2246	0.0672	2.7290	0.3711
689	YANSVFNIC	0.0846	0.3593	0.0338	0.1070	0.3698
829	LPYPDPSRI	0.0487	0.2070	0.9581	0.3690	0.3691
511	KARLYYDSM	0.0555	0.2355	0.7273	0.4830	0.3687
521	YEDQDALFA	0.0925	0.3926	0.1061	-0.8130	0.3679
856	IERFVSLAI	0.0517	0.2195	0.8189	0.5070	0.3677
750	RKHFSMMIL	0.0573	0.2431	0.3687	1.3260	0.3647
255	SHVDTDLTK	0.0592	0.2515	0.6095	0.4360	0.3647
357	QDVNLHSSR	0.0525	0.2230	0.4665	1.4090	0.3634
524	QDALFAYTK	0.0632	0.2682	0.6157	0.0480	0.3629
51	TNCCRFQEK	0.0615	0.2612	0.5428	0.4010	0.3627
442	FAQDGNAAI	0.0757	0.3216	0.1509	0.3550	0.3620
459	NLPTMCDIR	0.0651	0.2763	0.0793	1.4740	0.3619

42	VAGFAKFLK	0.0658	0.2794	0.3762	0.5170	0.3617
915	YWEPEFYEA	0.0666	0.2826	0.6272	-0.3210	0.3606
897	GHMLDMYSV	0.0510	0.2165	0.8769	0.2180	0.3589
180	ERVRQALLK	0.0620	0.2634	0.4567	0.5150	0.3577
830	PYPDPSRIL	0.0411	0.1745	0.9623	0.7720	0.3575
151	CCDDDYFNK	0.0724	0.3072	0.1985	0.4090	0.3574
175	YANLGERVR	0.0569	0.2414	0.2828	1.4700	0.3573
847	IVKTDGTLM	0.0608	0.2581	0.5497	0.3250	0.3568
609	VENPHLMGW	0.0451	0.1916	0.9000	0.5780	0.3555
660	AQVLSEMVM	0.0522	0.2215	0.6933	0.5950	0.3552
646	CSLSHRFYR	0.0630	0.2676	0.0851	1.4950	0.3551
392	RTTCFSVAA	0.0799	0.3393	0.1809	-0.2480	0.3540
872	HPNQEYADV	0.0603	0.2561	0.6756	-0.0740	0.3537
66	IDSYFVVKR	0.0519	0.2203	0.4726	1.2440	0.3534
108	GDMVPHISR	0.0486	0.2064	0.6707	0.9240	0.3532
421	DFAVSKGFF	0.0508	0.2157	0.1285	2.3510	0.3526
138	NCDTLKEIL	0.0711	0.3017	0.0701	0.8060	0.3525
262	TKPYIKWDL	0.0567	0.2407	0.4056	1.0160	0.3523
243	PILTLTRAL	0.0544	0.2311	0.5802	0.6570	0.3509
26	TSTDVVYRA	0.0757	0.3212	0.3901	-0.5820	0.3507
504	FPFNKWGKA	0.0598	0.2538	0.9269	-0.8530	0.3502
88	NLLKDCPAV	0.0602	0.2556	0.5130	0.3370	0.3494
189	TVQFCDAMR	0.0603	0.2561	0.0596	1.6710	0.3486
9	NRVCGVSAA	0.0514	0.2183	0.9145	-0.1660	0.3472
242	MPILTLTRA	0.0586	0.2488	0.8812	-0.6760	0.3471
924	MYTPHTVLQ	0.0542	0.2301	0.6712	0.3240	0.3469
110	MVPHISRQR	0.0543	0.2305	0.2706	1.5160	0.3469
238	YSLLMPILT	0.0892	0.3787	0.0266	-0.7190	0.3467
333	IFVDGVPFV	0.0502	0.2130	0.6555	0.6840	0.3455
850	TDGTLMIER	0.0649	0.2754	0.1019	1.0940	0.3454
597	GWHNMLKTV	0.0525	0.2229	0.6866	0.3820	0.3450
226	TPGSGVPVV	0.0551	0.2339	0.7936	-0.1640	0.3447
325	SFGPLVRKI	0.0518	0.2200	0.6301	0.6020	0.3446
706	ALLSTDGNK	0.0658	0.2796	0.1689	0.7870	0.3443
745	FYAYLRKHF	0.0473	0.2008	0.1009	2.5540	0.3437
698	QAVTANVNA	0.0674	0.2862	0.5146	-0.3990	0.3434
771	ASQGLVASI	0.0642	0.2725	0.2590	0.6350	0.3431
777	ASIKNFKSV	0.0609	0.2588	0.3557	0.6150	0.3429
264	PYIKWDLLK	0.0578	0.2452	0.5104	0.4190	0.3427
851	DGTLMIERF	0.0519	0.2205	0.1674	1.9260	0.3419
346	YHFRELGVV	0.0609	0.2587	0.4240	0.3840	0.3415

390	DKRTTCFSV	0.0547	0.2322	0.6736	0.1450	0.3405
836	RILGAGCFV	0.0663	0.2816	0.1367	0.7590	0.3400
607	SDVENPHLM	0.0530	0.2251	0.7398	0.0760	0.3399
274	DFTEERLKL	0.0533	0.2265	0.5154	0.7180	0.3397
908	TNDNTSRYW	0.0558	0.2369	0.4896	0.5830	0.3395
120	TKYTMADLV	0.0697	0.2961	0.0946	0.5780	0.3392
326	FGPLVRKIF	0.0513	0.2176	0.0722	2.2120	0.3391
452	DYDYYRYNL	0.0521	0.2211	0.5230	0.7810	0.3386
206	TLDNQDLNG	0.0953	0.4048	0.0551	-1.5250	0.3368
734	NRDVDTDFV	0.0674	0.2860	0.1620	0.5140	0.3360
547	AISAKNRAR	0.0567	0.2407	0.0682	1.7010	0.3359
288	KYWDQTYHP	0.0465	0.1972	0.7423	0.5430	0.3357
458	YNLPTMCDI	0.0681	0.2891	0.1308	0.5120	0.3343
402	TNNVAFQTV	0.0618	0.2626	0.4417	0.0950	0.3336
105	RIDGDMVPH	0.0772	0.3277	0.1840	-0.4440	0.3331
916	WEPEFYEAM	0.0581	0.2467	0.5500	0.0750	0.3330
525	DALFAYTKR	0.0541	0.2297	0.2889	1.1900	0.3326
412	PGNFNKDFY	0.0472	0.2006	0.1452	2.1850	0.3316
881	FHLYLQYIR	0.0495	0.2103	0.3186	1.4620	0.3312
456	YRYNLPTMC	0.0654	0.2777	0.2385	0.3360	0.3303
78	SNYQHEETI	0.0549	0.2330	0.4758	0.5170	0.3302
291	DQTYHPNCV	0.0520	0.2209	0.7286	-0.0180	0.3293
568	NRQFHQKLL	0.0484	0.2057	0.4287	1.1760	0.3288
164	DFVENPDIL	0.0465	0.1975	0.5956	0.8320	0.3284
565	TMTNRQFHQ	0.0707	0.3003	0.1780	0.0130	0.3276
571	FHQKLLKSI	0.0528	0.2241	0.5629	0.3660	0.3268
97	AKHDFFKFR	0.0509	0.2161	0.1458	1.7550	0.3257
623	DRAMPNMLR	0.0545	0.2316	0.1647	1.3850	0.3255
696	ICQAVTANV	0.0586	0.2486	0.3550	0.4670	0.3252
742	VNEFYAYLR	0.0601	0.2553	0.0831	1.1450	0.3251
479	YFDCYDGGC	0.0746	0.3168	0.0231	0.0870	0.3246
134	FDEGNCDTL	0.0528	0.2240	0.5371	0.3850	0.3238
793	FMSEAKCWT	0.0807	0.3427	0.0852	-0.6360	0.3237
345	GYHFRELGV	0.0479	0.2036	0.6526	0.4400	0.3235
763	VVCFNSTYA	0.0614	0.2606	0.4752	-0.1720	0.3233
718	KYVRNLQHR	0.0469	0.1991	0.2276	1.7950	0.3230
648	LSHRFYRLA	0.0742	0.3151	0.1886	-0.4070	0.3230
530	YTKRNVIPT	0.0818	0.3472	0.0350	-0.5950	0.3227
890	KLHDELTGH	0.0615	0.2612	0.4881	-0.2470	0.3221
637	VLARKHTTC	0.0686	0.2914	0.1510	0.1610	0.3221
73	KRHTFSNYQ	0.0595	0.2528	0.3629	0.2850	0.3214

873	PNQEYADVF	0.0487	0.2070	0.1463	1.8470	0.3213
625	AMPNMLRIM	0.0634	0.2693	0.1156	0.6870	0.3210
546	YAISAKNRA	0.0758	0.3220	0.1041	-0.3330	0.3210
2	ADAQSFLNR	0.0557	0.2365	0.0996	1.3920	0.3210
658	ECAQVLSEM	0.0604	0.2563	0.3330	0.2790	0.3202
822	QGDDYVYLP	0.0741	0.3147	0.1036	-0.2240	0.3191
591	TSKFYGGWH	0.0804	0.3413	0.0297	-0.5480	0.3184
104	FRIDGDMVP	0.0581	0.2467	0.3405	0.3860	0.3171
528	FAYTKRNVI	0.0631	0.2678	0.0840	0.7270	0.3168
234	VDSYYSLLM	0.0683	0.2899	0.1443	0.1020	0.3167
322	PPTSFGPLV	0.0542	0.2299	0.7802	-0.6110	0.3164
668	MCGGSLYVK	0.0579	0.2457	0.3318	0.4160	0.3163
62	DDNLIDSYF	0.0473	0.2009	0.2198	1.6500	0.3163
13	GVSAARLTP	0.0676	0.2868	0.1711	0.0530	0.3152
109	DMVPHISRQ	0.0606	0.2573	0.4635	-0.2520	0.3142
911	NTSRYWEPE	0.0931	0.3951	0.0240	-1.6930	0.3141
194	DAMRNAGIV	0.0693	0.2944	0.0900	0.1090	0.3134
90	LKDCPAVAK	0.0627	0.2660	0.1856	0.3900	0.3133
106	IDGDMVPHI	0.0516	0.2192	0.5476	0.2190	0.3123
710	TDGNKIADK	0.0585	0.2483	0.4161	0.0270	0.3121
891	LHDELTGHM	0.0602	0.2558	0.2946	0.2410	0.3120
178	LGERVRQAL	0.0558	0.2368	0.2699	0.6890	0.3118
398	VAALTNNVA	0.0676	0.2871	0.2692	-0.3500	0.3100
713	NKIADKYVR	0.0481	0.2042	0.1647	1.6100	0.3094
297	NCVNCLDDR	0.0538	0.2285	0.0474	1.4750	0.3094
351	LGVVHNQDV	0.0576	0.2446	0.4367	-0.0370	0.3083
461	PTMCDIRQL	0.0574	0.2435	0.2411	0.5660	0.3080
430	KEGSSVELK	0.0622	0.2643	0.2343	0.1680	0.3078
531	TKRNVIPTI	0.0559	0.2373	0.2826	0.5600	0.3077
852	GTLMIERFV	0.0601	0.2553	0.3441	-0.0070	0.3066
216	WYDFGDFIQ	0.0619	0.2629	0.2499	0.0970	0.3052
240	LLMPILTLT	0.0703	0.2984	0.2324	-0.5630	0.3051
391	KRTTCFSVA	0.0556	0.2362	0.5910	-0.3970	0.3050
277	EERLKLFDR	0.0485	0.2060	0.2565	1.2040	0.3047
540	TQMNLKYAI	0.0587	0.2492	0.1452	0.6630	0.3041
22	CGTGTSTDV	0.0596	0.2529	0.3953	-0.1740	0.3035
555	RTVAGVSIC	0.0649	0.2756	0.0278	0.4690	0.3032
545	KYAISAKNR	0.0440	0.1868	0.1315	1.9210	0.3026
503	GFPFNKWGK	0.0489	0.2077	0.4334	0.5920	0.3023
29	DVVYRAFDI	0.0580	0.2461	0.2262	0.4450	0.3022
455	YYRYNLPTM	0.0497	0.2109	0.4531	0.4590	0.3018

300	NCLDDRCIL	0.0503	0.2134	0.2273	1.0770	0.3013
384	SGNLLLDKR	0.0531	0.2256	0.0576	1.3310	0.3008
754	SMMILSDDA	0.0724	0.3072	0.0869	-0.4010	0.3002
14	VSAARLTPC	0.0685	0.2907	0.0284	0.1040	0.3002
330	VRKIFVDGV	0.0533	0.2265	0.2812	0.6290	0.3001
315	VLFSTVFPP	0.0612	0.2598	0.1131	0.4390	0.2987
465	DIRQLLFVV	0.0563	0.2392	0.3378	0.1690	0.2983
727	LYECLYRNR	0.0446	0.1894	0.1450	1.7410	0.2982
661	QVLSEMVMC	0.0652	0.2768	0.0274	0.3380	0.2978
800	WTETDLTKG	0.0877	0.3725	0.0298	-1.5950	0.2972
403	NNVAFQTVK	0.0562	0.2386	0.2407	0.4370	0.2966
792	VFMSEAKCW	0.0450	0.1909	0.2629	1.3200	0.2963
631	RIMASLVLA	0.0669	0.2841	0.0914	-0.0500	0.2953
273	YDFTEERLK	0.0610	0.2592	0.1179	0.3500	0.2944
839	GAGCFVDDI	0.0626	0.2658	0.0919	0.2930	0.2942
158	NKKDWYDFV	0.0556	0.2360	0.2887	0.2940	0.2940
58	EKDEDDNLI	0.0628	0.2665	0.1248	0.1550	0.2930
826	YVYLPYPDP	0.0618	0.2626	0.1072	0.2710	0.2922
679	GTSSGDATT	0.0774	0.3288	0.0676	-0.9400	0.2920
102	FKFRIDGDM	0.0616	0.2617	0.1097	0.2660	0.2914
327	GPLVRKIFV	0.0497	0.2112	0.6057	-0.2560	0.2892
884	YLQYIRKLH	0.0718	0.3048	0.0668	-0.5200	0.2888
789	QNNVFMSEA	0.0658	0.2795	0.3328	-0.8210	0.2884
116	RQRLTKYTM	0.0509	0.2160	0.2391	0.7270	0.2883
280	LKLFDRYFK	0.0494	0.2099	0.2961	0.6680	0.2877
117	QRLTKYTMA	0.0598	0.2541	0.2778	-0.1660	0.2875
223	IQTTPGSGV	0.0578	0.2456	0.1863	0.2720	0.2871
298	CVNCLDDRC	0.0649	0.2754	0.0222	0.1590	0.2867
140	DTLKEILVT	0.0716	0.3041	0.2250	-1.0320	0.2863
362	HSSRLSFKE	0.0843	0.3581	0.0437	-1.5720	0.2860
400	ALTNNVAFQ	0.0624	0.2650	0.0767	0.1760	0.2854
795	SEAKCWTET	0.0693	0.2943	0.1830	-0.7350	0.2850
840	AGCFVDDIV	0.0568	0.2413	0.2324	0.1710	0.2847
486	GCINANQVI	0.0541	0.2297	0.2154	0.4150	0.2828
404	NVAFQTVKP	0.0600	0.2546	0.0404	0.4380	0.2826
515	YYDSMSYED	0.0820	0.3481	0.1273	-1.6990	0.2823
651	RFYRLANEC	0.0580	0.2464	0.0418	0.5900	0.2822
224	QTTPGSGVP	0.0640	0.2717	0.0463	0.0690	0.2821
361	LHSSRLSFK	0.0537	0.2280	0.1488	0.6130	0.2810
814	SQHTMLVKQ	0.0635	0.2695	0.0575	0.0460	0.2804
135	DEGNCDTLK	0.0609	0.2584	0.2287	-0.2550	0.2799

518	SMSYEDQDA	0.0704	0.2988	0.0407	-0.5040	0.2797
621	KCDRAMPNM	0.0621	0.2635	0.0709	0.1110	0.2796
622	CDRAMPNML	0.0522	0.2218	0.1292	0.7560	0.2790
867	YPLTKHPNQ	0.0564	0.2395	0.3477	-0.2580	0.2788
548	ISAKNRART	0.0700	0.2973	0.0257	-0.4710	0.2776
219	FGDFIQTTP	0.0696	0.2956	0.0645	-0.5720	0.2767
578	SIAATRGAT	0.0691	0.2933	0.0232	-0.4060	0.2765
831	YPDPSRILG	0.0712	0.3022	0.4953	-2.0410	0.2745
903	YSVMLTNDN	0.0775	0.3291	0.0267	-1.1810	0.2740
387	LLLDKRTTC	0.0597	0.2533	0.0727	0.1970	0.2740
235	DSYYSLLMP	0.0629	0.2669	0.0259	0.0630	0.2739
562	ICSTMTNRQ	0.0630	0.2675	0.0368	-0.0120	0.2724
542	MNLKYAISA	0.0642	0.2724	0.2187	-0.6720	0.2716
604	TVYSDVENP	0.0566	0.2401	0.0375	0.5100	0.2713
185	ALLKTVQFC	0.0607	0.2578	0.0382	0.1470	0.2709
89	LLKDCPAVA	0.0507	0.2155	0.4881	-0.3730	0.2700
464	CDIRQLLFV	0.0603	0.2561	0.0685	0.0710	0.2699
139	CDTLKEILV	0.0551	0.2340	0.2145	0.0710	0.2698
244	ILTLTRALT	0.0596	0.2529	0.3913	-0.8440	0.2694
616	GWDYPKCDR	0.0437	0.1856	0.0760	1.4120	0.2676
163	YDFVENPDI	0.0566	0.2405	0.0929	0.2630	0.2676
370	ELLVYAADP	0.0604	0.2564	0.0603	0.0390	0.2674
205	LTLDNQDLN	0.0766	0.3251	0.0322	-1.2680	0.2666
331	RKIFVDGVP	0.0558	0.2368	0.0262	0.5140	0.2664
860	VSLAIDAYP	0.0599	0.2542	0.0369	0.1200	0.2657
919	EFYEAMYTP	0.0543	0.2306	0.1966	0.1100	0.2656
75	HTFSNYQHE	0.0778	0.3302	0.0347	-1.4340	0.2638
208	DNQDLNGNW	0.0504	0.2140	0.1844	0.4320	0.2633
701	TANVNALLS	0.0878	0.3727	0.0241	-2.2610	0.2632
682	SGDATTAYA	0.0650	0.2760	0.2028	-0.8710	0.2629
129	YALRHFDEG	0.0739	0.3139	0.0907	-1.2980	0.2626
302	LDDRCILHC	0.0662	0.2809	0.0301	-0.4720	0.2619
367	SFKELLVYA	0.0502	0.2133	0.4126	-0.2690	0.2618
663	LSEMVMCGG	0.0776	0.3293	0.0271	-1.4480	0.2610
614	LMGWDYPKC	0.0604	0.2564	0.0435	-0.0460	0.2606
904	SVMLTNDNT	0.0662	0.2810	0.0304	-0.5010	0.2605
862	LAIDAYPLT	0.0646	0.2742	0.0800	-0.5260	0.2599
901	DMYSVMLTN	0.0719	0.3052	0.1167	-1.2750	0.2589
790	NNVFMSEAK	0.0509	0.2162	0.1549	0.3780	0.2583
39	NDKVAGFAK	0.0482	0.2046	0.3109	0.1400	0.2582
841	GCFVDDIVK	0.0499	0.2117	0.1188	0.5720	0.2581

57	QEKDEDDNL	0.0489	0.2074	0.0908	0.7250	0.2573
920	FYEAMYTPH	0.0649	0.2758	0.1015	-0.6770	0.2571
808	GPHEFCSQH	0.0484	0.2056	0.6388	-0.8880	0.2570
43	AGFAKFLKT	0.0578	0.2456	0.2915	-0.6690	0.2559
691	NSVFNICQA	0.0589	0.2502	0.1111	-0.2260	0.2556
638	LARKHTTCC	0.0534	0.2268	0.1342	0.1670	0.2553
769	TYASQGLVA	0.0560	0.2378	0.1861	-0.2210	0.2547
791	NVFMSEAKC	0.0538	0.2284	0.0273	0.4430	0.2546
874	NQEYADVFH	0.0674	0.2861	0.0231	-0.7120	0.2540
453	YDYYRYNLP	0.0607	0.2578	0.0314	-0.1750	0.2537
921	YEAMYTPHT	0.0672	0.2855	0.1015	-0.9490	0.2533
757	ILSDDAVVC	0.0568	0.2412	0.0508	0.0890	0.2533
559	GVSICSTMT	0.0642	0.2724	0.0656	-0.5960	0.2525
635	SLVLARKHT	0.0553	0.2348	0.2502	-0.4010	0.2522
320	VFPPTSFGP	0.0533	0.2264	0.0609	0.3290	0.2520
574	KLLKSIAAT	0.0632	0.2682	0.0951	-0.6100	0.2519
527	LFAYTKRNV	0.0499	0.2118	0.0471	0.6570	0.2517
798	KCWTETDLT	0.0588	0.2496	0.0968	-0.2520	0.2516
605	VYSDVENPH	0.0476	0.2020	0.3984	-0.2030	0.2516
311	ANFNVLFST	0.0575	0.2440	0.2529	-0.6090	0.2515
653	YRLANECAQ	0.0581	0.2465	0.0348	-0.0060	0.2514
18	RLTPCGTGT	0.0609	0.2585	0.0589	-0.3240	0.2512
183	RQALLKTVQ	0.0546	0.2318	0.0339	0.2780	0.2508
419	FYDFAVSKG	0.0612	0.2600	0.4499	-1.5410	0.2505
248	TRALTAESH	0.0575	0.2441	0.1359	-0.2850	0.2502
722	NLQHRLYEC	0.0557	0.2363	0.0317	0.1710	0.2496
314	NVLFSTVFP	0.0554	0.2354	0.0300	0.1930	0.2495
672	SLYVKPGGT	0.0591	0.2508	0.1497	-0.4760	0.2494
636	LVLARKHTT	0.0576	0.2444	0.1926	-0.4910	0.2487
85	TIYNLLKDC	0.0560	0.2376	0.0252	0.1420	0.2484
858	RFVSLAIDA	0.0576	0.2447	0.0921	-0.2130	0.2479
259	TDLTKPYIK	0.0535	0.2272	0.1321	0.0150	0.2477
349	RELGVVHNQ	0.0526	0.2232	0.1889	-0.0840	0.2473
505	PFNKWGKAR	0.0449	0.1908	0.0516	0.9730	0.2472
254	ESHVDTDLT	0.0651	0.2764	0.0268	-0.6850	0.2462
523	DQDALFAYT	0.0706	0.2999	0.0671	-1.2890	0.2455
143	KEILVTYNC	0.0541	0.2296	0.0632	0.1030	0.2442
474	EVVDKYFDC	0.0556	0.2361	0.0291	0.0740	0.2441
80	YQHEETIYN	0.0678	0.2878	0.0687	-1.0890	0.2436
634	ASLVLARKH	0.0591	0.2507	0.0475	-0.2970	0.2430
198	NAGIVGVLT	0.0631	0.2680	0.0485	-0.6470	0.2430

910	DNTSRYWEP	0.0592	0.2512	0.0292	-0.2590	0.2427
19	LTPCGTGTS	0.0815	0.3460	0.0265	-2.1460	0.2427
193	CDAMRNAGI	0.0539	0.2290	0.0591	0.0980	0.2427
133	HFDEGNCDT	0.0639	0.2711	0.0327	-0.6730	0.2424
103	KFRIDGDMV	0.0482	0.2046	0.0703	0.5330	0.2418
181	RVRQALLKT	0.0604	0.2563	0.0470	-0.4410	0.2413
368	FKELLVYAA	0.0615	0.2611	0.0908	-0.6710	0.2412
177	NLGERVRQA	0.0563	0.2388	0.1734	-0.4880	0.2404
337	GVPFVVSTG	0.0542	0.2300	0.5983	-1.5900	0.2402
218	DFGDFIQTT	0.0546	0.2319	0.4113	-1.0690	0.2401
161	DWYDFVENP	0.0515	0.2186	0.0343	0.3230	0.2399
125	ADLVYALRH	0.0600	0.2549	0.1139	-0.6450	0.2398
708	LSTDGNKIA	0.0591	0.2507	0.0862	-0.4790	0.2397
669	CGGSLYVKP	0.0588	0.2499	0.0363	-0.3300	0.2388
583	RGATVVIGT	0.0634	0.2693	0.0289	-0.7010	0.2385
480	FDCYDGGCI	0.0533	0.2263	0.0497	0.0890	0.2382
729	ECLYRNRDV	0.0524	0.2224	0.0714	0.0970	0.2380
440	FFFAQDGNA	0.0554	0.2350	0.1413	-0.3740	0.2375
373	VYAADPAMH	0.0494	0.2097	0.2330	-0.1440	0.2375
529	AYTKRNVIP	0.0502	0.2131	0.0370	0.3720	0.2372
371	LLVYAADPA	0.0611	0.2596	0.0511	-0.6010	0.2372
690	ANSVFNICQ	0.0566	0.2402	0.0357	-0.1700	0.2370
299	VNCLDDRCI	0.0482	0.2048	0.0649	0.4460	0.2368
342	VSTGYHFRE	0.0717	0.3044	0.0303	-1.4520	0.2364
577	KSIAATRGA	0.0576	0.2446	0.0303	-0.2710	0.2356
284	DRYFKYWDQ	0.0503	0.2136	0.1854	-0.1330	0.2347
435	VELKHFFFA	0.0574	0.2436	0.1166	-0.5370	0.2342
137	GNCDTLKEI	0.0510	0.2163	0.1036	0.0450	0.2341
626	MPNMLRIMA	0.0563	0.2389	0.2116	-0.7330	0.2340
842	CFVDDIVKT	0.0519	0.2205	0.2658	-0.5310	0.2339
63	DNLIDSYFV	0.0495	0.2103	0.2469	-0.2750	0.2336
454	DYYRYNLPT	0.0529	0.2248	0.2625	-0.6220	0.2331
289	YWDQTYHPN	0.0671	0.2850	0.0416	-1.1640	0.2331
87	YNLLKDCPA	0.0595	0.2528	0.0909	-0.6810	0.2324
694	FNICQAVTA	0.0605	0.2568	0.0579	-0.6670	0.2322
46	AKFLKTNCC	0.0491	0.2087	0.0835	0.2150	0.2319
394	TCFSVAALT	0.0604	0.2564	0.0303	-0.5840	0.2317
67	DSYFVVKRH	0.0536	0.2275	0.2318	-0.6150	0.2315
535	VIPTITQMN	0.0649	0.2756	0.0389	-0.9990	0.2315
533	RNVIPTITQ	0.0488	0.2072	0.1069	0.1650	0.2315
217	YDFGDFIQT	0.0576	0.2447	0.2371	-0.9770	0.2314

17	ARLTPCGTG	0.0569	0.2414	0.2227	-0.8730	0.2312
485	GGCINANQV	0.0527	0.2240	0.1305	-0.2610	0.2305
805	LTKGPHEFC	0.0550	0.2337	0.0260	-0.1440	0.2304
788	YQNNVFMSE	0.0695	0.2951	0.1154	-1.6560	0.2296
608	DVENPHLMG	0.0695	0.2953	0.1162	-1.6630	0.2295
717	DKYVRNLQH	0.0528	0.2241	0.1879	-0.4610	0.2293
702	ANVNALLST	0.0585	0.2484	0.0790	-0.6260	0.2289
532	KRNVIPTIT	0.0513	0.2180	0.2318	-0.4760	0.2289
550	AKNRARTVA	0.0539	0.2290	0.1260	-0.3820	0.2288
4	AQSFLNRVC	0.0518	0.2199	0.0235	0.0910	0.2280
168	NPDILRVYA	0.0495	0.2103	0.4971	-1.1460	0.2276
843	FVDDIVKTD	0.0742	0.3152	0.0648	-1.9550	0.2272
494	IVNNLDKSA	0.0538	0.2284	0.1285	-0.4210	0.2266
131	LRHFDEGNC	0.0475	0.2017	0.0298	0.4090	0.2266
726	RLYECLYRN	0.0613	0.2605	0.0685	-0.8880	0.2264
516	YDSMSYEDQ	0.0583	0.2474	0.0232	-0.4890	0.2264
144	EILVTYNCC	0.0517	0.2193	0.0247	0.0580	0.2259
560	VSICSTMTN	0.0656	0.2785	0.0391	-1.1740	0.2257
317	FSTVFPPTS	0.0778	0.3302	0.0644	-2.2880	0.2255
618	DYPKCDRAM	0.0465	0.1976	0.1244	0.1820	0.2253
482	CYDGGCINA	0.0552	0.2345	0.1466	-0.6270	0.2251
557	VAGVSICST	0.0601	0.2551	0.0408	-0.7440	0.2240
45	FAKFLKTNC	0.0521	0.2210	0.0385	-0.0600	0.2238
905	VMLTNDNTS	0.0720	0.3056	0.1164	-1.9900	0.2236
714	KIADKYVRN	0.0622	0.2641	0.0377	-0.9220	0.2236
423	AVSKGFFKE	0.0623	0.2645	0.1912	-1.3920	0.2236
865	DAYPLTKHP	0.0504	0.2142	0.0494	0.0370	0.2234
316	LFSTVFPPT	0.0550	0.2334	0.0968	-0.4910	0.2234
287	FKYWDQTYH	0.0555	0.2356	0.0595	-0.4460	0.2222
285	RYFKYWDQT	0.0522	0.2215	0.0717	-0.2240	0.2211
382	AASGNLLLD	0.0692	0.2937	0.0629	-1.6460	0.2209
539	ITQMNLKYA	0.0571	0.2423	0.0388	-0.5560	0.2204
187	LKTVQFCDA	0.0526	0.2235	0.1349	-0.4700	0.2202
192	FCDAMRNAG	0.0708	0.3006	0.0226	-1.6950	0.2193
396	FSVAALTNN	0.0658	0.2794	0.0256	-1.2990	0.2183
431	EGSSVELKH	0.0561	0.2383	0.1673	-0.9090	0.2179
781	NFKSVLYYQ	0.0472	0.2004	0.0490	0.1980	0.2176
310	CANFNVLFS	0.0780	0.3311	0.0249	-2.3510	0.2172
290	WDQTYHPNC	0.0548	0.2326	0.0304	-0.4040	0.2170
765	CFNSTYASQ	0.0501	0.2126	0.0346	-0.0260	0.2165
573	QKLLKSIAA	0.0541	0.2298	0.0690	-0.4780	0.2162

770	YASQGLVAS	0.0757	0.3216	0.0295	-2.2070	0.2156
441	FFAQDGNAA	0.0558	0.2368	0.0427	-0.5510	0.2156
176	ANLGERVRQ	0.0515	0.2187	0.0280	-0.1480	0.2155
191	QFCDAMRNA	0.0530	0.2250	0.0320	-0.2990	0.2149
436	ELKHFFFAQ	0.0497	0.2111	0.1003	-0.2450	0.2139
293	TYHPNCVNC	0.0455	0.1930	0.0591	0.2390	0.2139
395	CFSVAALTN	0.0632	0.2684	0.0286	-1.1830	0.2136
824	DDYVYLPYP	0.0508	0.2155	0.1071	-0.3620	0.2135
74	RHTFSNYQH	0.0460	0.1952	0.1913	-0.2190	0.2130
347	HFRELGVVH	0.0507	0.2153	0.1372	-0.4620	0.2128
222	FIQTTPGSG	0.0672	0.2855	0.0257	-1.5330	0.2127
154	DDYFNKKDW	0.0387	0.1644	0.2182	0.3090	0.2126
619	YPKCDRAMP	0.0527	0.2239	0.0404	-0.3610	0.2119
712	GNKIADKYV	0.0441	0.1871	0.2174	-0.1580	0.2118
761	DAVVCFNST	0.0555	0.2357	0.1797	-1.0300	0.2111
115	SRQRLTKYT	0.0527	0.2237	0.0709	-0.4690	0.2109
252	TAESHVDTD	0.0709	0.3010	0.0235	-1.8800	0.2105
292	QTYHPNCVN	0.0568	0.2414	0.2188	-1.2760	0.2104
744	EFYAYLRKH	0.0503	0.2134	0.0532	-0.2440	0.2092
652	FYRLANECA	0.0476	0.2020	0.1061	-0.1850	0.2087
603	KTVYSDVEN	0.0611	0.2594	0.0240	-1.1040	0.2078
572	HQKLLKSIA	0.0541	0.2298	0.0717	-0.6580	0.2077
460	LPTMCDIRQ	0.0499	0.2120	0.0711	-0.3010	0.2076
544	LKYAISAKN	0.0548	0.2328	0.1427	-0.9340	0.2075
354	VHNQDVNLH	0.0505	0.2143	0.1251	-0.5130	0.2074
766	FNSTYASQG	0.0671	0.2848	0.0348	-1.6540	0.2073
201	IVGVLTLDN	0.0637	0.2705	0.0249	-1.3470	0.2068
695	NICQAVTAN	0.0623	0.2645	0.0260	-1.2460	0.2061
86	IYNLLKDCP	0.0439	0.1864	0.0243	0.3080	0.2055
68	SYFVVKRHT	0.0455	0.1933	0.2189	-0.4150	0.2054
870	TKHPNQEYA	0.0506	0.2148	0.0779	-0.4290	0.2050
415	FNKDFYDFA	0.0544	0.2308	0.0383	-0.6440	0.2044
171	ILRVYANLG	0.0600	0.2548	0.1436	-1.4430	0.2041
526	ALFAYTKRN	0.0595	0.2525	0.0663	-1.1790	0.2035
408	QTVKPGNFN	0.0628	0.2664	0.0253	-1.3510	0.2027
662	VLSEMVMCG	0.0616	0.2617	0.0589	-1.3670	0.2022
16	AARLTPCGT	0.0513	0.2179	0.0292	-0.4060	0.2019
787	YYQNNVFMS	0.0665	0.2823	0.1236	-1.9850	0.2016
801	TETDLTKGP	0.0475	0.2015	0.0421	-0.1440	0.2006
91	KDCPAVAKH	0.0513	0.2180	0.1068	-0.6790	0.2001
20	TPCGTGTST	0.0540	0.2295	0.1231	-0.9740	0.1992

12	CGVSAARLT	0.0563	0.2389	0.0241	-0.8880	0.1981
878	ADVFHLYLQ	0.0471	0.1999	0.0643	-0.2430	0.1974
195	AMRNAGIVG	0.0580	0.2464	0.0767	-1.2200	0.1969
118	RLTKYTMAD	0.0640	0.2718	0.0436	-1.6370	0.1965
49	LKTNCCRFQ	0.0473	0.2007	0.0335	-0.1930	0.1961
697	CQAVTANVN	0.0596	0.2533	0.0331	-1.2550	0.1955
348	FRELGVVHN	0.0568	0.2412	0.1484	-1.3710	0.1950
247	LTRALTAES	0.0690	0.2931	0.0788	-2.2040	0.1947
355	HNQDVNLHS	0.0714	0.3033	0.1000	-2.4740	0.1946
687	TAYANSVFN	0.0576	0.2445	0.0270	-1.0870	0.1942
386	NLLLDKRTT	0.0517	0.2195	0.0503	-0.6610	0.1940
783	KSVLYYQNN	0.0586	0.2488	0.0284	-1.1950	0.1933
674	YVKPGGTSS	0.0624	0.2651	0.2150	-2.0810	0.1933
900	LDMYSVMLT	0.0553	0.2348	0.0232	-0.9080	0.1929
306	CILHCANFN	0.0573	0.2434	0.0227	-1.0780	0.1929
202	VGVLTLDNQ	0.0471	0.2000	0.0268	-0.2230	0.1928
888	IRKLHDELT	0.0496	0.2106	0.0557	-0.5300	0.1924
731	LYRNRDVDT	0.0475	0.2016	0.0696	-0.3950	0.1923
15	SAARLTPCG	0.0580	0.2464	0.0475	-1.2260	0.1922
675	VKPGGTSSG	0.0531	0.2253	0.2281	-1.3510	0.1920
612	PHLMGWDYP	0.0463	0.1967	0.0288	-0.2090	0.1906
753	FSMMILSDD	0.0653	0.2771	0.0230	-1.8280	0.1891
70	FVVKRHTFS	0.0664	0.2820	0.1012	-2.1760	0.1884
759	SDDAVVCFN	0.0631	0.2681	0.0313	-1.6980	0.1879
588	VIGTSKFYG	0.0590	0.2503	0.0228	-1.3210	0.1877
617	WDYPKCDRA	0.0497	0.2112	0.0800	-0.7130	0.1876
489	NANQVIVNN	0.0581	0.2467	0.0413	-1.3110	0.1874
147	VTYNCCDDD	0.0614	0.2607	0.0248	-1.5400	0.1874
782	FKSVLYYQN	0.0576	0.2445	0.0269	-1.2240	0.1873
716	ADKYVRNLQ	0.0461	0.1955	0.0282	-0.2620	0.1867
736	DVDTDFVNE	0.0640	0.2719	0.0264	-1.7870	0.1865
776	VASIKNFKS	0.0674	0.2862	0.0304	-2.0870	0.1864
752	HFSMMILSD	0.0623	0.2646	0.0244	-1.6420	0.1862
424	VSKGFFKEG	0.0532	0.2259	0.1151	-1.1410	0.1861
596	GGWHNMLKT	0.0503	0.2134	0.1552	-1.0310	0.1851
8	LNRVCGVSA	0.0494	0.2099	0.0804	-0.7400	0.1850
356	NQDVNLHSS	0.0685	0.2906	0.0731	-2.3320	0.1850
269	DLLKYDFTE	0.0616	0.2614	0.1221	-1.9000	0.1847
556	TVAGVSICS	0.0652	0.2770	0.0430	-1.9790	0.1845
693	VFNICQAVT	0.0485	0.2060	0.0269	-0.5160	0.1842
705	NALLSTDGN	0.0561	0.2383	0.0238	-1.1610	0.1838

71	VVKRHTFSN	0.0558	0.2368	0.0529	-1.2220	0.1837
50	KTNCCRFQE	0.0604	0.2563	0.0238	-1.5260	0.1836
728	YECLYRNRD	0.0649	0.2757	0.0234	-1.9140	0.1835
739	TDFVNEFYA	0.0489	0.2075	0.1287	-0.8910	0.1822
159	KKDWYDFVE	0.0589	0.2501	0.0274	-1.4440	0.1820
5	QSFLNRVCG	0.0532	0.2260	0.0608	-1.0760	0.1814
816	HTMLVKQGD	0.0641	0.2723	0.0300	-1.9120	0.1812
832	PDPSRILGA	0.0509	0.2163	0.2235	-1.3900	0.1803
44	GFAKFLKTN	0.0530	0.2250	0.0877	-1.1560	0.1803
329	LVRKIFVDG	0.0572	0.2430	0.0317	-1.3490	0.1803
576	LKSIAATRG	0.0558	0.2370	0.0537	-1.2980	0.1802
807	KGPHEFCSQ	0.0441	0.1872	0.0428	-0.2700	0.1801
592	SKFYGGWHN	0.0541	0.2298	0.0312	-1.0910	0.1799
156	YFNKKDWYD	0.0636	0.2699	0.0320	-1.9010	0.1796
600	NMLKTVYSD	0.0594	0.2524	0.0660	-1.6620	0.1792
76	TFSNYQHEE	0.0580	0.2464	0.0300	-1.4460	0.1786
582	TRGATVVIG	0.0552	0.2345	0.0320	-1.2190	0.1784
584	GATVVIGTS	0.0605	0.2567	0.2340	-2.2760	0.1780
483	YDGGCINAN	0.0624	0.2647	0.0250	-1.8130	0.1778
817	TMLVKQGDD	0.0609	0.2586	0.0259	-1.7200	0.1765
541	QMNLKYAIS	0.0663	0.2815	0.0301	-2.1950	0.1763
56	FQEKDEDDN	0.0565	0.2401	0.0227	-1.3470	0.1761
732	YRNRDVDTD	0.0600	0.2549	0.0280	-1.6750	0.1754
703	NVNALLSTD	0.0590	0.2506	0.0243	-1.5800	0.1753
319	TVFPPTSFG	0.0517	0.2193	0.0762	-1.1120	0.1751
190	VQFCDAMRN	0.0527	0.2237	0.0250	-1.0560	0.1747
35	FDIYNDKVA	0.0482	0.2048	0.0341	-0.7150	0.1741
864	IDAYPLTKH	0.0464	0.1971	0.0627	-0.6490	0.1740
704	VNALLSTDG	0.0556	0.2362	0.0280	-1.3280	0.1740
246	TLTRALTAE	0.0578	0.2455	0.0414	-1.5550	0.1740
834	PSRILGAGC	0.0462	0.1960	0.0229	-0.5150	0.1737
31	VYRAFDIYN	0.0505	0.2142	0.0269	-0.8930	0.1736
268	WDLLKYDFT	0.0509	0.2160	0.0244	-0.9210	0.1736
7	FLNRVCGVS	0.0649	0.2757	0.1045	-2.3610	0.1733
405	VAFQTVKPG	0.0501	0.2125	0.0776	-1.0280	0.1728
339	PFVVSTGYH	0.0472	0.2005	0.0564	-0.7230	0.1728
914	RYWEPEFYE	0.0441	0.1870	0.1799	-0.8310	0.1725
746	YAYLRKHFS	0.0648	0.2750	0.0245	-2.1310	0.1721
32	YRAFDIYND	0.0567	0.2409	0.0598	-1.5650	0.1716
200	GIVGVLTLD	0.0548	0.2326	0.1365	-1.6340	0.1714
491	NQVIVNNLD	0.0556	0.2362	0.0571	-1.4710	0.1712

130	ALRHFDEGN	0.0529	0.2247	0.0278	-1.1550	0.1711
336	DGVPFVVST	0.0506	0.2150	0.0930	-1.1630	0.1708
406	AFQTVKPGN	0.0509	0.2163	0.0234	-0.9890	0.1704
439	HFFFAQDGN	0.0512	0.2173	0.0257	-1.0260	0.1698
295	HPNCVNCLD	0.0603	0.2562	0.2098	-2.3570	0.1698
186	LLKTVQFCD	0.0568	0.2413	0.0640	-1.6400	0.1689
889	RKLHDELTG	0.0513	0.2177	0.0276	-1.0750	0.1681
488	INANQVIVN	0.0547	0.2323	0.0509	-1.4350	0.1681
142	LKEILVTYN	0.0527	0.2238	0.0928	-1.3960	0.1680
100	DFFKFRIDG	0.0549	0.2332	0.0495	-1.4550	0.1679
833	DPSRILGAG	0.0530	0.2249	0.3367	-2.1650	0.1671
553	RARTVAGVS	0.0590	0.2504	0.0298	-1.7590	0.1669
443	AQDGNAAIS	0.0654	0.2779	0.0276	-2.3070	0.1666
677	PGGTSSGDA	0.0533	0.2264	0.0459	-1.3440	0.1661
145	ILVTYNCCD	0.0599	0.2545	0.0283	-1.8620	0.1657
484	DGGCINANQ	0.0488	0.2070	0.0250	-0.9030	0.1656
438	KHFFFAQDG	0.0495	0.2101	0.0757	-1.1210	0.1654
773	QGLVASIKN	0.0553	0.2347	0.0276	-1.4970	0.1640
837	ILGAGCFVD	0.0557	0.2366	0.1137	-1.8040	0.1635
512	ARLYYDSMS	0.0543	0.2304	0.1436	-1.7760	0.1632
451	SDYDYYRYN	0.0521	0.2212	0.0615	-1.3740	0.1617
649	SHRFYRLAN	0.0498	0.2112	0.0529	-1.1580	0.1613
344	TGYHFRELG	0.0541	0.2297	0.0249	-1.4420	0.1613
52	NCCRFQEKD	0.0575	0.2442	0.0250	-1.7390	0.1610
657	NECAQVLSE	0.0547	0.2323	0.0248	-1.5010	0.1609
514	LYYDSMSYE	0.0478	0.2028	0.0386	-0.9780	0.1597
172	LRVYANLGE	0.0517	0.2193	0.0249	-1.2680	0.1596
250	ALTAESHVD	0.0543	0.2304	0.0438	-1.5560	0.1592
385	GNLLLDKRT	0.0479	0.2032	0.0269	-0.9650	0.1590
845	DDIVKTDGT	0.0506	0.2149	0.0240	-1.2130	0.1579
735	RDVDTDFVN	0.0498	0.2116	0.0271	-1.1600	0.1577
469	LLFVVEVVD	0.0540	0.2291	0.0995	-1.7290	0.1576
150	NCCDDDYFN	0.0514	0.2181	0.0247	-1.3010	0.1568
127	LVYALRHFD	0.0549	0.2332	0.0302	-1.6250	0.1565
751	KHFSMMILS	0.0581	0.2466	0.0260	-1.8870	0.1562
112	PHISRQRLT	0.0483	0.2051	0.0260	-1.0620	0.1559
551	KNRARTVAG	0.0500	0.2124	0.0247	-1.2380	0.1542
493	VIVNNLDKS	0.0588	0.2497	0.0286	-2.0260	0.1527
902	MYSVMLTND	0.0530	0.2249	0.0634	-1.6470	0.1521
203	GVLTLDNQD	0.0560	0.2377	0.0256	-1.7910	0.1520
350	ELGVVHNQD	0.0572	0.2429	0.0708	-2.0310	0.1519

128	VYALRHFDE	0.0485	0.2059	0.0287	-1.1740	0.1515
796	EAKCWTETD	0.0524	0.2224	0.1602	-1.9150	0.1506
721	RNLQHRLYE	0.0511	0.2169	0.0257	-1.4090	0.1503
84	ETIYNLLKD	0.0596	0.2530	0.0241	-2.1390	0.1497
502	AGFPFNKWG	0.0495	0.2102	0.0464	-1.3520	0.1496
36	DIYNDKVAG	0.0518	0.2199	0.0317	-1.5010	0.1496
160	KDWYDFVEN	0.0486	0.2066	0.0284	-1.2430	0.1487
570	QFHQKLLKS	0.0557	0.2366	0.0477	-1.9030	0.1486
885	LQYIRKLHD	0.0521	0.2210	0.0492	-1.6010	0.1483
678	GGTSSGDAT	0.0497	0.2112	0.0232	-1.3320	0.1481
650	HRFYRLANE	0.0487	0.2069	0.0331	-1.2840	0.1476
55	RFQEKDEDD	0.0509	0.2160	0.0243	-1.4430	0.1475
54	CRFQEKDED	0.0517	0.2193	0.0340	-1.5450	0.1472
210	QDLNGNWYD	0.0557	0.2365	0.0539	-1.9510	0.1470
853	TLMIERFVS	0.0598	0.2539	0.0241	-2.2120	0.1469
730	CLYRNRDVD	0.0549	0.2333	0.0239	-1.8170	0.1460
478	KYFDCYDGG	0.0451	0.1914	0.0463	-1.0510	0.1457
683	GDATTAYAN	0.0538	0.2286	0.0238	-1.7310	0.1456
352	GVVHNQDVN	0.0476	0.2020	0.0316	-1.2300	0.1453
886	QYIRKLHDE	0.0469	0.1992	0.0309	-1.1710	0.1452
517	DSMSYEDQD	0.0578	0.2455	0.0231	-2.0780	0.1451
437	LKHFFFAQD	0.0513	0.2179	0.1415	-1.8990	0.1442
410	VKPGNFNKD	0.0526	0.2231	0.0394	-1.7090	0.1436
212	LNGNWYDFG	0.0546	0.2320	0.0253	-1.8460	0.1435
378	PAMHAASGN	0.0543	0.2307	0.0235	-1.8170	0.1434
673	LYVKPGGTS	0.0499	0.2117	0.1581	-1.8450	0.1431
866	AYPLTKHPN	0.0443	0.1879	0.0544	-1.0610	0.1430
303	DDRCILHCA	0.0439	0.1865	0.0605	-1.0620	0.1425
481	DCYDGGCIN	0.0504	0.2141	0.0315	-1.5290	0.1423
304	DRCILHCAN	0.0507	0.2151	0.0328	-1.5550	0.1422
377	DPAMHAASG	0.0518	0.2200	0.0945	-1.8450	0.1419
656	ANECAQVLS	0.0595	0.2527	0.0329	-2.3500	0.1401
602	LKTVYSDVE	0.0499	0.2118	0.0247	-1.5120	0.1399
857	ERFVSLAID	0.0488	0.2074	0.1129	-1.6920	0.1397
444	QDGNAAISD	0.0570	0.2420	0.0245	-2.1190	0.1397
499	DKSAGFPFN	0.0501	0.2128	0.0247	-1.5380	0.1396
838	LGAGCFVDD	0.0560	0.2379	0.0265	-2.0520	0.1393
146	LVTYNCCDD	0.0519	0.2203	0.0230	-1.7000	0.1388
664	SEMVMCGGS	0.0578	0.2454	0.0248	-2.2150	0.1384
589	IGTSKFYGG	0.0505	0.2145	0.0319	-1.6280	0.1379
220	GDFIQTTPG	0.0458	0.1946	0.2444	-1.8750	0.1375

466	IRQLLFVVE	0.0486	0.2065	0.0350	-1.5070	0.1364
270	LLKYDFTEE	0.0486	0.2065	0.0809	-1.6830	0.1344
764	VCFNSTYAS	0.0538	0.2285	0.0346	-2.0040	0.1335
477	DKYFDCYDG	0.0466	0.1977	0.0361	-1.4280	0.1318
132	RHFDEGNCD	0.0459	0.1950	0.0426	-1.4040	0.1312
918	PEFYEAMYT	0.0461	0.1957	0.0263	-1.3760	0.1309
815	QHTMLVKQG	0.0460	0.1951	0.0228	-1.3570	0.1307
213	NGNWYDFGD	0.0544	0.2310	0.0288	-2.0910	0.1307
641	KHTTCCSLS	0.0535	0.2273	0.0276	-2.0350	0.1297
676	KPGGTSSGD	0.0512	0.2173	0.1238	-2.1290	0.1294
261	LTKPYIKWD	0.0522	0.2217	0.0424	-1.9740	0.1294
369	KELLVYAAD	0.0521	0.2211	0.0314	-1.9310	0.1292
844	VDDIVKTDG	0.0517	0.2197	0.0310	-1.9220	0.1282
893	DELTGHMLD	0.0553	0.2346	0.0439	-2.2720	0.1276
871	KHPNQEYAD	0.0490	0.2082	0.0317	-1.7060	0.1276
495	VNNLDKSAG	0.0477	0.2025	0.0228	-1.5710	0.1274
809	PHEFCSQHT	0.0430	0.1827	0.0799	-1.3520	0.1271
266	IKWDLLKYD	0.0471	0.2000	0.0550	-1.6230	0.1271
671	GSLYVKPGG	0.0467	0.1982	0.0395	-1.5460	0.1268
670	GGSLYVKPG	0.0486	0.2064	0.0849	-1.8520	0.1266
615	MGWDYPKCD	0.0486	0.2065	0.0690	-1.8150	0.1261
599	HNMLKTVYS	0.0578	0.2454	0.0307	-2.4800	0.1260
457	RYNLPTMCD	0.0446	0.1892	0.0607	-1.4530	0.1257
276	TEERLKLFD	0.0549	0.2333	0.0227	-2.2210	0.1257
684	DATTAYANS	0.0587	0.2491	0.0263	-2.5750	0.1243
162	WYDFVENPD	0.0504	0.2140	0.0274	-1.8930	0.1234
428	FFKEGSSVE	0.0459	0.1949	0.0276	-1.5250	0.1228
28	TDVVYRAFD	0.0517	0.2194	0.0230	-2.0120	0.1223
620	PKCDRAMPN	0.0484	0.2054	0.0255	-1.7890	0.1197
376	ADPAMHAAS	0.0539	0.2290	0.0278	-2.2700	0.1196
510	GKARLYYDS	0.0547	0.2324	0.0305	-2.4140	0.1162
328	PLVRKIFVD	0.0523	0.2222	0.0340	-2.2280	0.1159
92	DCPAVAKHD	0.0499	0.2119	0.0258	-2.0250	0.1145
207	LDNQDLNGN	0.0458	0.1943	0.0259	-1.7030	0.1130
53	CCRFQEKDE	0.0422	0.1790	0.0224	-1.3960	0.1126
473	VEVVDKYFD	0.0462	0.1962	0.0274	-1.7640	0.1121
509	WGKARLYYD	0.0490	0.2078	0.0282	-2.0100	0.1116
803	TDLTKGPHE	0.0489	0.2076	0.0396	-2.0430	0.1114
101	FFKFRIDGD	0.0474	0.2011	0.0250	-1.8840	0.1106
446	GNAAISDYD	0.0518	0.2201	0.0241	-2.2690	0.1102
425	SKGFFKEGS	0.0516	0.2191	0.0242	-2.2590	0.1098

169	PDILRVYAN	0.0470	0.1997	0.0276	-1.8930	0.1092
868	PLTKHPNQE	0.0492	0.2089	0.0383	-2.1140	0.1090
827	VYLPYPDPS	0.0466	0.1979	0.0453	-1.9530	0.1071
413	GNFNKDFYD	0.0490	0.2082	0.0493	-2.1800	0.1066
99	HDFFKFRID	0.0476	0.2022	0.0284	-2.0010	0.1064
231	VPVVDSYYS	0.0478	0.2031	0.1609	-2.4400	0.1052
896	TGHMLDMYS	0.0529	0.2244	0.0230	-2.4550	0.1051
610	ENPHLMGWD	0.0491	0.2084	0.0224	-2.1400	0.1048
909	NDNTSRYWE	0.0464	0.1972	0.0223	-1.9200	0.1046
21	PCGTGTSTD	0.0521	0.2211	0.0267	-2.4280	0.1037
283	FDRYFKYWD	0.0480	0.2038	0.0260	-2.1210	0.1017
476	VDKYFDCYD	0.0465	0.1974	0.0282	-2.0160	0.1009
639	ARKHTTCCS	0.0439	0.1863	0.0366	-1.8490	0.0993
806	TKGPHEFCS	0.0495	0.2100	0.0267	-2.3320	0.0974
59	KDEDDNLID	0.0493	0.2094	0.0241	-2.3270	0.0967
228	GSGVPVVDS	0.0521	0.2212	0.0674	-2.6920	0.0967
221	DFIQTTPGS	0.0493	0.2093	0.0255	-2.3550	0.0953
359	VNLHSSRLS	0.0502	0.2130	0.0241	-2.4570	0.0938
426	KGFFKEGSS	0.0473	0.2009	0.0261	-2.2390	0.0928
60	DEDDNLIDS	0.0533	0.2262	0.0346	-2.7720	0.0927
136	EGNCDTLKE	0.0474	0.2012	0.0243	-2.2690	0.0914
417	KDFYDFAVS	0.0442	0.1877	0.0759	-2.1930	0.0895
296	PNCVNCLDD	0.0490	0.2082	0.0241	-2.4620	0.0887
825	DYVYLPYPD	0.0422	0.1792	0.0252	-1.9280	0.0866
627	PNMLRIMAS	0.0512	0.2173	0.0252	-2.7770	0.0822
107	DGDMVPHIS	0.0534	0.2267	0.0288	-2.9950	0.0812
760	DDAVVCFNS	0.0508	0.2157	0.0311	-2.7980	0.0804
389	LDKRTTCFS	0.0473	0.2009	0.0279	-2.5030	0.0799
335	VDGVPFVVS	0.0486	0.2063	0.0483	-2.7590	0.0756
227	PGSGVPVVD	0.0445	0.1889	0.0623	-2.5560	0.0704
153	DDDYFNKKD	0.0461	0.1957	0.0311	-2.6220	0.0693

Table S4. List of predicted CTL from SARS-CoV-2 Nsp13 helicase. → -E represents the MHC ligands and top epitopes.

NetCTL-1.2 predictions using MHC supertype A1. Threshold 0.750000

Number of MHC ligands 19 identified. Number of peptides 593. Protein name Nsp13

**Residue No.**	**Peptide Sequence**	**Predicted MHC binding affinity**	**Rescale binding affinity**	**C-terminal cleavage affinity**	**Transport** efficiency	**Prediction score**	**Identified MHC** ligand
57	VTDVTQLYL	0.4708	1.9988	0.6073	0.6800	2.1239	<-E
190	NSKVQIGEY	0.3664	1.5555	0.9654	3.1010	1.8553	<-E
468	SAQCFKMFY	0.3537	1.5018	0.7388	3.0990	1.7676	<-E
316	ALCEKALKY	0.2955	1.2546	0.8753	2.9580	1.5337	<-E
56	DVTDVTQLY	0.2890	1.2271	0.9651	2.7040	1.5071	<-E
258	ISDEFSSNV	0.3113	1.3216	0.9348	0.3430	1.4790	<-E
535	SSQGSEYDY	0.2761	1.1724	0.8149	2.8470	1.4370	<-E
291	FAIGLALYY	0.2670	1.1338	0.5534	2.9280	1.3633	<-E
306	YTACSHAAV	0.2786	1.1829	0.6004	0.3370	1.2898	<-E
141	TEETFKLSY	0.1837	0.7800	0.9319	2.7780	1.0587	<-E
238	PTLVPQEHY	0.1794	0.7617	0.8719	2.5950	1.0222	<-E
448	IVDTVSALV	0.1991	0.8453	0.8977	0.1330	0.9866	<-E
103	VTDFNAIAT	0.2348	0.9967	0.0576	-0.7000	0.9704	<-E
574	CIMSDRDLY	0.1634	0.6937	0.1836	3.1250	0.8775	<-E
347	KVNSTLEQY	0.1391	0.5907	0.8156	2.9710	0.8616	<-E
269	YQKVGMQKY	0.1138	0.4832	0.9732	3.1190	0.7851	<-E
245	HYVRITGLY	0.1102	0.4678	0.9598	3.0090	0.7622	<-E
85	ANGQVFGLY	0.1141	0.4845	0.9132	2.7460	0.7588	<-E
538	GSEYDYVIF	0.1401	0.5947	0.3528	2.2030	0.7578	<-E
209	VVYRGTTTY	0.1008	0.4279	0.9784	3.4660	0.7480	
298	YYPSARIVY	0.1028	0.4366	0.9743	3.2020	0.7428	
62	QLYLGGMSY	0.1027	0.4361	0.9783	3.1540	0.7405	
227	LTSHTVMPL	0.1259	0.5347	0.9325	0.9800	0.7236	
114	WTNAGDYIL	0.1347	0.5719	0.7199	0.6730	0.7135	
290	HFAIGLALY	0.0973	0.4130	0.9332	3.0720	0.7066	
126	CTERLKLFA	0.1724	0.7318	0.0403	-0.7320	0.7013	
40	KLVLSVNPY	0.0935	0.3969	0.9737	3.1060	0.6983	
390	RLRAKHYVY	0.0877	0.3724	0.9757	3.3910	0.6883	
533	VDSSQGSEY	0.1049	0.4454	0.6924	2.6850	0.6835	
158	LSDRELHLS	0.1856	0.7882	0.0446	-2.3300	0.6784	
213	GTTTYKLNV	0.1480	0.6284	0.2012	0.2100	0.6691	

507	RKAVFISPY	0.0909	0.3858	0.8260	3.1790	0.6687
262	FSSNVANYQ	0.1551	0.6587	0.0664	-0.3310	0.6521
349	NSTLEQYVF	0.0951	0.4038	0.7366	2.6230	0.6454
576	MSDRDLYDK	0.1321	0.5608	0.4288	0.3850	0.6444
124	NTCTERLKL	0.1168	0.4957	0.5878	1.1330	0.6406
63	LYLGGMSYY	0.0779	0.3306	0.9789	3.1620	0.6355
378	MATNYDLSV	0.1131	0.4800	0.7159	0.4320	0.6090
177	NRNYVFTGY	0.0706	0.2998	0.9762	3.1220	0.6023
37	TSHKLVLSV	0.1162	0.4935	0.5779	0.4180	0.6011
449	VDTVSALVY	0.0750	0.3185	0.8868	2.6520	0.5841
216	TYKLNVGDY	0.0658	0.2794	0.9120	3.1760	0.5750
261	EFSSNVANY	0.0679	0.2884	0.9103	2.7450	0.5622
99	GSDNVTDFN	0.1494	0.6342	0.0312	-1.5830	0.5597
365	ETTADIVVF	0.0792	0.3361	0.7452	2.1280	0.5543
254	PTLNISDEF	0.0881	0.3740	0.4393	1.9990	0.5398
73	KSHKPPISF	0.0611	0.2594	0.9643	2.7050	0.5393
520	AVASKILGL	0.0818	0.3473	0.7986	1.3950	0.5368
355	YVFCTVNAL	0.0787	0.3340	0.9482	1.1360	0.5330
388	NARLRAKHY	0.0677	0.2875	0.5560	3.1100	0.5264
480	ITHDVSSAI	0.0819	0.3479	0.9686	0.6130	0.5238
192	KVQIGEYTF	0.0552	0.2342	0.9684	2.8020	0.5196
211	YRGTTTYKL	0.0749	0.3181	0.9706	1.0250	0.5149
464	HKDKSAQCF	0.0821	0.3485	0.2769	2.4710	0.5136
157	VLSDRELHL	0.0772	0.3276	0.9464	0.8650	0.5128
546	FTQTTETAH	0.1254	0.5323	0.0546	-0.6750	0.5068
112	CDWTNAGDY	0.0654	0.2776	0.5958	2.7580	0.5048
413	TKGTLEPEY	0.0692	0.2940	0.4351	2.8380	0.5012
33	HVISTSHKL	0.0729	0.3096	0.9659	0.9320	0.5011
338	ARVECFDKF	0.0535	0.2272	0.7940	3.0950	0.5011
194	QIGEYTFEK	0.0791	0.3359	0.9303	0.4900	0.4999
453	SALVYDNKL	0.0699	0.2967	0.9748	1.1250	0.4992
122	LANTCTERL	0.0808	0.3433	0.7274	0.9260	0.4987
137	TLKATEETF	0.0571	0.2425	0.9007	2.3640	0.4958
383	DLSVVNARL	0.0754	0.3200	0.9703	0.6020	0.4957
263	SSNVANYQK	0.0951	0.4036	0.4823	0.3590	0.4939
467	KSAQCFKMF	0.0808	0.3432	0.1448	2.5600	0.4929
374	DEISMATNY	0.0629	0.2672	0.6927	2.4370	0.4929
172	PRPPLNRNY	0.0512	0.2173	0.9224	2.7370	0.4925
404	QLPAPRTLL	0.0682	0.2896	0.9710	1.1260	0.4916
6	VLCNSQTSL	0.0702	0.2980	0.9330	1.0380	0.4898
35	ISTSHKLVL	0.0823	0.3496	0.5853	1.0290	0.4888

379	ATNYDLSVV	0.0876	0.3717	0.6486	0.3790	0.4880
430	KTIGPDMFL	0.0672	0.2855	0.9581	1.1450	0.4865
409	RTLLTKGTL	0.0698	0.2965	0.8951	1.0500	0.4832
147	LSYGIATVR	0.0595	0.2528	0.9296	1.8170	0.4831
185	YRVTKNSKV	0.0737	0.3128	0.9487	0.5490	0.4825
559	NRFNVAITR	0.0587	0.2492	0.9133	1.8870	0.4805
584	KLQFTSLEI	0.0761	0.3233	0.8690	0.4900	0.4781
146	KLSYGIATV	0.0737	0.3128	0.9673	0.3320	0.4745
75	HKPPISFPL	0.0661	0.2807	0.9726	0.9520	0.4742
203	GDYGDAVVY	0.0459	0.1947	0.9539	2.7260	0.4741
549	TTETAHSCN	0.1279	0.5430	0.0244	-1.5510	0.4691
309	CSHAAVDAL	0.0850	0.3610	0.3376	1.1380	0.4686
248	RITGLYPTL	0.0598	0.2537	0.9767	1.3380	0.4671
217	YKLNVGDYF	0.0713	0.3027	0.2309	2.5920	0.4669
139	KATEETFKL	0.0630	0.2676	0.9525	1.1260	0.4668
429	MKTIGPDMF	0.0532	0.2257	0.7255	2.6430	0.4667
516	NSQNAVASK	0.0751	0.3188	0.8288	0.4580	0.4660
503	NPAWRKAVF	0.0494	0.2096	0.8949	2.4160	0.4646
41	LVLSVNPYV	0.0678	0.2879	0.9628	0.5760	0.4611
332	RIIPARARV	0.0665	0.2822	0.9200	0.8100	0.4607
491	PQIGVVREF	0.0539	0.2287	0.7540	2.3310	0.4584
218	KLNVGDYFV	0.0723	0.3069	0.9116	0.2950	0.4584
232	VMPLSAPTL	0.0647	0.2749	0.8340	1.1330	0.4567
565	ITRAKVGIL	0.0637	0.2704	0.8533	1.1590	0.4563
500	LTRNPAWRK	0.0821	0.3486	0.5875	0.3910	0.4563
312	AAVDALCEK	0.0663	0.2815	0.9222	0.7160	0.4557
435	DMFLGTCRR	0.0589	0.2501	0.9476	1.2570	0.4551
452	VSALVYDNK	0.0879	0.3733	0.3396	0.5940	0.4539
84	CANGQVFGL	0.0682	0.2897	0.7867	0.9200	0.4537
225	FVLTSHTVM	0.0708	0.3007	0.9101	0.3140	0.4529
95	NTCVGSDNV	0.0989	0.4198	0.0731	0.4400	0.4528
362	ALPETTADI	0.0659	0.2800	0.8741	0.8240	0.4523
494	GVVREFLTR	0.0593	0.2517	0.8251	1.5310	0.4520
131	KLFAAETLK	0.0650	0.2759	0.9121	0.7510	0.4502
289	SHFAIGLAL	0.0587	0.2492	0.9163	1.2280	0.4481
153	TVREVLSDR	0.0544	0.2309	0.8461	1.8050	0.4481
489	NRPQIGVVR	0.0523	0.2219	0.9330	1.7220	0.4479
587	FTSLEIPRR	0.0784	0.3328	0.3275	1.3150	0.4477
150	GIATVREVL	0.0642	0.2727	0.8732	0.8690	0.4471
376	ISMATNYDL	0.0783	0.3325	0.4438	0.9160	0.4449
553	AHSCNVNRF	0.0521	0.2212	0.5881	2.6920	0.4440

197	EYTFEKGDY	0.0578	0.2453	0.3498	2.9190	0.4437
121	ILANTCTER	0.0659	0.2797	0.5873	1.4920	0.4424
447	EIVDTVSAL	0.0577	0.2449	0.9295	1.1110	0.4399
296	ALYYPSARI	0.0591	0.2509	0.9457	0.9330	0.4394
476	YKGVITHDV	0.0724	0.3073	0.8521	0.0760	0.4390
567	RAKVGILCI	0.0597	0.2536	0.9279	0.8390	0.4348
130	LKLFAAETL	0.0558	0.2370	0.9304	1.1580	0.4344
285	GTGKSHFAI	0.0781	0.3318	0.6636	0.0260	0.4326
557	NVNRFNVAI	0.0751	0.3187	0.5532	0.6090	0.4321
272	VGMQKYSTL	0.0566	0.2404	0.9707	0.9160	0.4318
224	YFVLTSHTV	0.0631	0.2680	0.9449	0.4010	0.4298
174	PPLNRNYVF	0.0457	0.1939	0.9362	1.9000	0.4293
391	LRAKHYVYI	0.0619	0.2629	0.8034	0.8940	0.4281
82	PLCANGQVF	0.0570	0.2419	0.5808	1.9780	0.4279
344	DKFKVNSTL	0.0591	0.2509	0.9463	0.6950	0.4275
517	SQNAVASKI	0.0686	0.2911	0.6703	0.7100	0.4271
161	RELHLSWEV	0.0637	0.2703	0.9382	0.3020	0.4261
60	VTQLYLGGM	0.0877	0.3722	0.2946	0.1740	0.4251
417	LEPEYFNSV	0.0637	0.2704	0.9688	0.1780	0.4246
155	REVLSDREL	0.0542	0.2299	0.8979	1.1880	0.4240
525	ILGLPTQTV	0.0618	0.2622	0.9781	0.2950	0.4237
283	PPGTGKSHF	0.0486	0.2064	0.9177	1.5570	0.4219
240	LVPQEHYVR	0.0558	0.2371	0.6856	1.6220	0.4210
403	AQLPAPRTL	0.0521	0.2214	0.9637	1.0470	0.4183
239	TLVPQEHYV	0.0612	0.2596	0.9645	0.2460	0.4166
181	VFTGYRVTK	0.0576	0.2445	0.9377	0.6060	0.4155
582	YDKLQFTSL	0.0554	0.2354	0.9716	0.6730	0.4148
389	ARLRAKHYV	0.0559	0.2373	0.9057	0.8240	0.4144
268	NYQKVGMQK	0.0525	0.2227	0.9745	0.8880	0.4133
34	VISTSHKLV	0.0814	0.3456	0.2730	0.4780	0.4105
513	SPYNSQNAV	0.0587	0.2494	0.9718	0.2590	0.4081
143	ETFKLSYGI	0.0677	0.2874	0.7096	0.2540	0.4065
352	LEQYVFCTV	0.0602	0.2557	0.9509	0.1610	0.4064
420	EYFNSVCRL	0.0498	0.2113	0.9704	0.9820	0.4060
244	EHYVRITGL	0.0511	0.2169	0.9448	0.8960	0.4034
321	ALKYLPIDK	0.0561	0.2381	0.8445	0.7270	0.4011
287	GKSHFAIGL	0.0530	0.2248	0.8983	0.8310	0.4011
406	PAPRTLLTK	0.0612	0.2599	0.9156	0.0700	0.4008
98	VGSDNVTDF	0.0549	0.2332	0.3423	2.3190	0.4005
455	LVYDNKLKA	0.0623	0.2646	0.9095	-0.0520	0.3985
397	VYIGDPAQL	0.0452	0.1919	0.9383	1.2980	0.3976

55	CDVTDVTQL	0.0531	0.2256	0.8974	0.6540	0.3929
324	YLPIDKCSR	0.0612	0.2598	0.3594	1.5700	0.3922
20	IRRPFLCCK	0.0503	0.2134	0.9355	0.7360	0.3905
592	IPRRNVATL	0.0506	0.2148	0.9786	0.5550	0.3893
233	MPLSAPTLV	0.0660	0.2801	0.6872	0.1220	0.3893
454	ALVYDNKLK	0.0560	0.2377	0.7522	0.7600	0.3885
428	LMKTIGPDM	0.0627	0.2663	0.6261	0.5600	0.3882
444	CPAEIVDTV	0.0638	0.2707	0.7423	0.1180	0.3880
577	SDRDLYDKL	0.0483	0.2050	0.9536	0.7850	0.3873
589	SLEIPRRNV	0.0604	0.2567	0.7850	0.2530	0.3871
149	YGIATVREV	0.0699	0.2967	0.5498	0.1510	0.3868
86	NGQVFGLYK	0.0615	0.2612	0.7168	0.3570	0.3865
363	LPETTADIV	0.0639	0.2711	0.7569	0.0240	0.3859
241	VPQEHYVRI	0.0559	0.2373	0.8810	0.3080	0.3849
210	VYRGTTTYK	0.0471	0.2000	0.9335	0.8870	0.3844
499	FLTRNPAWR	0.0615	0.2611	0.4159	1.2060	0.3838
159	SDRELHLSW	0.0470	0.1994	0.9761	0.7270	0.3821
168	EVGKPRPPL	0.0549	0.2330	0.7609	0.6700	0.3807
273	GMQKYSTLQ	0.0763	0.3238	0.4280	-0.1480	0.3806
44	SVNPYVCNA	0.0693	0.2941	0.6971	-0.3900	0.3792
125	TCTERLKLF	0.0510	0.2164	0.1965	2.6390	0.3778
297	LYYPSARIV	0.0489	0.2077	0.8226	0.9240	0.3773
219	LNVGDYFVL	0.0500	0.2125	0.7660	0.9850	0.3766
7	LCNSQTSLR	0.0601	0.2551	0.3079	1.4620	0.3744
319	EKALKYLPI	0.0591	0.2507	0.6833	0.3900	0.3727
370	IVVFDEISM	0.0511	0.2168	0.8934	0.4050	0.3711
414	KGTLEPEYF	0.0465	0.1973	0.3672	2.3490	0.3699
173	RPPLNRNYV	0.0542	0.2299	0.8928	0.0940	0.3685
280	LQGPPGTGK	0.0541	0.2299	0.7850	0.3910	0.3672
187	VTKNSKVQI	0.0635	0.2697	0.4139	0.7050	0.3671
179	NYVFTGYRV	0.0485	0.2061	0.7945	0.8260	0.3666
178	RNYVFTGYR	0.0588	0.2495	0.1400	1.9040	0.3657
474	MFYKGVITH	0.0607	0.2579	0.7230	-0.0330	0.3647
81	FPLCANGQV	0.0636	0.2702	0.6308	-0.0130	0.3642
340	VECFDKFKV	0.0538	0.2284	0.7767	0.3820	0.3640
14	LRCGACIRR	0.0576	0.2448	0.2035	1.7580	0.3632
52	APGCDVTDV	0.0545	0.2315	0.8273	0.1320	0.3622
304	IVYTACSHA	0.0618	0.2623	0.6835	-0.0550	0.3621
102	NVTDFNAIA	0.0700	0.2970	0.6117	-0.5350	0.3620
485	SSAINRPQI	0.0728	0.3093	0.1194	0.6920	0.3618
339	RVECFDKFK	0.0697	0.2959	0.1454	0.8570	0.3605

11	QTSLRCGAC	0.0832	0.3534	0.0264	0.0560	0.3601
23	PFLCCKCCY	0.0451	0.1916	0.2020	2.7620	0.3600
377	SMATNYDLS	0.1079	0.4583	0.0234	-2.0450	0.3595
537	QGSEYDYVI	0.0580	0.2464	0.6430	0.3310	0.3594
164	HLSWEVGKP	0.0796	0.3381	0.1045	0.0770	0.3576
488	INRPQIGVV	0.0505	0.2143	0.8257	0.3880	0.3575
382	YDLSVVNAR	0.0626	0.2659	0.1721	1.3160	0.3575
68	MSYYCKSHK	0.0726	0.3082	0.0829	0.7090	0.3561
13	SLRCGACIR	0.0572	0.2427	0.2078	1.6160	0.3546
568	AKVGILCIM	0.0562	0.2386	0.5300	0.7080	0.3535
331	SRIIPARAR	0.0513	0.2179	0.2988	1.8060	0.3531
562	NVAITRAKV	0.0687	0.2918	0.2680	0.4020	0.3521
405	LPAPRTLLT	0.0629	0.2669	0.9060	-1.0380	0.3509
371	VVFDEISMA	0.0605	0.2567	0.6943	-0.2140	0.3502
487	AINRPQIGV	0.0656	0.2784	0.2927	0.5470	0.3497
424	SVCRLMKTI	0.0644	0.2734	0.2172	0.8760	0.3497
422	FNSVCRLMK	0.0719	0.3055	0.2363	0.1610	0.3490
204	DYGDAVVYR	0.0478	0.2028	0.5504	1.2400	0.3474
498	EFLTRNPAW	0.0457	0.1942	0.6863	0.9980	0.3470
579	RDLYDKLQF	0.0457	0.1940	0.2354	2.3500	0.3468
401	DPAQLPAPR	0.0493	0.2094	0.6287	0.8510	0.3463
419	PEYFNSVCR	0.0460	0.1954	0.7039	0.8890	0.3454
325	LPIDKCSRI	0.0533	0.2263	0.7093	0.2510	0.3453
571	GILCIMSDR	0.0572	0.2429	0.1817	1.4990	0.3451
518	QNAVASKIL	0.0541	0.2296	0.4708	0.8860	0.3445
457	YDNKLKAHK	0.0624	0.2648	0.4946	0.0900	0.3435
492	QIGVVREFL	0.0668	0.2837	0.1317	0.7880	0.3428
288	KSHFAIGLA	0.0798	0.3388	0.1559	-0.3880	0.3428
17	GACIRRPFL	0.0550	0.2337	0.4440	0.8300	0.3418
552	TAHSCNVNR	0.0624	0.2648	0.0532	1.3790	0.3417
16	CGACIRRPF	0.0491	0.2083	0.0963	2.3760	0.3415
329	KCSRIIPAR	0.0545	0.2313	0.2354	1.4690	0.3401
421	YFNSVCRLM	0.0636	0.2702	0.3916	0.1910	0.3385
101	DNVTDFNAI	0.0510	0.2164	0.7431	0.1980	0.3377
411	LLTKGTLEP	0.0731	0.3102	0.1461	0.1020	0.3372
384	LSVVNARLR	0.0604	0.2565	0.0425	1.4680	0.3362
536	SQGSEYDYV	0.0700	0.2972	0.1257	0.3340	0.3328
381	NYDLSVVNA	0.0572	0.2429	0.7314	-0.4000	0.3326
106	FNAIATCDW	0.0567	0.2408	0.4375	0.5150	0.3322
264	SNVANYQKV	0.0579	0.2458	0.4461	0.3470	0.3301
368	ADIVVFDEI	0.0591	0.2508	0.3723	0.4500	0.3292

521	VASKILGLP	0.0719	0.3054	0.0346	0.3690	0.3291
32	DHVISTSHK	0.0486	0.2062	0.7565	0.1820	0.3288
165	LSWEVGKPR	0.0519	0.2202	0.1633	1.6780	0.3286
299	YPSARIVYT	0.0641	0.2721	0.7366	-1.0860	0.3283
335	PARARVECF	0.0486	0.2065	0.0899	2.1650	0.3282
544	VIFTQTTET	0.0667	0.2833	0.4483	-0.4550	0.3278
226	VLTSHTVMP	0.0677	0.2876	0.2469	0.0450	0.3268
300	PSARIVYTA	0.0680	0.2888	0.5842	-1.0100	0.3259
350	STLEQYVFC	0.0734	0.3114	0.0271	0.2050	0.3258
548	QTTETAHSC	0.0757	0.3216	0.0321	-0.0200	0.3254
348	VNSTLEQYV	0.0605	0.2568	0.3502	0.3210	0.3254
315	DALCEKALK	0.0596	0.2529	0.3770	0.2660	0.3227
398	YIGDPAQLP	0.0730	0.3100	0.0444	0.0970	0.3215
351	TLEQYVFCT	0.0808	0.3430	0.1220	-0.7970	0.3214
317	LCEKALKYL	0.0533	0.2261	0.2999	0.9890	0.3206
295	LALYYPSAR	0.0563	0.2390	0.0329	1.5290	0.3204
64	YLGGMSYYC	0.0734	0.3116	0.0897	-0.1010	0.3200
246	YVRITGLYP	0.0711	0.3018	0.0283	0.2490	0.3185
27	CKCCYDHVI	0.0586	0.2489	0.2798	0.5450	0.3181
506	WRKAVFISP	0.0557	0.2364	0.3904	0.4360	0.3168
586	QFTSLEIPR	0.0521	0.2211	0.0707	1.6770	0.3155
386	VVNARLRAK	0.0581	0.2465	0.2393	0.6250	0.3137
313	AVDALCEKA	0.0717	0.3043	0.1286	-0.2750	0.3099
385	SVVNARLRA	0.0709	0.3011	0.1140	-0.2180	0.3073
469	AQCFKMFYK	0.0609	0.2584	0.0740	0.7470	0.3068
367	TADIVVFDE	0.0914	0.3882	0.0231	-1.7000	0.3066
31	YDHVISTSH	0.0608	0.2581	0.6015	-0.8400	0.3063
163	LHLSWEVGK	0.0536	0.2277	0.4139	0.3270	0.3062
337	RARVECFDK	0.0560	0.2376	0.1316	0.9640	0.3056
170	GKPRPPLNR	0.0494	0.2099	0.1749	1.3870	0.3055
202	KGDYGDAVV	0.0565	0.2398	0.4990	-0.1880	0.3053
294	GLALYYPSA	0.0618	0.2625	0.5056	-0.6690	0.3049
564	AITRAKVGI	0.0596	0.2532	0.0629	0.8180	0.3035
482	HDVSSAINR	0.0566	0.2403	0.0292	1.1740	0.3033
138	LKATEETFK	0.0612	0.2599	0.1050	0.5450	0.3029
108	AIATCDWTN	0.0804	0.3415	0.0291	-0.8850	0.3017
12	TSLRCGACI	0.0612	0.2599	0.1022	0.5230	0.3014
539	SEYDYVIFT	0.0625	0.2656	0.4439	-0.6310	0.3006
566	TRAKVGILC	0.0665	0.2823	0.0346	0.2260	0.2988
573	LCIMSDRDL	0.0544	0.2311	0.0973	1.0570	0.2986
511	FISPYNSQN	0.0811	0.3444	0.1072	-1.2390	0.2986

484	VSSAINRPQ	0.0710	0.3013	0.0284	-0.1460	0.2983
466	DKSAQCFKM	0.0549	0.2330	0.3875	0.1350	0.2979
220	NVGDYFVLT	0.0705	0.2991	0.1968	-0.6450	0.2964
472	FKMFYKGVI	0.0588	0.2499	0.1265	0.5470	0.2962
346	FKVNSTLEQ	0.0680	0.2887	0.0286	0.0280	0.2944
396	YVYIGDPAQ	0.0622	0.2641	0.1416	0.1770	0.2942
540	EYDYVIFTQ	0.0575	0.2440	0.3879	-0.1670	0.2938
550	TETAHSCNV	0.0586	0.2486	0.2535	0.1320	0.2933
266	VANYQKVGM	0.0552	0.2343	0.2168	0.4660	0.2901
483	DVSSAINRP	0.0672	0.2851	0.0603	-0.1000	0.2892
555	SCNVNRFNV	0.0607	0.2577	0.1112	0.2710	0.2879
326	PIDKCSRII	0.0636	0.2700	0.0974	0.0550	0.2873
504	PAWRKAVFI	0.0544	0.2308	0.2292	0.4200	0.2862
459	NKLKAHKDK	0.0529	0.2244	0.2592	0.4300	0.2848
441	CRRCPAEIV	0.0518	0.2198	0.2535	0.5390	0.2847
36	STSHKLVLS	0.0920	0.3904	0.0286	-2.2090	0.2843
356	VFCTVNALP	0.0618	0.2622	0.0485	0.2910	0.2841
235	LSAPTLVPQ	0.0671	0.2850	0.0543	-0.1890	0.2837
529	PTQTVDSSQ	0.0715	0.3036	0.0625	-0.5910	0.2834
502	RNPAWRKAV	0.0549	0.2331	0.2015	0.4010	0.2834
593	PRRNVATLQ	0.0495	0.2101	0.5911	-0.3210	0.2828
228	TSHTVMPLS	0.0903	0.3835	0.0384	-2.1480	0.2819
250	TGLYPTLNI	0.0527	0.2239	0.2860	0.2740	0.2805
21	RRPFLCCKC	0.0520	0.2206	0.2364	0.4590	0.2790
69	SYYCKSHKP	0.0492	0.2088	0.2460	0.6450	0.2780
49	VCNAPGCDV	0.0596	0.2532	0.0814	0.2510	0.2780
90	FGLYKNTCV	0.0569	0.2414	0.2366	0.0180	0.2778
554	HSCNVNRFN	0.0787	0.3341	0.0232	-1.2030	0.2774
561	FNVAITRAK	0.0565	0.2398	0.0958	0.4270	0.2755
523	SKILGLPTQ	0.0536	0.2278	0.2444	0.2200	0.2754
237	APTLVPQEH	0.0530	0.2251	0.5613	-0.6780	0.2754
394	KHYVYIGDP	0.0559	0.2375	0.0648	0.5600	0.2753
542	DYVIFTQTT	0.0476	0.2023	0.7286	-0.7310	0.2750
314	VDALCEKAL	0.0476	0.2019	0.1919	0.8800	0.2747
71	YCKSHKPPI	0.0540	0.2292	0.1428	0.4810	0.2746
532	TVDSSQGSE	0.0835	0.3547	0.0234	-1.6730	0.2746
132	LFAAETLKA	0.0620	0.2634	0.1505	-0.2460	0.2737
433	GPDMFLGTC	0.0575	0.2442	0.3970	-0.6360	0.2720
399	IGDPAQLPA	0.0614	0.2608	0.4037	-1.0070	0.2710
129	RLKLFAAET	0.0614	0.2607	0.2721	-0.6110	0.2710
140	ATEETFKLS	0.0884	0.3755	0.0244	-2.1660	0.2709

512	ISPYNSQNA	0.0581	0.2468	0.3554	-0.5920	0.2705
180	YVFTGYRVT	0.0626	0.2658	0.1412	-0.3340	0.2702
560	RFNVAITRA	0.0579	0.2458	0.1845	-0.0770	0.2697
8	CNSQTSLRC	0.0637	0.2705	0.0373	-0.1350	0.2693
136	ETLKATEET	0.0707	0.3000	0.0481	-0.7590	0.2692
366	TTADIVVFD	0.0854	0.3628	0.0380	-2.0080	0.2681
230	HTVMPLSAP	0.0600	0.2548	0.0413	0.1240	0.2672
201	EKGDYGDAV	0.0600	0.2548	0.1188	-0.1080	0.2672
310	SHAAVDALC	0.0606	0.2571	0.0353	0.0850	0.2667
110	ATCDWTNAG	0.0760	0.3229	0.0373	-1.2550	0.2657
292	AIGLALYYP	0.0603	0.2559	0.0264	0.1090	0.2653
42	VLSVNPYVC	0.0581	0.2467	0.0746	0.1400	0.2649
541	YDYVIFTQT	0.0607	0.2576	0.3816	-1.0130	0.2642
545	IFTQTTETA	0.0517	0.2193	0.4103	-0.3340	0.2641
113	DWTNAGDYI	0.0509	0.2160	0.1802	0.4220	0.2641
585	LQFTSLEIP	0.0566	0.2402	0.0247	0.3970	0.2638
590	LEIPRRNVA	0.0507	0.2154	0.5012	-0.5440	0.2634
364	PETTADIVV	0.0548	0.2327	0.2980	-0.2890	0.2629
479	VITHDVSSA	0.0507	0.2153	0.4526	-0.4110	0.2626
275	QKYSTLQGP	0.0561	0.2384	0.0277	0.3900	0.2620
267	ANYQKVGMQ	0.0540	0.2294	0.1770	0.1200	0.2619
15	RCGACIRRP	0.0578	0.2453	0.0244	0.2540	0.2616
440	TCRRCPAEI	0.0534	0.2265	0.0431	0.5530	0.2606
123	ANTCTERLK	0.0521	0.2213	0.1050	0.4680	0.2605
18	ACIRRPFLC	0.0577	0.2450	0.0301	0.2180	0.2604
26	CCKCCYDHV	0.0549	0.2332	0.0575	0.3400	0.2588
522	ASKILGLPT	0.0641	0.2721	0.0640	-0.4760	0.2579
249	ITGLYPTLN	0.0743	0.3155	0.0563	-1.3450	0.2567
231	TVMPLSAPT	0.0599	0.2545	0.1937	-0.5460	0.2563
578	DRDLYDKLQ	0.0511	0.2170	0.3701	-0.3370	0.2557
65	LGGMSYYCK	0.0512	0.2173	0.2593	-0.0130	0.2556
434	PDMFLGTCR	0.0457	0.1940	0.1648	0.7320	0.2554
276	KYSTLQGPP	0.0530	0.2251	0.0583	0.4230	0.2550
19	CIRRPFLCC	0.0530	0.2252	0.0953	0.3050	0.2548
322	LKYLPIDKC	0.0539	0.2287	0.0316	0.4090	0.2539
358	CTVNALPET	0.0681	0.2890	0.0300	-0.7950	0.2537
184	GYRVTKNSK	0.0424	0.1799	0.2797	0.6090	0.2523
395	HYVYIGDPA	0.0498	0.2117	0.3285	-0.1830	0.2518
10	SQTSLRCGA	0.0593	0.2518	0.0965	-0.3020	0.2511
524	KILGLPTQT	0.0547	0.2324	0.2526	-0.3980	0.2504
39	HKLVLSVNP	0.0559	0.2374	0.0406	0.1350	0.2502

70	YYCKSHKPP	0.0530	0.2249	0.0275	0.4120	0.2496
372	VFDEISMAT	0.0617	0.2619	0.1114	-0.6050	0.2484
426	CRLMKTIGP	0.0533	0.2263	0.0316	0.3350	0.2478
76	KPPISFPLC	0.0503	0.2137	0.2948	-0.2210	0.2469
437	FLGTCRRCP	0.0585	0.2483	0.0228	-0.0980	0.2468
303	RIVYTACSH	0.0552	0.2345	0.0525	0.0690	0.2458
465	KDKSAQCFK	0.0509	0.2163	0.1145	0.2390	0.2454
205	YGDAVVYRG	0.0791	0.3360	0.0557	-1.9970	0.2445
104	TDFNAIATC	0.0563	0.2392	0.0832	-0.1550	0.2439
234	PLSAPTLVP	0.0565	0.2397	0.1328	-0.3240	0.2434
222	GDYFVLTSH	0.0468	0.1986	0.5597	-0.7880	0.2432
471	CFKMFYKGV	0.0487	0.2068	0.1095	0.3900	0.2427
334	IPARARVEC	0.0589	0.2499	0.0298	-0.2410	0.2424
115	TNAGDYILA	0.0591	0.2509	0.1835	-0.7480	0.2411
591	EIPRRNVAT	0.0583	0.2475	0.2018	-0.7480	0.2404
116	NAGDYILAN	0.0703	0.2985	0.0724	-1.3790	0.2404
145	FKLSYGIAT	0.0639	0.2712	0.0299	-0.7230	0.2395
360	VNALPETTA	0.0528	0.2243	0.2994	-0.5990	0.2392
359	TVNALPETT	0.0634	0.2693	0.0240	-0.6890	0.2385
198	YTFEKGDYG	0.0726	0.3082	0.0273	-1.4920	0.2377
89	VFGLYKNTC	0.0545	0.2313	0.0291	0.0320	0.2372
301	SARIVYTAC	0.0540	0.2293	0.0426	0.0240	0.2369
247	VRITGLYPT	0.0569	0.2417	0.1051	-0.4200	0.2365
510	VFISPYNSQ	0.0492	0.2090	0.0648	0.3470	0.2361
77	PPISFPLCA	0.0516	0.2191	0.4835	-1.1500	0.2341
182	FTGYRVTKN	0.0712	0.3021	0.0601	-1.5480	0.2337
443	RCPAEIVDT	0.0574	0.2437	0.0331	-0.3240	0.2325
166	SWEVGKPRP	0.0502	0.2132	0.0245	0.3080	0.2323
208	AVVYRGTTT	0.0590	0.2504	0.0397	-0.4840	0.2322
446	AEIVDTVSA	0.0535	0.2270	0.2354	-0.6030	0.2321
133	FAAETLKAT	0.0613	0.2601	0.0299	-0.6830	0.2304
462	KAHKDKSAQ	0.0515	0.2187	0.0326	0.1320	0.2302
330	CSRIIPARA	0.0555	0.2356	0.0905	-0.3860	0.2299
432	IGPDMFLGT	0.0571	0.2424	0.2323	-0.9540	0.2296
436	MFLGTCRRC	0.0515	0.2187	0.0244	0.1080	0.2278
186	RVTKNSKVQ	0.0499	0.2119	0.0409	0.1890	0.2274
423	NSVCRLMKT	0.0602	0.2555	0.0305	-0.6550	0.2273
29	CCYDHVIST	0.0569	0.2416	0.0490	-0.4380	0.2271
156	EVLSDRELH	0.0569	0.2415	0.0507	-0.4510	0.2265
109	IATCDWTNA	0.0561	0.2382	0.0346	-0.3420	0.2263
80	SFPLCANGQ	0.0509	0.2162	0.0249	0.1090	0.2254

191	SKVQIGEYT	0.0556	0.2362	0.0515	-0.3840	0.2247
305	VYTACSHAA	0.0492	0.2089	0.1775	-0.2360	0.2238
308	ACSHAAVDA	0.0554	0.2352	0.0443	-0.3650	0.2236
3	GACVLCNSQ	0.0535	0.2272	0.0333	-0.1760	0.2234
318	CEKALKYLP	0.0521	0.2213	0.0228	-0.0260	0.2234
278	STLQGPPGT	0.0575	0.2442	0.0963	-0.7080	0.2233
497	REFLTRNPA	0.0507	0.2152	0.1414	-0.2680	0.2230
501	TRNPAWRKA	0.0505	0.2144	0.1857	-0.3960	0.2225
25	LCCKCCYDH	0.0584	0.2482	0.0299	-0.6070	0.2223
473	KMFYKGVIT	0.0563	0.2390	0.0432	-0.4700	0.2220
496	VREFLTRNP	0.0480	0.2037	0.0266	0.2760	0.2215
451	TVSALVYDN	0.0668	0.2838	0.0307	-1.3410	0.2214
54	GCDVTDVTQ	0.0573	0.2434	0.0375	-0.5850	0.2198
107	NAIATCDWT	0.0586	0.2487	0.0225	-0.6520	0.2195
45	VNPYVCNAP	0.0499	0.2120	0.0304	0.0530	0.2192
120	YILANTCTE	0.0684	0.2903	0.0484	-1.5780	0.2186
88	QVFGLYKNT	0.0511	0.2169	0.1675	-0.4720	0.2185
354	QYVFCTVNA	0.0474	0.2014	0.2290	-0.3470	0.2184
207	DAVVYRGTT	0.0606	0.2573	0.0419	-0.9210	0.2176
580	DLYDKLQFT	0.0557	0.2363	0.0963	-0.6830	0.2166
229	SHTVMPLSA	0.0535	0.2273	0.0889	-0.5010	0.2156
311	HAAVDALCE	0.0679	0.2882	0.0234	-1.5330	0.2151
575	IMSDRDLYD	0.0679	0.2885	0.0670	-1.6820	0.2144
74	SHKPPISFP	0.0447	0.1898	0.0755	0.2640	0.2143
167	WEVGKPRPP	0.0486	0.2065	0.0240	0.0740	0.2138
67	GMSYYCKSH	0.0534	0.2266	0.1205	-0.6270	0.2133
556	CNVNRFNVA	0.0532	0.2259	0.1132	-0.5970	0.2130
1	AVGACVLCN	0.0634	0.2690	0.0255	-1.1980	0.2129
387	VNARLRAKH	0.0556	0.2360	0.0296	-0.5620	0.2123
4	ACVLCNSQT	0.0543	0.2308	0.0408	-0.4930	0.2122
118	GDYILANTC	0.0498	0.2113	0.0495	-0.1310	0.2121
221	VGDYFVLTS	0.0767	0.3257	0.0652	-2.4850	0.2112
328	DKCSRIIPA	0.0560	0.2379	0.0783	-0.7840	0.2104
277	YSTLQGPPG	0.0636	0.2701	0.0362	-1.3100	0.2100
259	SDEFSSNVA	0.0491	0.2083	0.3158	-0.9250	0.2094
463	AHKDKSAQC	0.0447	0.1899	0.0319	0.2650	0.2079
410	TLLTKGTLE	0.0646	0.2744	0.0276	-1.4170	0.2077
456	VYDNKLKAH	0.0482	0.2047	0.1296	-0.3370	0.2073
257	NISDEFSSN	0.0603	0.2560	0.0410	-1.1060	0.2069
271	KVGMQKYST	0.0538	0.2286	0.0775	-0.6720	0.2067
543	YVIFTQTTE	0.0642	0.2726	0.0654	-1.5170	0.2065

392	RAKHYVYIG	0.0502	0.2132	0.3057	-1.0530	0.2064
22	RPFLCCKCC	0.0452	0.1918	0.0288	0.1980	0.2060
93	YKNTCVGSD	0.0689	0.2927	0.0249	-1.8320	0.2049
427	RLMKTIGPD	0.0645	0.2737	0.0378	-1.4940	0.2046
470	QCFKMFYKG	0.0582	0.2470	0.0714	-1.0960	0.2029
127	TERLKLFAA	0.0502	0.2130	0.1483	-0.6500	0.2028
223	DYFVLTSHT	0.0461	0.1958	0.2684	-0.6750	0.2023
48	YVCNAPGCD	0.0683	0.2901	0.0246	-1.8330	0.2021
527	GLPTQTVDS	0.0622	0.2639	0.3715	-2.3720	0.2010
407	APRTLLTKG	0.0532	0.2258	0.3854	-1.6520	0.2010
431	TIGPDMFLG	0.0607	0.2578	0.0685	-1.3500	0.2006
495	VVREFLTRN	0.0586	0.2488	0.0367	-1.0960	0.1995
514	PYNSQNAVA	0.0435	0.1848	0.3588	-0.7910	0.1991
96	TCVGSDNVT	0.0514	0.2184	0.0315	-0.4870	0.1988
486	SAINRPQIG	0.0594	0.2523	0.0271	-1.1670	0.1980
100	SDNVTDFNA	0.0533	0.2264	0.1105	-0.9140	0.1973
418	EPEYFNSVC	0.0497	0.2108	0.1059	-0.5900	0.1972
215	TTYKLNVGD	0.0644	0.2734	0.0337	-1.6610	0.1954
282	GPPGTGKSH	0.0430	0.1824	0.4354	-1.0830	0.1936
279	TLQGPPGTG	0.0568	0.2410	0.1367	-1.3750	0.1927
117	AGDYILANT	0.0545	0.2312	0.0302	-0.8630	0.1926
400	GDPAQLPAP	0.0485	0.2060	0.0343	-0.3880	0.1917
152	ATVREVLSD	0.0619	0.2630	0.0579	-1.6060	0.1914
200	FEKGDYGDA	0.0518	0.2198	0.0565	-0.7580	0.1904
353	EQYVFCTVN	0.0522	0.2216	0.1840	-1.2090	0.1887
144	TFKLSYGIA	0.0490	0.2080	0.0555	-0.5580	0.1885
111	TCDWTNAGD	0.0649	0.2754	0.0266	-1.8380	0.1875
119	DYILANTCT	0.0484	0.2057	0.1169	-0.7180	0.1873
265	NVANYQKVG	0.0566	0.2402	0.0289	-1.1490	0.1871
563	VAITRAKVG	0.0556	0.2361	0.0284	-1.0710	0.1868
551	ETAHSCNVN	0.0610	0.2590	0.0236	-1.5140	0.1868
581	LYDKLQFTS	0.0674	0.2860	0.1011	-2.2920	0.1866
252	LYPTLNISD	0.0539	0.2288	0.2493	-1.5920	0.1866
134	AAETLKATE	0.0617	0.2621	0.0315	-1.6180	0.1859
327	IDKCSRIIP	0.0466	0.1979	0.0260	-0.3270	0.1854
416	TLEPEYFNS	0.0705	0.2995	0.0456	-2.4210	0.1853
47	PYVCNAPGC	0.0424	0.1802	0.0259	0.0180	0.1850
531	QTVDSSQGS	0.0679	0.2882	0.0309	-2.1660	0.1846
214	TTTYKLNVG	0.0601	0.2554	0.0250	-1.5040	0.1839
519	NAVASKILG	0.0574	0.2437	0.0256	-1.2770	0.1837
236	SAPTLVPQE	0.0585	0.2483	0.0416	-1.4270	0.1832

475	FYKGVITHD	0.0482	0.2046	0.3932	-1.6120	0.1830
461	LKAHKDKSA	0.0451	0.1913	0.0644	-0.3640	0.1828
91	GLYKNTCVG	0.0551	0.2338	0.1292	-1.4730	0.1796
493	IGVVREFLT	0.0520	0.2209	0.0282	-0.9200	0.1791
162	ELHLSWEVG	0.0552	0.2343	0.0882	-1.4180	0.1766
50	CNAPGCDVT	0.0503	0.2135	0.0303	-0.8350	0.1763
508	KAVFISPYN	0.0510	0.2165	0.0430	-0.9460	0.1756
43	LSVNPYVCN	0.0528	0.2244	0.0457	-1.1460	0.1739
175	PLNRNYVFT	0.0564	0.2395	0.0288	-1.4050	0.1736
97	CVGSDNVTD	0.0616	0.2614	0.0255	-1.8710	0.1716
5	CVLCNSQTS	0.0610	0.2591	0.0745	-1.9760	0.1715
302	ARIVYTACS	0.0552	0.2343	0.1691	-1.7720	0.1710
30	CYDHVISTS	0.0622	0.2639	0.1149	-2.2050	0.1709
558	VNRFNVAIT	0.0463	0.1964	0.0295	-0.6060	0.1705
438	LGTCRRCPA	0.0494	0.2096	0.0471	-0.9390	0.1697
9	NSQTSLRCG	0.0548	0.2326	0.0275	-1.3570	0.1688
83	LCANGQVFG	0.0563	0.2390	0.0237	-1.4990	0.1676
588	TSLEIPRRN	0.0543	0.2304	0.0378	-1.3830	0.1669
274	MQKYSTLQG	0.0536	0.2274	0.0276	-1.3050	0.1663
380	TNYDLSVVN	0.0510	0.2166	0.0458	-1.1480	0.1660
59	DVTQLYLGG	0.0569	0.2417	0.0257	-1.5990	0.1656
176	LNRNYVFTG	0.0561	0.2381	0.0609	-1.6400	0.1652
572	ILCIMSDRD	0.0589	0.2501	0.0244	-1.7940	0.1640
307	TACSHAAVD	0.0590	0.2504	0.0313	-1.8270	0.1638
193	VQIGEYTFE	0.0523	0.2221	0.0281	-1.2580	0.1634
402	PAQLPAPRT	0.0513	0.2178	0.0618	-1.3040	0.1618
58	TDVTQLYLG	0.0558	0.2370	0.0302	-1.5990	0.1616
569	KVGILCIMS	0.0600	0.2546	0.0460	-2.0090	0.1610
46	NPYVCNAPG	0.0511	0.2168	0.1041	-1.4290	0.1610
51	NAPGCDVTD	0.0571	0.2426	0.0327	-1.7480	0.1601
357	FCTVNALPE	0.0561	0.2381	0.0230	-1.6290	0.1601
320	KALKYLPID	0.0562	0.2387	0.0760	-1.8050	0.1599
442	RRCPAEIVD	0.0526	0.2234	0.0278	-1.3630	0.1594
243	QEHYVRITG	0.0555	0.2356	0.0354	-1.6330	0.1592
478	GVITHDVSS	0.0608	0.2582	0.0442	-2.1160	0.1591
336	ARARVECFD	0.0549	0.2330	0.0365	-1.5930	0.1588
188	TKNSKVQIG	0.0526	0.2231	0.0407	-1.4140	0.1585
373	FDEISMATN	0.0578	0.2455	0.0236	-1.8150	0.1583
515	YNSQNAVAS	0.0661	0.2807	0.0297	-2.5670	0.1568
154	VREVLSDRE	0.0528	0.2240	0.0247	-1.4310	0.1562
53	PGCDVTDVT	0.0483	0.2051	0.1020	-1.2890	0.1559

490	RPQIGVVRE	0.0487	0.2068	0.1635	-1.5170	0.1555
534	DSSQGSEYD	0.0598	0.2540	0.0245	-2.0480	0.1552
24	FLCCKCCYD	0.0596	0.2529	0.0247	-2.0270	0.1552
135	AETLKATEE	0.0513	0.2178	0.0592	-1.4390	0.1547
148	SYGIATVRE	0.0485	0.2060	0.1109	-1.3630	0.1545
481	THDVSSAIN	0.0531	0.2252	0.0261	-1.4960	0.1544
189	KNSKVQIGE	0.0542	0.2299	0.0281	-1.6050	0.1539
393	AKHYVYIGD	0.0523	0.2221	0.0290	-1.4600	0.1535
425	VCRLMKTIG	0.0496	0.2105	0.0227	-1.2100	0.1534
94	KNTCVGSDN	0.0522	0.2216	0.0273	-1.4490	0.1533
87	GQVFGLYKN	0.0476	0.2021	0.0701	-1.2000	0.1526
333	IIPARARVE	0.0502	0.2133	0.0275	-1.2970	0.1525
450	DTVSALVYD	0.0588	0.2495	0.0286	-2.0280	0.1524
212	RGTTTYKLN	0.0517	0.2196	0.0245	-1.4260	0.1520
79	ISFPLCANG	0.0494	0.2099	0.0685	-1.3650	0.1519
255	TLNISDEFS	0.0641	0.2723	0.0232	-2.4860	0.1515
206	GDAVVYRGT	0.0493	0.2091	0.0234	-1.2270	0.1513
251	GLYPTLNIS	0.0539	0.2287	0.2152	-2.2080	0.1506
253	YPTLNISDE	0.0553	0.2348	0.0626	-1.8780	0.1503
408	PRTLLTKGT	0.0437	0.1854	0.0503	-0.8610	0.1499
415	GTLEPEYFN	0.0503	0.2135	0.0330	-1.3970	0.1486
361	NALPETTAD	0.0531	0.2255	0.0370	-1.6610	0.1480
439	GTCRRCPAE	0.0540	0.2293	0.0228	-1.7090	0.1473
343	FDKFKVNST	0.0464	0.1972	0.0361	-1.1170	0.1468
38	SHKLVLSVN	0.0478	0.2029	0.0376	-1.2470	0.1462
570	VGILCIMSD	0.0552	0.2345	0.0251	-1.8500	0.1458
61	TQLYLGGMS	0.0583	0.2477	0.0290	-2.1260	0.1457
530	TQTVDSSQG	0.0511	0.2172	0.0295	-1.5220	0.1455
547	TQTTETAHS	0.0601	0.2553	0.0439	-2.3320	0.1453
412	LTKGTLEPE	0.0513	0.2178	0.0248	-1.5370	0.1446
375	EISMATNYD	0.0571	0.2422	0.0264	-2.0340	0.1445
342	CFDKFKVNS	0.0620	0.2632	0.0259	-2.4620	0.1440
128	ERLKLFAAE	0.0501	0.2128	0.0455	-1.5350	0.1429
509	AVFISPYNS	0.0518	0.2201	0.0568	-1.7500	0.1411
260	DEFSSNVAN	0.0511	0.2170	0.0272	-1.6010	0.1410
242	PQEHYVRIT	0.0464	0.1969	0.0273	-1.2070	0.1407
66	GGMSYYCKS	0.0569	0.2416	0.1826	-2.5780	0.1401
171	KPRPPLNRN	0.0457	0.1942	0.1284	-1.4940	0.1387
369	DIVVFDEIS	0.0569	0.2414	0.0261	-2.2010	0.1353
583	DKLQFTSLE	0.0526	0.2233	0.0265	-1.8660	0.1339
286	TGKSHFAIG	0.0503	0.2137	0.0310	-1.7150	0.1326

151	IATVREVLS	0.0562	0.2388	0.0253	-2.2360	0.1308
281	QGPPGTGKS	0.0555	0.2356	0.1211	-2.4660	0.1305
199	TFEKGDYGD	0.0515	0.2188	0.0279	-1.8650	0.1297
505	AWRKAVFIS	0.0523	0.2219	0.0256	-1.9510	0.1282
528	LPTQTVDSS	0.0501	0.2127	0.3052	-2.6120	0.1279
345	KFKVNSTLE	0.0456	0.1934	0.0344	-1.4320	0.1270
284	PGTGKSHFA	0.0443	0.1882	0.0778	-1.4630	0.1267
2	VGACVLCNS	0.0550	0.2334	0.0429	-2.2790	0.1259
460	KLKAHKDKS	0.0518	0.2201	0.0272	-1.9800	0.1252
195	IGEYTFEKG	0.0501	0.2126	0.0250	-1.8320	0.1247
341	ECFDKFKVN	0.0437	0.1856	0.0368	-1.3400	0.1241
526	LGLPTQTVD	0.0487	0.2068	0.1135	-2.0510	0.1212
196	GEYTFEKGD	0.0502	0.2131	0.0283	-1.9340	0.1207
78	PISFPLCAN	0.0481	0.2044	0.0277	-1.8160	0.1178
142	EETFKLSYG	0.0471	0.2000	0.0351	-1.7610	0.1172
160	DRELHLSWE	0.0467	0.1984	0.0355	-1.7650	0.1155
105	DFNAIATCD	0.0500	0.2121	0.0388	-2.0480	0.1155
256	LNISDEFSS	0.0529	0.2246	0.0241	-2.3150	0.1125
28	KCCYDHVIS	0.0503	0.2136	0.0231	-2.1160	0.1112
270	QKVGMQKYS	0.0503	0.2137	0.0343	-2.1570	0.1109
169	VGKPRPPLN	0.0440	0.1868	0.0275	-1.6890	0.1065
293	IGLALYYPS	0.0511	0.2168	0.0246	-2.3170	0.1047
92	LYKNTCVGS	0.0451	0.1915	0.0633	-2.0090	0.1005
183	TGYRVTKNS	0.0508	0.2158	0.0295	-2.4510	0.0976
445	PAEIVDTVS	0.0528	0.2241	0.0739	-2.8240	0.0940
323	KYLPIDKCS	0.0421	0.1786	0.0370	-1.8080	0.0938
458	DNKLKAHKD	0.0459	0.1948	0.0639	-2.3380	0.0874
477	KGVITHDVS	0.0472	0.2004	0.0277	-2.3790	0.0856
72	CKSHKPPIS	0.0455	0.1930	0.0232	-2.2930	0.0818

Table S5. HTL epitope of SARS-CoV-2 Main protease (Mpro) protein having strong binding affinity, predicted by NetMHC-II.

Alleles	Peptides	core Oflog50k (aff)	Binding level
DRB1_0101	KYKFVRIQPGQTFSV	0.9145	Strong binder
DRB1_0101	PKYKFVRIQPGQTFS	0.9009	Strong binder
DRB1_0101	YKFVRIQPGQTFSVL	0.9002	Strong binder
DRB1_0101	KFVRIQPGQTFSVLA	0.8791	Strong binder
DRB1_0101	TPKYKFVRIQPGQTF	0.8786	Strong binder
DRB1_0101	NHNFLVQAGNVQLRV	0.8528	Strong binder
DRB1_0101	HNFLVQAGNVQLRVI	0.8521	Strong binder
DRB1_0101	NFLVQAGNVQLRVIG	0.8299	Strong binder
DRB1_0101	SNHNFLVQAGNVQLR	0.8266	Strong binder
DRB1_0101	FVRIQPGQTFSVLAC	0.8181	Strong binder

Table S6. HTL epitope of SARS-CoV-2 RNA polymerase protein having strong binding affinity, predicted by NetMHC-II.

Alleles	peptides	core Of log50k (aff)	Binding level
DRB1_0101	PEFYEAMYTPHTVLQ	0.9077	Strong binder
DRB1_0101	SHRFYRLANECAQVL	0.9061	Strong binder
DRB1_0101	QKLLKSIAATRGATV	0.9055	Strong binder
DRB1_0101	HQKLLKSIAATRGAT	0.9041	Strong binder
DRB1_0101	HRFYRLANECAQVLS	0.9013	Strong binder
DRB1_0101	EPEFYEAMYTPHTVL	0.8989	Strong binder
DRB1_0101	FHQKLLKSIAATRGA	0.8974	Strong binder
DRB1_0101	LSHRFYRLANECAQV	0.8856	Strong binder
DRB1_0101	RFYRLANECAQVLSE	0.8856	Strong binder
DRB1_0101	KLLKSIAATRGATVV	0.8813	Strong binder
DRB1_0101	QFHQKLLKSIAATRG	0.8702	Strong binder
DRB1_0101	WEPEFYEAMYTPHTV	0.8643	Strong binder
DRB1_0101	PNMLRIMASLVLARK	0.8643	Strong binder
DRB1_0101	MPNMLRIMASLVLAR	0.8616	Strong binder
DRB1_0101	SLSHRFYRLANECAQ	0.8596	Strong binder
DRB1_0101	NMLRIMASLVLARKH	0.8539	Strong binder
DRB1_0101	RQFHQKLLKSIAATR	0.8511	Strong binder
DRB1_0101	AMPNMLRIMASLVLA	0.8347	Strong binder
DRB1_0101	LLKSIAATRGATVVI	0.8272	Strong binder
DRB1_0101	MLRIMASLVLARKHT	0.8255	Strong binder
DRB1_0101	FLNRVCGVSAARLTP	0.8249	Strong binder
DRB1_0101	LLVYAADPAMHAASG	0.8236	Strong binder
DRB1_0101	KGFFKEGSSVELKHF	0.8227	Strong binder
DRB1_0101	NRQFHQKLLKSIAAT	0.8223	Strong binder
DRB1_0101	FYAYLRKHFSMMILS	0.8205	Strong binder
DRB1_0101	SFLNRVCGVSAARLT	0.8204	Strong binder
DRB1_0101	FYRLANECAQVLSEM	0.8181	Strong binder
DRB1_0101	ELLVYAADPAMHAAS	0.8163	Strong binder
DRB1_0101	EFYAYLRKHFSMMIL	0.8135	Strong binder
DRB1_0101	LTGHMLDMYSVMLTN	0.8101	Strong binder
DRB1_0101	PDILRVYANLGERVR	0.8087	Strong binder
DRB1_0101	SKGFFKEGSSVELKH	0.8058	Strong binder
DRB1_0101	NPDILRVYANLGERV	0.8054	Strong binder
DRB1_0101	LNRVCGVSAARLTPC	0.8036	Strong binder
DRB1_0101	CSLSHRFYRLANECA	0.8035	Strong binder
DRB1_0101	TGHMLDMYSVMLTND	0.8028	Strong binder

Table S7. HTL epitope of SARS-CoV-2 Nsp13 helicase having strong binding affinity, predicted by NetMHC-I.

Alleles	peptides	core Of log50k (aff)	Binding level
DRB1_0101	ASKILGLPTQTVDSS	0.8936	Strong binder
DRB1_0101	VASKILGLPTQTVDS	0.8853	Strong binder
DRB1_0101	SKILGLPTQTVDSSQ	0.8782	Strong binder
DRB1_0101	MQKYSTLQGPPGTGK	0.8687	Strong binder
DRB1_0101	AVASKILGLPTQTVD	0.8627	Strong binder
DRB1_0101	QKYSTLQGPPGTGKS	0.8584	Strong binder
DRB1_0101	TFKLSYGIATVREVL	0.8549	Strong binder
DRB1_0101	VGDYFVLTSHTVMPL	0.8535	Strong binder
DRB1_0101	GDYFVLTSHTVMPLS	0.8510	Strong binder
DRB1_0101	ISPYNSQNAVASKIL	0.8488	Strong binder
DRB1_0101	ETFKLSYGIATVREV	0.8459	Strong binder
DRB1_0101	GMQKYSTLQGPPGTG	0.8449	Strong binder
DRB1_0101	EETFKLSYGIATVRE	0.8418	Strong binder
DRB1_0101	KSHFAIGLALYYPSA	0.8413	Strong binder
DRB1_0101	FKLSYGIATVREVLS	0.8376	Strong binder
DRB1_0101	TEETFKLSYGIATVR	0.8363	Strong binder
DRB1_0101	GKSHFAIGLALYYPS	0.8354	Strong binder
DRB1_0101	SPYNSQNAVASKILG	0.8335	Strong binder
DRB1_0101	NVGDYFVLTSHTVMP	0.8331	Strong binder
DRB1_0101	DYFVLTSHTVMPLSA	0.8320	Strong binder
DRB1_0101	KYSTLQGPPGTGKSH	0.8313	Strong binder
DRB1_0101	KILGLPTQTVDSSQG	0.8259	Strong binder
DRB1_0101	LNVGDYFVLTSHTVM	0.8224	Strong binder
DRB1_0101	FISPYNSQNAVASKI	0.8224	Strong binder
DRB1_0101	TGKSHFAIGLALYYP	0.8223	Strong binder
DRB1_0101	VGMQKYSTLQGPPGT	0.8146	Strong binder
DRB1_0101	GTGKSHFAIGLALYY	0.8133	Strong binder
DRB1_0101	NAVASKILGLPTQTV	0.8111	Strong binder

Table S8. HTL epitope of SARS-CoV-2 Spike protein having strong binding affinity, predicted by NetMHC-II.

alleles	peptides	core Of log50k (aff)	Level of binding
DRB1_0101	VLSFELLHAPATVCG	0.9082	Strong binder
DRB1_0101	LSFELLHAPATVCGP	0.9033	Strong binder
DRB1_0101	VVLSFELLHAPATVC	0.9010	Strong binder
DRB1_0101	SFELLHAPATVCGPK	0.8909	Strong binder
DRB1_0101	VVVLSFELLHAPATV	0.8905	Strong binder
DRB1_0101	LQTYVTQQLIRAAEI	0.8745	Strong binder
DRB1_0101	YYVGYLQPRTFLLKY	0.8736	Strong binder
DRB1_0101	YVGYLQPRTFLLKYN	0.8685	Strong binder
DRB1_0101	QTYVTQQLIRAAEIR	0.8673	Strong binder
DRB1_0101	TGRLQSLQTYVTQQL	0.8638	Strong binder
DRB1_0101	AYYVGYLQPRTFLLK	0.8627	Strong binder
DRB1_0101	SLQTYVTQQLIRAAE	0.8532	Strong binder
DRB1_0101	IIAYTMSLGAENSVA	0.8529	Strong binder
DRB1_0101	ITGRLQSLQTYVTQQ	0.8523	Strong binder
DRB1_0101	GWTFGAGAALQIPFA	0.8445	Strong binder
DRB1_0101	IAYTMSLGAENSVAY	0.8429	Strong binder
DRB1_0101	FELLHAPATVCGPKK	0.8406	Strong binder
DRB1_0101	VGYLQPRTFLLKYNE	0.8399	Strong binder
DRB1_0101	GRLQSLQTYVTQQLI	0.8386	Strong binder
DRB1_0101	QSLQTYVTQQLIRAA	0.8351	Strong binder
DRB1_0101	LNTLVKQLSSNFGAI	0.8337	Strong binder
DRB1_0101	WTFGAGAALQIPFAM	0.8331	Strong binder
DRB1_0101	SIIAYTMSLGAENSV	0.8331	Strong binder
DRB1_0101	NTLVKQLSSNFGAIS	0.8330	Strong binder
DRB1_0101	LITGRLQSLQTYVTQ	0.8329	Strong binder
DRB1_0101	RVVVLSFELLHAPAT	0.8315	Strong binder
DRB1_0101	SSNFGAISSVLNDIL	0.8297	Strong binder
DRB1_0101	AAYYVGYLQPRTFLL	0.8264	Strong binder
DRB1_0101	GKGYHLMSFPQSAPH	0.8264	Strong binder
DRB1_0101	SGWTFGAGAALQIPF	0.8240	Strong binder
DRB1_0101	KGYHLMSFPQSAPHG	0.8223	Strong binder
DRB1_0101	TYVTQQLIRAAEIRA	0.8219	Strong binder
DRB1_0101	AQKFNGLTVLPPLLT	0.8195	Strong binder
DRB1_0101	QIPFAMQMAYRFNGI	0.8164	Strong binder
DRB1_0101	LSSNFGAISSVLNDI	0.8149	Strong binder
DRB1_0101	TRFQTLLALHRSYLT	0.8132	Strong binder
DRB1_0101	AYTMSLGAENSVAYS	0.8128	Strong binder

DRB1_0101	NFNFNGLTGTGVLTE	0.8104	Strong binder
DRB1_0101	ALNTLVKQLSSNFGA	0.8094	Strong binder
DRB1_0101	CGKGYHLMSFPQSAP	0.8087	Strong binder
DRB1_0101	QKFNGLTVLPPLLTD	0.8084	Strong binder
DRB1_0101	SNFGAISSVLNDILS	0.8082	Strong binder
DRB1_0101	LQIPFAMQMAYRFNG	0.8069	Strong binder
DRB1_0101	VNFNFNGLTGTGVLT	0.8064	Strong binder
DRB1_0101	TLVKQLSSNFGAISS	0.8047	Strong binder
DRB1_0101	TSGWTFGAGAALQIP	0.8027	Strong binder

Table S9. List of all Molecular interactions of MVC (Chain B) with TLR3 (Chain A).

Hydrogen bonds

	<----- A T O M 1 ----->						<----- A T O M 2 ----->					
	Atom. no.	Atom name	Res name	Res no.	Chain		Atom no.	Atom name	Res name	Res no.	Chain	Distance
1.	88	O	HIS	39	A	<-->	14203	N	GLY	881	B	2.82
2.	82	ND1	HIS	39	A	<-->	14185	O	THR	879	B	2.93
3.	97	O	LEU	40	A	<-->	14290	ND2	ASN	893	B	2.83
4.	105	NZ	LYS	41	A	<-->	14202	O	ARG	880	B	2.66
5.	105	NZ	LYS	41	A	<-->	14207	O	GLY	881	B	2.53
6.	105	NZ	LYS	41	A	<-->	14218	OG1	THR	883	B	2.74
7.	4250	OH	TYR	468	A	<-->	6609	OH	TYR	49	B	2.75
8.	4477	O	ARG	489	A	<-->	6648	OH	TYR	53	B	2.81
9.	4467	NE	ARG	489	A	<-->	6682	SG	CYS	57	B	3.22
10.	4738	ND2	ASN	517	A	<-->	7221	OH	TYR	116	B	2.85
11.	4950	O	HIS	539	A	<-->	6674	OH	TYR	56	B	2.74

12.	4944	ND1	HIS	539	A	<-->	7195	OH	TYR	113	B	2.83
13.	5263	O	SER	571	A	<-->	7195	OH	TYR	113	B	3.08
14.	5719	NZ	LYS	619	A	<-->	7134	O	THR	106	B	2.61
15.	5918	OE2	GLU	639	A	<-->	15038	OG1	THR	980	B	2.68
16.	5918	OE2	GLU	639	A	<-->	15043	N	SER	981	B	3.33
17.	5918	OE2	GLU	639	A	<-->	15047	OG	SER	981	B	2.87
18.	5964	O	ARG	643	A	<-->	14905	NH1	ARG	964	B	2.80
19.	5957	NH1	ARG	643	A	<-->	15109	OG	SER	988	B	2.72
20.	6100	N	VAL	658	A	<-->	14963	O	PRO	971	B	2.86
21.	6114	ND2	ASN	659	A	<-->	15016	O	PHE	977	B	2.87
22.	6114	ND2	ASN	659	A	<-->	15033	O	LEU	979	B	2.83
23.	6154	O	ASN	662	A	<-->	14475	NZ	LYS	914	B	2.56
24.	6164	O	GLU	663	A	<-->	14905	NH1	ARG	964	B	2.52
25.	6161	OE1	GLU	663	A	<-->	14475	NZ	LYS	914	B	2.64
26.	6162	OE2	GLU	663	A	<-->	14475	NZ	LYS	914	B	2.60
27.	6169	OG1	THR	664	A	<-->	14922	O	GLU	965	B	3.15

Non-bonded contacts

<----- A T O M 1 ----->   <----- A T O M 2 ----->

Atom Atom Res Res   Atom Atom Res Res

	no.	name	name	no.	Chain		no.	name	name	no.	Chain	Distance
1.	79	CA	HIS	39	A	<-->	14185	O	THR	879	B	3.50
2.	87	C	HIS	39	A	<-->	14201	C	ARG	880	B	3.81
3.	87	C	HIS	39	A	<-->	14203	N	GLY	881	B	3.60
4.	88	O	HIS	39	A	<-->	14176	O	ALA	878	B	2.97
5.	88	O	HIS	39	A	<-->	14201	C	ARG	880	B	3.44
6.	88	O	HIS	39	A	<-->	14203	N	GLY	881	B	2.82
7.	88	O	HIS	39	A	<-->	14205	CA	GLY	881	B	3.22
8.	80	CB	HIS	39	A	<-->	14176	O	ALA	878	B	3.25
9.	80	CB	HIS	39	A	<-->	14179	CA	THR	879	B	3.49
10.	80	CB	HIS	39	A	<-->	14184	C	THR	879	B	3.41
11.	80	CB	HIS	39	A	<-->	14185	O	THR	879	B	3.26
12.	81	CG	HIS	39	A	<-->	14179	CA	THR	879	B	3.58
13.	81	CG	HIS	39	A	<-->	14184	C	THR	879	B	3.81
14.	81	CG	HIS	39	A	<-->	14185	O	THR	879	B	3.48
15.	82	ND1	HIS	39	A	<-->	14179	CA	THR	879	B	3.33
16.	82	ND1	HIS	39	A	<-->	14184	C	THR	879	B	3.50
17.	82	ND1	HIS	39	A	<-->	14185	O	THR	879	B	2.93
18.	82	ND1	HIS	39	A	<-->	14180	CB	THR	879	B	3.43
19.	82	ND1	HIS	39	A	<-->	14183	CG2	THR	879	B	3.66
20.	86	CE1	HIS	39	A	<-->	6935	O	LEU	85	B	3.07
21.	86	CE1	HIS	39	A	<-->	14183	CG2	THR	879	B	3.69
22.	85	NE2	HIS	39	A	<-->	6935	O	LEU	85	B	3.80

23.	85	NE2	HIS	39	A	<-->	6942	CD2	PHE	86	B	3.50
24.	91	CA	LEU	40	A	<-->	14202	O	ARG	880	B	3.80
25.	91	CA	LEU	40	A	<-->	14203	N	GLY	881	B	3.84
26.	91	CA	LEU	40	A	<-->	14205	CA	GLY	881	B	3.57
27.	91	CA	LEU	40	A	<-->	14289	OD1	ASN	893	B	3.89
28.	96	C	LEU	40	A	<-->	14202	O	ARG	880	B	3.36
29.	96	C	LEU	40	A	<-->	14290	ND2	ASN	893	B	3.84
30.	97	O	LEU	40	A	<-->	14202	O	ARG	880	B	3.86
31.	97	O	LEU	40	A	<-->	14288	CG	ASN	893	B	3.42
32.	97	O	LEU	40	A	<-->	14289	OD1	ASN	893	B	3.24
33.	97	O	LEU	40	A	<-->	14290	ND2	ASN	893	B	2.83
34.	92	CB	LEU	40	A	<-->	14288	CG	ASN	893	B	3.86
35.	92	CB	LEU	40	A	<-->	14289	OD1	ASN	893	B	3.70
36.	92	CB	LEU	40	A	<-->	14290	ND2	ASN	893	B	3.47
37.	95	CD2	LEU	40	A	<-->	14205	CA	GLY	881	B	3.81
38.	95	CD2	LEU	40	A	<-->	14294	O	ASN	893	B	3.61
39.	95	CD2	LEU	40	A	<-->	14289	OD1	ASN	893	B	3.49
40.	98	N	LYS	41	A	<-->	14201	C	ARG	880	B	3.77
41.	98	N	LYS	41	A	<-->	14202	O	ARG	880	B	3.16
42.	100	CA	LYS	41	A	<-->	14202	O	ARG	880	B	3.65
43.	101	CB	LYS	41	A	<-->	14202	O	ARG	880	B	3.04
44.	102	CG	LYS	41	A	<-->	14202	O	ARG	880	B	3.46
45.	104	CE	LYS	41	A	<-->	14202	O	ARG	880	B	3.36

46.	104	CE	LYS	41	A	<-->	14189	CB	ARG	880	B	3.79
47.	104	CE	LYS	41	A	<-->	14207	O	GLY	881	B	3.84
48.	104	CE	LYS	41	A	<-->	14218	OG1	THR	883	B	3.42
49.	105	NZ	LYS	41	A	<-->	14201	C	ARG	880	B	3.63
50.	105	NZ	LYS	41	A	<-->	14202	O	ARG	880	B	2.66
51.	105	NZ	LYS	41	A	<-->	14206	C	GLY	881	B	3.54
52.	105	NZ	LYS	41	A	<-->	14207	O	GLY	881	B	2.53
53.	105	NZ	LYS	41	A	<-->	14218	OG1	THR	883	B	2.74
54.	117	CD2	LEU	42	A	<-->	14290	ND2	ASN	893	B	3.48
55.	283	CE1	HIS	60	A	<-->	6944	CE2	PHE	86	B	3.75
56.	282	NE2	HIS	60	A	<-->	6930	CB	LEU	85	B	3.74
57.	282	NE2	HIS	60	A	<-->	6932	CD1	LEU	85	B	3.54
58.	528	CE2	PHE	84	A	<-->	6932	CD1	LEU	85	B	3.69
59.	529	CZ	PHE	84	A	<-->	6932	CD1	LEU	85	B	3.56
60.	4226	O	ASN	466	A	<-->	6644	CE1	TYR	53	B	3.66
61.	4226	O	ASN	466	A	<-->	6647	CZ	TYR	53	B	3.42
62.	4226	O	ASN	466	A	<-->	6648	OH	TYR	53	B	3.36
63.	4239	O	LYS	467	A	<-->	6642	CG	TYR	53	B	3.78
64.	4239	O	LYS	467	A	<-->	6643	CD1	TYR	53	B	3.76
65.	4245	CD1	TYR	468	A	<-->	6625	CG1	VAL	51	B	3.60
66.	4246	CE1	TYR	468	A	<-->	6607	CE2	TYR	49	B	3.83
67.	4249	CZ	TYR	468	A	<-->	6609	OH	TYR	49	B	3.88
68.	4250	OH	TYR	468	A	<-->	6607	CE2	TYR	49	B	3.43

69.	4250	OH	TYR	468	A	<-->	6608	CZ	TYR	49	B	3.47
70.	4250	OH	TYR	468	A	<-->	6609	OH	TYR	49	B	2.75
71.	4476	C	ARG	489	A	<-->	6644	CE1	TYR	53	B	3.58
72.	4476	C	ARG	489	A	<-->	6648	OH	TYR	53	B	3.85
73.	4477	O	ARG	489	A	<-->	6644	CE1	TYR	53	B	3.13
74.	4477	O	ARG	489	A	<-->	6647	CZ	TYR	53	B	3.39
75.	4477	O	ARG	489	A	<-->	6648	OH	TYR	53	B	2.81
76.	4465	CG	ARG	489	A	<-->	6644	CE1	TYR	53	B	3.80
77.	4465	CG	ARG	489	A	<-->	6648	OH	TYR	53	B	3.42
78.	4467	NE	ARG	489	A	<-->	6682	SG	CYS	57	B	3.22
79.	4469	CZ	ARG	489	A	<-->	6682	SG	CYS	57	B	3.66
80.	4469	CZ	ARG	489	A	<-->	6700	CG	ASP	59	B	3.60
81.	4469	CZ	ARG	489	A	<-->	6701	OD1	ASP	59	B	3.16
82.	4469	CZ	ARG	489	A	<-->	6702	OD2	ASP	59	B	3.19
83.	4470	NH1	ARG	489	A	<-->	6700	CG	ASP	59	B	3.15
84.	4470	NH1	ARG	489	A	<-->	6701	OD1	ASP	59	B	2.84
85.	4470	NH1	ARG	489	A	<-->	6702	OD2	ASP	59	B	2.78
86.	4473	NH2	ARG	489	A	<-->	6680	CA	CYS	57	B	3.57
87.	4473	NH2	ARG	489	A	<-->	6681	CB	CYS	57	B	3.38
88.	4473	NH2	ARG	489	A	<-->	6682	SG	CYS	57	B	3.52
89.	4473	NH2	ARG	489	A	<-->	6700	CG	ASP	59	B	3.13
90.	4473	NH2	ARG	489	A	<-->	6701	OD1	ASP	59	B	2.75
91.	4473	NH2	ARG	489	A	<-->	6702	OD2	ASP	59	B	2.79

92.	4473	NH2	ARG	489	A	<-->	6706	CD	PRO	60	B	3.83
93.	4486	N	ALA	491	A	<-->	6643	CD1	TYR	53	B	3.73
94.	4486	N	ALA	491	A	<-->	6644	CE1	TYR	53	B	3.82
95.	4489	CB	ALA	491	A	<-->	6643	CD1	TYR	53	B	3.65
96.	4712	CA	ASN	515	A	<-->	6674	OH	TYR	56	B	3.01
97.	4719	C	ASN	515	A	<-->	6672	CE2	TYR	56	B	3.59
98.	4719	C	ASN	515	A	<-->	6673	CZ	TYR	56	B	3.84
99.	4719	C	ASN	515	A	<-->	6674	OH	TYR	56	B	3.33
100.	4720	O	ASN	515	A	<-->	6672	CE2	TYR	56	B	3.08
101.	4720	O	ASN	515	A	<-->	6673	CZ	TYR	56	B	3.78
102.	4720	O	ASN	515	A	<-->	6674	OH	TYR	56	B	3.65
103.	4713	CB	ASN	515	A	<-->	6674	OH	TYR	56	B	3.56
104.	4736	CG	ASN	517	A	<-->	6669	CD1	TYR	56	B	3.49
105.	4736	CG	ASN	517	A	<-->	6670	CE1	TYR	56	B	3.50
106.	4737	OD1	ASN	517	A	<-->	6669	CD1	TYR	56	B	3.45
107.	4737	OD1	ASN	517	A	<-->	6670	CE1	TYR	56	B	3.07
108.	4737	OD1	ASN	517	A	<-->	6673	CZ	TYR	56	B	3.87
109.	4738	ND2	ASN	517	A	<-->	6669	CD1	TYR	56	B	3.37
110.	4738	ND2	ASN	517	A	<-->	6670	CE1	TYR	56	B	3.88
111.	4738	ND2	ASN	517	A	<-->	7220	CZ	TYR	116	B	3.78
112.	4738	ND2	ASN	517	A	<-->	7221	OH	TYR	116	B	2.85
113.	4941	CA	HIS	539	A	<-->	7195	OH	TYR	113	B	3.42
114.	4949	C	HIS	539	A	<-->	6674	OH	TYR	56	B	3.66

115.	4950	O	HIS	539	A	<-->	6673	CZ	TYR	56	B	3.85
116.	4950	O	HIS	539	A	<-->	6674	OH	TYR	56	B	2.74
117.	4942	CB	HIS	539	A	<-->	6674	OH	TYR	56	B	3.17
118.	4942	CB	HIS	539	A	<-->	7195	OH	TYR	113	B	3.64
119.	4943	CG	HIS	539	A	<-->	7195	OH	TYR	113	B	3.59
120.	4944	ND1	HIS	539	A	<-->	7191	CE1	TYR	113	B	3.58
121.	4944	ND1	HIS	539	A	<-->	7194	CZ	TYR	113	B	3.64
122.	4944	ND1	HIS	539	A	<-->	7195	OH	TYR	113	B	2.83
123.	5263	O	SER	571	A	<-->	7195	OH	TYR	113	B	3.08
124.	5259	CB	SER	571	A	<-->	7195	OH	TYR	113	B	3.38
125.	5485	CG	LEU	595	A	<-->	7157	OD2	ASP	109	B	3.32
126.	5487	CD2	LEU	595	A	<-->	7154	CB	ASP	109	B	3.60
127.	5487	CD2	LEU	595	A	<-->	7155	CG	ASP	109	B	3.86
128.	5487	CD2	LEU	595	A	<-->	7157	OD2	ASP	109	B	3.69
129.	5684	CG	ASN	616	A	<-->	15086	CE	MET	985	B	3.72
130.	5686	ND2	ASN	616	A	<-->	15086	CE	MET	985	B	3.63
131.	5705	CD	GLN	618	A	<-->	15085	SD	MET	985	B	3.51
132.	5705	CD	GLN	618	A	<-->	15086	CE	MET	985	B	3.72
133.	5706	OE1	GLN	618	A	<-->	15085	SD	MET	985	B	3.76
134.	5706	OE1	GLN	618	A	<-->	15086	CE	MET	985	B	3.68
135.	5707	NE2	GLN	618	A	<-->	15085	SD	MET	985	B	3.58
136.	5707	NE2	GLN	618	A	<-->	15086	CE	MET	985	B	3.50
137.	5717	CD	LYS	619	A	<-->	7157	OD2	ASP	109	B	3.15

138.	5718	CE	LYS	619	A	<-->	7134	O	THR	106	B	3.36
139.	5718	CE	LYS	619	A	<-->	7156	OD1	ASP	109	B	3.70
140.	5718	CE	LYS	619	A	<-->	7157	OD2	ASP	109	B	3.55
141.	5719	NZ	LYS	619	A	<-->	7125	O	LYS	105	B	3.56
142.	5719	NZ	LYS	619	A	<-->	7128	CA	THR	106	B	3.81
143.	5719	NZ	LYS	619	A	<-->	7133	C	THR	106	B	3.50
144.	5719	NZ	LYS	619	A	<-->	7134	O	THR	106	B	2.61
145.	5719	NZ	LYS	619	A	<-->	7155	CG	ASP	109	B	2.94
146.	5719	NZ	LYS	619	A	<-->	7156	OD1	ASP	109	B	2.55
147.	5719	NZ	LYS	619	A	<-->	7157	OD2	ASP	109	B	2.88
148.	5861	CE2	PHE	634	A	<-->	14949	CG	PRO	969	B	3.58
149.	5915	CG	GLU	639	A	<-->	15038	OG1	THR	980	B	3.70
150.	5916	CD	GLU	639	A	<-->	15038	OG1	THR	980	B	3.57
151.	5916	CD	GLU	639	A	<-->	15047	OG	SER	981	B	3.81
152.	5918	OE2	GLU	639	A	<-->	15037	CB	THR	980	B	3.52
153.	5918	OE2	GLU	639	A	<-->	15038	OG1	THR	980	B	2.68
154.	5918	OE2	GLU	639	A	<-->	15040	CG2	THR	980	B	3.34
155.	5918	OE2	GLU	639	A	<-->	15043	N	SER	981	B	3.33
156.	5918	OE2	GLU	639	A	<-->	15045	CA	SER	981	B	3.87
157.	5918	OE2	GLU	639	A	<-->	15046	CB	SER	981	B	3.32
158.	5918	OE2	GLU	639	A	<-->	15047	OG	SER	981	B	2.87
159.	5927	CD2	LEU	640	A	<-->	14949	CG	PRO	969	B	3.79
160.	5927	CD2	LEU	640	A	<-->	14946	CD	PRO	969	B	3.71

161.	5936	OD2	ASP	641	A	<-->	15084	CG	MET	985	B	3.58
162.	5936	OD2	ASP	641	A	<-->	15085	SD	MET	985	B	3.34
163.	5963	C	ARG	643	A	<-->	14905	NH1	ARG	964	B	3.48
164.	5964	O	ARG	643	A	<-->	14901	CD	ARG	964	B	3.75
165.	5964	O	ARG	643	A	<-->	14905	NH1	ARG	964	B	2.80
166.	5953	CD	ARG	643	A	<-->	15109	OG	SER	988	B	3.17
167.	5954	NE	ARG	643	A	<-->	15109	OG	SER	988	B	3.88
168.	5956	CZ	ARG	643	A	<-->	15096	N	LEU	987	B	3.60
169.	5956	CZ	ARG	643	A	<-->	15098	CA	LEU	987	B	3.80
170.	5956	CZ	ARG	643	A	<-->	15109	OG	SER	988	B	3.72
171.	5957	NH1	ARG	643	A	<-->	15093	CG	PRO	986	B	3.84
172.	5957	NH1	ARG	643	A	<-->	15096	N	LEU	987	B	3.27
173.	5957	NH1	ARG	643	A	<-->	15098	CA	LEU	987	B	3.51
174.	5957	NH1	ARG	643	A	<-->	15103	C	LEU	987	B	3.69
175.	5957	NH1	ARG	643	A	<-->	15105	N	SER	988	B	3.30
176.	5957	NH1	ARG	643	A	<-->	15108	CB	SER	988	B	3.89
177.	5957	NH1	ARG	643	A	<-->	15109	OG	SER	988	B	2.72
178.	5960	NH2	ARG	643	A	<-->	15084	CG	MET	985	B	3.81
179.	5960	NH2	ARG	643	A	<-->	15085	SD	MET	985	B	3.24
180.	5960	NH2	ARG	643	A	<-->	15093	CG	PRO	986	B	3.68
181.	5960	NH2	ARG	643	A	<-->	15096	N	LEU	987	B	3.79
182.	5970	CD1	PHE	644	A	<-->	9029	CB	THR	313	B	3.86
183.	5970	CD1	PHE	644	A	<-->	9032	CG2	THR	313	B	3.53

184.	5972	CE1	PHE	644	A	<-->	9029	CB	THR	313	B	3.62
185.	5972	CE1	PHE	644	A	<-->	9030	OG1	THR	313	B	3.67
186.	5972	CE1	PHE	644	A	<-->	9032	CG2	THR	313	B	3.85
187.	5972	CE1	PHE	644	A	<-->	15109	OG	SER	988	B	3.46
188.	5973	CE2	PHE	644	A	<-->	15109	OG	SER	988	B	3.84
189.	5974	CZ	PHE	644	A	<-->	15109	OG	SER	988	B	3.03
190.	5977	N	ASN	645	A	<-->	14905	NH1	ARG	964	B	3.40
191.	5986	C	ASN	645	A	<-->	14904	CZ	ARG	964	B	3.86
192.	5987	O	ASN	645	A	<-->	14901	CD	ARG	964	B	3.59
193.	5987	O	ASN	645	A	<-->	14902	NE	ARG	964	B	2.88
194.	5987	O	ASN	645	A	<-->	14904	CZ	ARG	964	B	2.70
195.	5987	O	ASN	645	A	<-->	14905	NH1	ARG	964	B	3.17
196.	5987	O	ASN	645	A	<-->	14908	NH2	ARG	964	B	2.97
197.	5990	CA	PRO	646	A	<-->	14892	CG1	VAL	963	B	3.69
198.	5993	C	PRO	646	A	<-->	14892	CG1	VAL	963	B	3.55
199.	5995	N	PHE	647	A	<-->	14892	CG1	VAL	963	B	3.23
200.	6006	O	PHE	647	A	<-->	14892	CG1	VAL	963	B	3.19
201.	5998	CB	PHE	647	A	<-->	14908	NH2	ARG	964	B	3.37
202.	5999	CG	PHE	647	A	<-->	14908	NH2	ARG	964	B	3.24
203.	6001	CD2	PHE	647	A	<-->	14908	NH2	ARG	964	B	3.29
204.	6027	OG1	THR	650	A	<-->	14928	CG2	VAL	966	B	3.81
205.	6051	CA	SER	653	A	<-->	14947	CA	PRO	969	B	3.68
206.	6051	CA	SER	653	A	<-->	14950	C	PRO	969	B	3.70

207.	6051	CA	SER	653	A	<-->	14951	O	PRO	969	B	3.85
208.	6052	CB	SER	653	A	<-->	14943	C	GLY	968	B	3.41
209.	6052	CB	SER	653	A	<-->	14944	O	GLY	968	B	2.99
210.	6052	CB	SER	653	A	<-->	14945	N	PRO	969	B	3.61
211.	6052	CB	SER	653	A	<-->	14947	CA	PRO	969	B	3.26
212.	6052	CB	SER	653	A	<-->	14950	C	PRO	969	B	3.15
213.	6052	CB	SER	653	A	<-->	14951	O	PRO	969	B	3.71
214.	6052	CB	SER	653	A	<-->	14952	N	GLY	970	B	3.18
215.	6052	CB	SER	653	A	<-->	14954	CA	GLY	970	B	3.74
216.	6053	OG	SER	653	A	<-->	14944	O	GLY	968	B	3.40
217.	6057	N	ILE	654	A	<-->	14951	O	PRO	969	B	3.66
218.	6065	O	ILE	654	A	<-->	14961	CG	PRO	971	B	3.72
219.	6065	O	ILE	654	A	<-->	14958	CD	PRO	971	B	3.02
220.	6072	N	TRP	656	A	<-->	14958	CD	PRO	971	B	3.50
221.	6086	C	TRP	656	A	<-->	14958	CD	PRO	971	B	3.68
222.	6087	O	TRP	656	A	<-->	14951	O	PRO	969	B	3.74
223.	6087	O	TRP	656	A	<-->	14952	N	GLY	970	B	3.81
224.	6087	O	TRP	656	A	<-->	14954	CA	GLY	970	B	3.00
225.	6087	O	TRP	656	A	<-->	14955	C	GLY	970	B	3.26
226.	6087	O	TRP	656	A	<-->	14957	N	PRO	971	B	3.21
227.	6087	O	TRP	656	A	<-->	14958	CD	PRO	971	B	3.07
228.	6079	CE3	TRP	656	A	<-->	14951	O	PRO	969	B	3.82
229.	6084	CZ3	TRP	656	A	<-->	14951	O	PRO	969	B	3.60

230.	6084	CZ3	TRP	656	A	<-->	14948	CB	PRO	969	B	3.84
231.	6085	CH2	TRP	656	A	<-->	14951	O	PRO	969	B	3.75
232.	6085	CH2	TRP	656	A	<-->	14948	CB	PRO	969	B	3.56
233.	6090	CA	PHE	657	A	<-->	14963	O	PRO	971	B	3.60
234.	6098	C	PHE	657	A	<-->	14963	O	PRO	971	B	3.71
235.	6093	CD1	PHE	657	A	<-->	14963	O	PRO	971	B	3.32
236.	6094	CD2	PHE	657	A	<-->	15038	OG1	THR	980	B	3.25
237.	6095	CE1	PHE	657	A	<-->	14963	O	PRO	971	B	3.82
238.	6096	CE2	PHE	657	A	<-->	15033	O	LEU	979	B	3.49
239.	6096	CE2	PHE	657	A	<-->	15038	OG1	THR	980	B	3.70
240.	6097	CZ	PHE	657	A	<-->	15011	CD2	PHE	977	B	3.38
241.	6097	CZ	PHE	657	A	<-->	15013	CE2	PHE	977	B	3.85
242.	6100	N	VAL	658	A	<-->	14955	C	GLY	970	B	3.87
243.	6100	N	VAL	658	A	<-->	14956	O	GLY	970	B	3.67
244.	6100	N	VAL	658	A	<-->	14962	C	PRO	971	B	3.88
245.	6100	N	VAL	658	A	<-->	14963	O	PRO	971	B	2.86
246.	6102	CA	VAL	658	A	<-->	14956	O	GLY	970	B	3.72
247.	6102	CA	VAL	658	A	<-->	14963	O	PRO	971	B	3.72
248.	6106	C	VAL	658	A	<-->	14963	O	PRO	971	B	3.60
249.	6107	O	VAL	658	A	<-->	14956	O	GLY	970	B	3.40
250.	6107	O	VAL	658	A	<-->	14962	C	PRO	971	B	3.74
251.	6107	O	VAL	658	A	<-->	14963	O	PRO	971	B	3.30
252.	6107	O	VAL	658	A	<-->	14964	N	GLY	972	B	3.77

253.	6107	O	VAL	658	A	<-->	14966	CA	GLY	972	B	3.01
254.	6107	O	VAL	658	A	<-->	14967	C	GLY	972	B	3.29
255.	6107	O	VAL	658	A	<-->	14969	N	VAL	973	B	3.50
256.	6103	CB	VAL	658	A	<-->	14944	O	GLY	968	B	3.90
257.	6103	CB	VAL	658	A	<-->	14950	C	PRO	969	B	3.78
258.	6103	CB	VAL	658	A	<-->	14952	N	GLY	970	B	3.11
259.	6103	CB	VAL	658	A	<-->	14954	CA	GLY	970	B	3.80
260.	6103	CB	VAL	658	A	<-->	14955	C	GLY	970	B	3.71
261.	6103	CB	VAL	658	A	<-->	14956	O	GLY	970	B	3.17
262.	6104	CG1	VAL	658	A	<-->	14943	C	GLY	968	B	3.49
263.	6104	CG1	VAL	658	A	<-->	14944	O	GLY	968	B	3.27
264.	6104	CG1	VAL	658	A	<-->	14945	N	PRO	969	B	3.88
265.	6104	CG1	VAL	658	A	<-->	14952	N	GLY	970	B	3.65
266.	6104	CG1	VAL	658	A	<-->	14956	O	GLY	970	B	3.59
267.	6105	CG2	VAL	658	A	<-->	14945	N	PRO	969	B	3.79
268.	6105	CG2	VAL	658	A	<-->	14947	CA	PRO	969	B	3.63
269.	6105	CG2	VAL	658	A	<-->	14950	C	PRO	969	B	3.40
270.	6105	CG2	VAL	658	A	<-->	14948	CB	PRO	969	B	3.72
271.	6105	CG2	VAL	658	A	<-->	14952	N	GLY	970	B	3.31
272.	6118	O	ASN	659	A	<-->	15019	CA	VAL	978	B	3.54
273.	6118	O	ASN	659	A	<-->	15021	CG1	VAL	978	B	3.72
274.	6111	CB	ASN	659	A	<-->	15016	O	PHE	977	B	3.90
275.	6111	CB	ASN	659	A	<-->	15023	C	VAL	978	B	3.82

276.	6111	CB	ASN	659	A	<-->	15024	O	VAL	978	B	3.34
277.	6112	CG	ASN	659	A	<-->	15016	O	PHE	977	B	3.84
278.	6112	CG	ASN	659	A	<-->	15024	O	VAL	978	B	3.66
279.	6112	CG	ASN	659	A	<-->	15037	CB	THR	980	B	3.51
280.	6113	OD1	ASN	659	A	<-->	15037	CB	THR	980	B	3.08
281.	6113	OD1	ASN	659	A	<-->	15038	OG1	THR	980	B	3.28
282.	6113	OD1	ASN	659	A	<-->	15040	CG2	THR	980	B	3.82
283.	6114	ND2	ASN	659	A	<-->	15016	O	PHE	977	B	2.87
284.	6114	ND2	ASN	659	A	<-->	15023	C	VAL	978	B	3.46
285.	6114	ND2	ASN	659	A	<-->	15024	O	VAL	978	B	3.41
286.	6114	ND2	ASN	659	A	<-->	15025	N	LEU	979	B	3.73
287.	6114	ND2	ASN	659	A	<-->	15032	C	LEU	979	B	3.18
288.	6114	ND2	ASN	659	A	<-->	15033	O	LEU	979	B	2.83
289.	6114	ND2	ASN	659	A	<-->	15034	N	THR	980	B	3.60
290.	6114	ND2	ASN	659	A	<-->	15036	CA	THR	980	B	3.78
291.	6114	ND2	ASN	659	A	<-->	15037	CB	THR	980	B	3.56
292.	6133	C	TRP	660	A	<-->	14968	O	GLY	972	B	3.52
293.	6134	O	TRP	660	A	<-->	14967	C	GLY	972	B	3.39
294.	6134	O	TRP	660	A	<-->	14968	O	GLY	972	B	3.32
295.	6134	O	TRP	660	A	<-->	14969	N	VAL	973	B	3.30
296.	6134	O	TRP	660	A	<-->	14971	CA	VAL	973	B	3.37
297.	6134	O	TRP	660	A	<-->	14972	CB	VAL	973	B	3.81
298.	6134	O	TRP	660	A	<-->	14973	CG1	VAL	973	B	3.07

299.	6131	CZ3	TRP	660	A	<-->	14905	NH1	ARG	964	B	3.83
300.	6132	CH2	TRP	660	A	<-->	14942	CA	GLY	968	B	3.90
301.	6135	N	ILE	661	A	<-->	14968	O	GLY	972	B	3.64
302.	6137	CA	ILE	661	A	<-->	14968	O	GLY	972	B	3.87
303.	6138	CB	ILE	661	A	<-->	14968	O	GLY	972	B	3.20
304.	6138	CB	ILE	661	A	<-->	14977	N	GLY	974	B	3.84
305.	6139	CG2	ILE	661	A	<-->	14968	O	GLY	972	B	3.47
306.	6139	CG2	ILE	661	A	<-->	14996	CD1	TYR	976	B	3.43
307.	6141	CD1	ILE	661	A	<-->	14977	N	GLY	974	B	3.63
308.	6141	CD1	ILE	661	A	<-->	14979	CA	GLY	974	B	3.85
309.	6141	CD1	ILE	661	A	<-->	14980	C	GLY	974	B	3.69
310.	6141	CD1	ILE	661	A	<-->	14981	O	GLY	974	B	3.13
311.	6141	CD1	ILE	661	A	<-->	14997	CE1	TYR	976	B	3.57
312.	6153	C	ASN	662	A	<-->	14475	NZ	LYS	914	B	3.39
313.	6153	C	ASN	662	A	<-->	14912	O	ARG	964	B	3.81
314.	6154	O	ASN	662	A	<-->	14474	CE	LYS	914	B	3.63
315.	6154	O	ASN	662	A	<-->	14475	NZ	LYS	914	B	2.56
316.	6154	O	ASN	662	A	<-->	14912	O	ARG	964	B	2.76
317.	6163	C	GLU	663	A	<-->	14905	NH1	ARG	964	B	3.70
318.	6164	O	GLU	663	A	<-->	14901	CD	ARG	964	B	3.13
319.	6164	O	GLU	663	A	<-->	14902	NE	ARG	964	B	3.37
320.	6164	O	GLU	663	A	<-->	14904	CZ	ARG	964	B	3.15
321.	6164	O	GLU	663	A	<-->	14905	NH1	ARG	964	B	2.52

322.	6159	CG	GLU	663	A	<-->	15109	OG	SER	988	B	3.58
323.	6160	CD	GLU	663	A	<-->	14475	NZ	LYS	914	B	2.93
324.	6161	OE1	GLU	663	A	<-->	14475	NZ	LYS	914	B	2.64
325.	6162	OE2	GLU	663	A	<-->	14473	CD	LYS	914	B	3.84
326.	6162	OE2	GLU	663	A	<-->	14474	CE	LYS	914	B	3.74
327.	6162	OE2	GLU	663	A	<-->	14475	NZ	LYS	914	B	2.60
328.	6167	CA	THR	664	A	<-->	14930	O	VAL	966	B	3.19
329.	6172	C	THR	664	A	<-->	14898	CA	ARG	964	B	3.43
330.	6172	C	THR	664	A	<-->	14911	C	ARG	964	B	3.02
331.	6172	C	THR	664	A	<-->	14912	O	ARG	964	B	3.05
332.	6172	C	THR	664	A	<-->	14902	NE	ARG	964	B	3.75
333.	6172	C	THR	664	A	<-->	14904	CZ	ARG	964	B	3.62
334.	6172	C	THR	664	A	<-->	14908	NH2	ARG	964	B	3.80
335.	6172	C	THR	664	A	<-->	14913	N	GLU	965	B	3.41
336.	6172	C	THR	664	A	<-->	14915	CA	GLU	965	B	3.62
337.	6172	C	THR	664	A	<-->	14921	C	GLU	965	B	3.33
338.	6172	C	THR	664	A	<-->	14922	O	GLU	965	B	3.46
339.	6172	C	THR	664	A	<-->	14923	N	VAL	966	B	3.67
340.	6172	C	THR	664	A	<-->	14929	C	VAL	966	B	3.82
341.	6172	C	THR	664	A	<-->	14930	O	VAL	966	B	2.95
342.	6173	O	THR	664	A	<-->	14895	O	VAL	963	B	3.10
343.	6173	O	THR	664	A	<-->	14898	CA	ARG	964	B	3.25
344.	6173	O	THR	664	A	<-->	14911	C	ARG	964	B	3.29

345.	6173	O	THR	664	A	<-->	14912	O	ARG	964	B	3.71
346.	6173	O	THR	664	A	<-->	14902	NE	ARG	964	B	3.05
347.	6173	O	THR	664	A	<-->	14904	CZ	ARG	964	B	2.78
348.	6173	O	THR	664	A	<-->	14905	NH1	ARG	964	B	3.44
349.	6173	O	THR	664	A	<-->	14908	NH2	ARG	964	B	2.67
350.	6173	O	THR	664	A	<-->	14913	N	GLU	965	B	3.56
351.	6173	O	THR	664	A	<-->	14921	C	GLU	965	B	3.73
352.	6173	O	THR	664	A	<-->	14923	N	VAL	966	B	3.62
353.	6173	O	THR	664	A	<-->	14929	C	VAL	966	B	3.75
354.	6173	O	THR	664	A	<-->	14930	O	VAL	966	B	2.82
355.	6173	O	THR	664	A	<-->	14927	CG1	VAL	966	B	3.62
356.	6168	CB	THR	664	A	<-->	14921	C	GLU	965	B	3.88
357.	6168	CB	THR	664	A	<-->	14922	O	GLU	965	B	3.16
358.	6168	CB	THR	664	A	<-->	14929	C	VAL	966	B	3.67
359.	6168	CB	THR	664	A	<-->	14930	O	VAL	966	B	2.99
360.	6168	CB	THR	664	A	<-->	14933	CA	LEU	967	B	3.84
361.	6169	OG1	THR	664	A	<-->	14922	O	GLU	965	B	3.15
362.	6171	CG2	THR	664	A	<-->	14930	O	VAL	966	B	3.62
363.	6171	CG2	THR	664	A	<-->	14933	CA	LEU	967	B	3.56
364.	6171	CG2	THR	664	A	<-->	14938	C	LEU	967	B	3.84
365.	6171	CG2	THR	664	A	<-->	14940	N	GLY	968	B	3.53
366.	6171	CG2	THR	664	A	<-->	14973	CG1	VAL	973	B	3.75

Salt bridges

	<----- A T O M 1 ----->						<----- A T O M 2 ----->					
	Atom no.	Atom name	Res name	Res no.	Chain		Atom no.	Atom name	Res name	Res no.	Chain	Distance
1.	4473	NH2	ARG	489	A	<-->	6702	OD2	ASP	59	B	2.75
2.	5719	NZ	LYS	619	A	<-->	7157	OD2	ASP	109	B	2.55
3.	6161	OE1	GLU	663	A	<-->	14475	NZ	LYS	914	B	2.60

Number of salt bridges: 3

Number of hydrogen bonds: 27

Number of non-bonded contacts: 366

Table S10. List of all Molecular interactions of MVC (Chain B) with TLR4 (Chain A).

Hydrogen bonds

	<----- A T O M 1 ----->						<----- A T O M 2 ----->					
	Atom no.	Atom name	Res name	Res no.	Chain		Atom no.	Atom name	Res name	Res no.	Chain	Distance
1.	42	O	GLU	31	A	<-->	13704	NE	ARG	863	B	3.34
2.	42	O	GLU	31	A	<-->	13710	NH2	ARG	863	B	2.67
3.	39	OE1	GLU	31	A	<-->	13710	NH2	ARG	863	B	2.69
4.	50	O	VAL	32	A	<-->	13704	NE	ARG	863	B	3.26
5.	143	OE1	GLU	42	A	<-->	13021	OG	SER	789	B	2.94
6.	144	OE2	GLU	42	A	<-->	13017	N	SER	789	B	3.21
7.	1992	NH1	ARG	234	A	<-->	8467	SG	CYS	289	B	3.03
8.	2294	OD1	ASN	265	A	<-->	8488	N	ALA	292	B	3.01
9.	3022	ND2	ASN	339	A	<-->	6563	OE1	GLN	83	B	2.91
10.	3436	NE	ARG	382	A	<-->	13836	O	THR	879	B	2.97
11.	3442	NH2	ARG	382	A	<-->	13836	O	THR	879	B	3.14
12.	4354	NZ	LYS	477	A	<-->	13940	OD1	ASN	893	B	2.66
13.	5624	OE2	GLU	605	A	<-->	14069	NZ	LYS	906	B	2.71
14.	5624	OE2	GLU	605	A	<-->	14827	OH	TYR	997	B	2.84

Non-bonded contacts

	<----- A T O M 1 ----->						<----- A T O M 2 ----->					
	Atom no.	Atom name	Res name	Res no.	Chain		Atom no.	Atom name	Res name	Res no.	Chain	Distance
1.	9	C	GLU	27	A	<-->	14987	CD1	ILE	1015	B	3.72
2.	10	O	GLU	27	A	<-->	14987	CD1	ILE	1015	B	3.64
3.	11	N	PRO	28	A	<-->	14987	CD1	ILE	1015	B	3.79
4.	13	CA	PRO	28	A	<-->	13559	CB	PRO	848	B	3.81
5.	14	CB	PRO	28	A	<-->	13559	CB	PRO	848	B	3.68
6.	14	CB	PRO	28	A	<-->	14986	CG1	ILE	1015	B	3.90
7.	29	CG1	VAL	30	A	<-->	13603	O	HSD	852	B	3.67
8.	29	CG1	VAL	30	A	<-->	13642	CG	LEU	856	B	3.68
9.	29	CG1	VAL	30	A	<-->	13643	CD1	LEU	856	B	3.84
10.	29	CG1	VAL	30	A	<-->	13644	CD2	LEU	856	B	3.71
11.	30	CG2	VAL	30	A	<-->	13590	C	PHE	851	B	3.75
12.	30	CG2	VAL	30	A	<-->	13591	O	PHE	851	B	3.18
13.	30	CG2	VAL	30	A	<-->	13595	CB	HSD	852	B	3.79
14.	41	C	GLU	31	A	<-->	13644	CD2	LEU	856	B	3.67
15.	41	C	GLU	31	A	<-->	13710	NH2	ARG	863	B	3.66
16.	42	O	GLU	31	A	<-->	13643	CD1	LEU	856	B	3.67
17.	42	O	GLU	31	A	<-->	13644	CD2	LEU	856	B	3.00

18.	42	O	GLU	31	A	<-->	13704	NE	ARG	863	B	3.34
19.	42	O	GLU	31	A	<-->	13706	CZ	ARG	863	B	3.44
20.	42	O	GLU	31	A	<-->	13710	NH2	ARG	863	B	2.67
21.	36	CB	GLU	31	A	<-->	13710	NH2	ARG	863	B	3.54
22.	37	CG	GLU	31	A	<-->	13710	NH2	ARG	863	B	3.34
23.	38	CD	GLU	31	A	<-->	13710	NH2	ARG	863	B	3.39
24.	39	OE1	GLU	31	A	<-->	13706	CZ	ARG	863	B	3.73
25.	39	OE1	GLU	31	A	<-->	13710	NH2	ARG	863	B	2.69
26.	45	CA	VAL	32	A	<-->	13644	CD2	LEU	856	B	3.72
27.	50	O	VAL	32	A	<-->	13674	CD1	ILE	859	B	3.69
28.	50	O	VAL	32	A	<-->	13702	CG	ARG	863	B	3.41
29.	50	O	VAL	32	A	<-->	13703	CD	ARG	863	B	3.75
30.	50	O	VAL	32	A	<-->	13704	NE	ARG	863	B	3.26
31.	48	CG2	VAL	32	A	<-->	13635	CD2	LEU	855	B	3.82
32.	63	CG	PRO	34	A	<-->	13706	CZ	ARG	863	B	3.58
33.	63	CG	PRO	34	A	<-->	13707	NH1	ARG	863	B	3.54
34.	63	CG	PRO	34	A	<-->	13710	NH2	ARG	863	B	3.62
35.	60	CD	PRO	34	A	<-->	13704	NE	ARG	863	B	3.79
36.	60	CD	PRO	34	A	<-->	13706	CZ	ARG	863	B	3.59
37.	60	CD	PRO	34	A	<-->	13710	NH2	ARG	863	B	3.72
38.	114	CD	GLN	39	A	<-->	13062	CE2	PHE	792	B	3.88
39.	116	NE2	GLN	39	A	<-->	13062	CE2	PHE	792	B	3.70
40.	136	O	MET	41	A	<-->	13586	CD2	PHE	851	B	3.34

41.	136	O	MET	41	A	<-->	13588	CE2	PHE	851	B	3.03
42.	134	CE	MET	41	A	<-->	13021	OG	SER	789	B	3.47
43.	134	CE	MET	41	A	<-->	13058	CG	PHE	792	B	3.61
44.	134	CE	MET	41	A	<-->	13059	CD1	PHE	792	B	3.90
45.	134	CE	MET	41	A	<-->	13060	CD2	PHE	792	B	3.86
46.	134	CE	MET	41	A	<-->	13600	NE2	HSD	852	B	3.35
47.	146	O	GLU	42	A	<-->	13588	CE2	PHE	851	B	3.26
48.	146	O	GLU	42	A	<-->	13589	CZ	PHE	851	B	3.10
49.	140	CB	GLU	42	A	<-->	13588	CE2	PHE	851	B	3.77
50.	142	CD	GLU	42	A	<-->	13017	N	SER	789	B	3.85
51.	142	CD	GLU	42	A	<-->	13019	CA	SER	789	B	3.86
52.	142	CD	GLU	42	A	<-->	13020	CB	SER	789	B	3.59
53.	142	CD	GLU	42	A	<-->	13021	OG	SER	789	B	3.48
54.	143	OE1	GLU	42	A	<-->	13017	N	SER	789	B	3.70
55.	143	OE1	GLU	42	A	<-->	13019	CA	SER	789	B	3.35
56.	143	OE1	GLU	42	A	<-->	13020	CB	SER	789	B	3.31
57.	143	OE1	GLU	42	A	<-->	13021	OG	SER	789	B	2.94
58.	144	OE2	GLU	42	A	<-->	12994	O	PRO	785	B	3.40
59.	144	OE2	GLU	42	A	<-->	13011	CB	LEU	788	B	3.44
60.	144	OE2	GLU	42	A	<-->	13017	N	SER	789	B	3.21
61.	144	OE2	GLU	42	A	<-->	13019	CA	SER	789	B	3.66
62.	144	OE2	GLU	42	A	<-->	13020	CB	SER	789	B	3.51
63.	144	OE2	GLU	42	A	<-->	13021	OG	SER	789	B	3.88

64.	153	CD2	LEU	43	A	<-->	13584	CG	PHE	851	B	3.85
65.	153	CD2	LEU	43	A	<-->	13586	CD2	PHE	851	B	3.89
66.	160	CG	ASN	44	A	<-->	13526	CG1	VAL	843	B	3.63
67.	161	OD1	ASN	44	A	<-->	13526	CG1	VAL	843	B	3.66
68.	162	ND2	ASN	44	A	<-->	13526	CG1	VAL	843	B	3.84
69.	162	ND2	ASN	44	A	<-->	13534	CG	LEU	844	B	3.35
70.	162	ND2	ASN	44	A	<-->	13536	CD2	LEU	844	B	3.35
71.	187	CE2	TYR	46	A	<-->	15429	C	LYS	1057	B	3.71
72.	1992	NH1	ARG	234	A	<-->	8467	SG	CYS	289	B	3.03
73.	2265	CD1	PHE	263	A	<-->	8461	C	VAL	288	B	3.80
74.	2265	CD1	PHE	263	A	<-->	8462	O	VAL	288	B	3.46
75.	2265	CD1	PHE	263	A	<-->	8465	CA	CYS	289	B	3.76
76.	2267	CE1	PHE	263	A	<-->	8461	C	VAL	288	B	3.88
77.	2267	CE1	PHE	263	A	<-->	8460	CG2	VAL	288	B	3.86
78.	2267	CE1	PHE	263	A	<-->	8463	N	CYS	289	B	3.73
79.	2267	CE1	PHE	263	A	<-->	8465	CA	CYS	289	B	3.71
80.	2293	CG	ASN	265	A	<-->	8485	CB	ALA	291	B	3.84
81.	2294	OD1	ASN	265	A	<-->	8482	N	ALA	291	B	3.54
82.	2294	OD1	ASN	265	A	<-->	8484	CA	ALA	291	B	3.68
83.	2294	OD1	ASN	265	A	<-->	8486	C	ALA	291	B	3.84
84.	2294	OD1	ASN	265	A	<-->	8485	CB	ALA	291	B	3.25
85.	2294	OD1	ASN	265	A	<-->	8488	N	ALA	292	B	3.01
86.	2294	OD1	ASN	265	A	<-->	8491	CB	ALA	292	B	3.78

87.	2295	ND2	ASN	265	A	<-->	8485	CB	ALA	291	B	3.77
88.	2791	OG	SER	317	A	<-->	8477	CE1	PHE	290	B	3.44
89.	2791	OG	SER	317	A	<-->	8479	CZ	PHE	290	B	3.07
90.	3020	CG	ASN	339	A	<-->	6563	OE1	GLN	83	B	3.55
91.	3021	OD1	ASN	339	A	<-->	6563	OE1	GLN	83	B	3.44
92.	3022	ND2	ASN	339	A	<-->	6560	CB	GLN	83	B	3.56
93.	3022	ND2	ASN	339	A	<-->	6562	CD	GLN	83	B	3.64
94.	3022	ND2	ASN	339	A	<-->	6563	OE1	GLN	83	B	2.91
95.	3237	CB	SER	360	A	<-->	6568	O	GLN	83	B	3.71
96.	3238	OG	SER	360	A	<-->	6574	OD1	ASP	84	B	3.56
97.	3238	OG	SER	360	A	<-->	13849	NH2	ARG	880	B	3.27
98.	3445	C	ARG	382	A	<-->	6583	CD1	LEU	85	B	3.75
99.	3446	O	ARG	382	A	<-->	6583	CD1	LEU	85	B	3.73
100.	3433	CB	ARG	382	A	<-->	6574	OD1	ASP	84	B	3.59
101.	3434	CG	ARG	382	A	<-->	6574	OD1	ASP	84	B	3.43
102.	3434	CG	ARG	382	A	<-->	13845	CZ	ARG	880	B	3.72
103.	3434	CG	ARG	382	A	<-->	13846	NH1	ARG	880	B	3.44
104.	3434	CG	ARG	382	A	<-->	13849	NH2	ARG	880	B	3.81
105.	3435	CD	ARG	382	A	<-->	13845	CZ	ARG	880	B	3.60
106.	3435	CD	ARG	382	A	<-->	13846	NH1	ARG	880	B	3.55
107.	3436	NE	ARG	382	A	<-->	13836	O	THR	879	B	2.97
108.	3436	NE	ARG	382	A	<-->	13842	CD	ARG	880	B	3.88
109.	3436	NE	ARG	382	A	<-->	13845	CZ	ARG	880	B	3.84

110.	3436	NE	ARG	382	A	<-->	13846	NH1	ARG	880	B	3.47
111.	3438	CZ	ARG	382	A	<-->	13836	O	THR	879	B	3.40
112.	3442	NH2	ARG	382	A	<-->	13836	O	THR	879	B	3.14
113.	3456	C	ASN	383	A	<-->	6583	CD1	LEU	85	B	3.77
114.	3458	N	GLY	384	A	<-->	6583	CD1	LEU	85	B	3.22
115.	3460	CA	GLY	384	A	<-->	6583	CD1	LEU	85	B	3.73
116.	3686	O	PHE	408	A	<-->	6583	CD1	LEU	85	B	3.59
117.	3681	CD2	PHE	408	A	<-->	6586	O	LEU	85	B	3.69
118.	3681	CD2	PHE	408	A	<-->	6593	CD2	PHE	86	B	3.33
119.	3682	CE1	PHE	408	A	<-->	13836	O	THR	879	B	3.69
120.	3683	CE2	PHE	408	A	<-->	13834	CG2	THR	879	B	3.67
121.	3684	CZ	PHE	408	A	<-->	13830	CA	THR	879	B	3.67
122.	4352	CD	LYS	477	A	<-->	13940	OD1	ASN	893	B	3.30
123.	4353	CE	LYS	477	A	<-->	13940	OD1	ASN	893	B	3.23
124.	4354	NZ	LYS	477	A	<-->	13939	CG	ASN	893	B	3.83
125.	4354	NZ	LYS	477	A	<-->	13940	OD1	ASN	893	B	2.66
126.	4583	CE1	PHE	500	A	<-->	13952	CE	MET	894	B	3.58
127.	4584	CE2	PHE	500	A	<-->	13952	CE	MET	894	B	3.75
128.	4585	CZ	PHE	500	A	<-->	13946	N	MET	894	B	3.70
129.	4585	CZ	PHE	500	A	<-->	13949	CB	MET	894	B	3.90
130.	4585	CZ	PHE	500	A	<-->	13950	CG	MET	894	B	3.73
131.	4585	CZ	PHE	500	A	<-->	13952	CE	MET	894	B	3.48
132.	4596	O	LEU	501	A	<-->	13952	CE	MET	894	B	3.66

133.	4603	OD2	ASP	502	A	<-->	13948	CA	MET	894	B	3.70
134.	4603	OD2	ASP	502	A	<-->	13949	CB	MET	894	B	3.13
135.	4629	OE1	GLN	505	A	<-->	13985	CG2	ILE	897	B	3.81
136.	4630	NE2	GLN	505	A	<-->	13985	CG2	ILE	897	B	3.58
137.	4630	NE2	GLN	505	A	<-->	13987	CD1	ILE	897	B	3.66
138.	4805	CG1	VAL	524	A	<-->	13951	SD	MET	894	B	3.40
139.	4805	CG1	VAL	524	A	<-->	13952	CE	MET	894	B	3.74
140.	4821	CB	ASN	526	A	<-->	13951	SD	MET	894	B	3.48
141.	4821	CB	ASN	526	A	<-->	13952	CE	MET	894	B	3.55
142.	4824	ND2	ASN	526	A	<-->	13949	CB	MET	894	B	3.74
143.	4842	OG	SER	528	A	<-->	13994	CG	MET	898	B	3.15
144.	4842	OG	SER	528	A	<-->	13995	SD	MET	898	B	3.52
145.	4842	OG	SER	528	A	<-->	13996	CE	MET	898	B	3.59
146.	4849	CB	HIS	529	A	<-->	13996	CE	MET	898	B	3.74
147.	4851	ND1	HIS	529	A	<-->	13995	SD	MET	898	B	3.45
148.	5094	CD1	LEU	553	A	<-->	13995	SD	MET	898	B	3.68
149.	5343	OE1	GLN	578	A	<-->	14019	CD2	LEU	901	B	3.48
150.	5344	NE2	GLN	578	A	<-->	14019	CD2	LEU	901	B	3.84
151.	5603	CG	GLU	603	A	<-->	14069	NZ	LYS	906	B	3.87
152.	5604	CD	GLU	603	A	<-->	14026	CG1	VAL	902	B	3.76
153.	5604	CD	GLU	603	A	<-->	14069	NZ	LYS	906	B	2.77
154.	5605	OE1	GLU	603	A	<-->	14026	CG1	VAL	902	B	3.41
155.	5605	OE1	GLU	603	A	<-->	14068	CE	LYS	906	B	3.79

156.	5605	OE1	GLU	603	A	<-->	14069	NZ	LYS	906	B	2.71
157.	5606	OE2	GLU	603	A	<-->	14026	CG1	VAL	902	B	3.60
158.	5606	OE2	GLU	603	A	<-->	14067	CD	LYS	906	B	3.49
159.	5606	OE2	GLU	603	A	<-->	14068	CE	LYS	906	B	3.56
160.	5606	OE2	GLU	603	A	<-->	14069	NZ	LYS	906	B	2.57
161.	5622	CD	GLU	605	A	<-->	14069	NZ	LYS	906	B	3.70
162.	5622	CD	GLU	605	A	<-->	14827	OH	TYR	997	B	3.78
163.	5624	OE2	GLU	605	A	<-->	14068	CE	LYS	906	B	3.30
164.	5624	OE2	GLU	605	A	<-->	14069	NZ	LYS	906	B	2.71
165.	5624	OE2	GLU	605	A	<-->	14823	CE1	TYR	997	B	3.71
166.	5624	OE2	GLU	605	A	<-->	14826	CZ	TYR	997	B	3.64
167.	5624	OE2	GLU	605	A	<-->	14827	OH	TYR	997	B	2.84
168.	5636	NH1	ARG	606	A	<-->	14027	CG2	VAL	902	B	3.76
169.	5728	OE1	GLN	616	A	<-->	14611	CB	PRO	971	B	3.49

Salt bridges

	<----- A T O M 1 ----->						<----- A T O M 2 ----->					
	Atom no.	Atom name	Res name	Res no.	Chain		Atom no.	Atom name	Res name	Res no.	Chain	Distance
1.	40	OE2	GLU	31	A	<-->	13710	NH2	ARG	863	B	2.69
2.	5605	OE1	GLU	603	A	<-->	14069	NZ	LYS	906	B	2.57
3.	5624	OE2	GLU	605	A	<-->	14069	NZ	LYS	906	B	2.71
